# Comprehensive *in silico* CpG methylation analysis in hepatocellular carcinoma identifies tissue- and tumor-type specific marks disconnected from gene expression

**DOI:** 10.1007/s13105-024-01045-8

**Published:** 2024-09-21

**Authors:** Idoia Bilbao, Miriam Recalde, Fabrice Daian, José Maria Herranz, María Elizalde, Mercedes Iñarrairaegui, Matteo Canale, Maite G. Fernández-Barrena, Andrea Casadei-Gardini, Bruno Sangro, Matías A. Ávila, Manuel F. Landecho Acha, Carmen Berasain, María Arechederra

**Affiliations:** 1 Liver Unit and HPB Oncology Area, Clínica Universidad de Navarra, Avda. Pio XII, n55, 31008 Pamplona, Spain; 2 Hepatology Laboratory, Solid Tumors Program, CIMA, CCUN, University of Navarra, 3008 Pamplona, Spain; 3 Laboratoire d’Informatique Et Système (LIS), Aix Marseille Univ, Aix Marseille Univ, CNRS, 13009 Marseille, France; 4 Centro de Investigación Biomédica en Red de Enfermedades Hepáticas y Digestivas (CIBERehd), 28029 Madrid, Spain; 5 IdiSNA, Navarra Institute for Health Research, 31008 Pamplona, Spain; 6 Biosciences Laboratory-IRCCS Istituto Romagnolo Per Lo Studio Dei Tumori (IRST) “Dino Amadori”, 47014 Meldola, Italy; 7 Medical Oncology Department, IRCSS San Raffaele Scientific Institute, Milan, Italy; 8 Department of Oncology, Vita-Salute San Raffaele University, Milan, Italy; 9 Internal Medicine Dpt. Clínica, Universidad de Navarra, 31008 Pamplona, Spain

**Keywords:** DNA methylation, Liver cancer, Gene expression, Tumor-specific markers

## Abstract

DNA methylation is crucial for chromatin structure, transcription regulation and genome stability, defining cellular identity. Aberrant hypermethylation of CpG-rich regions is common in cancer, influencing gene expression. However, the specific contributions of individual epigenetic modifications to tumorigenesis remain under investigation. In hepatocellular carcinoma (HCC), DNA methylation alterations are documented as in other tumor types. We aimed to identify hypermethylated CpGs in HCC, assess their specificity across other tumor types, and investigate their impact on gene expression. To this end, public methylomes from HCC, other liver diseases, and 27 tumor types as well as expression data from TCGA-LIHC and GTEx were analyzed. This study identified 39 CpG sites that were hypermethylated in HCC compared to control liver tissue, and were located within promoter, gene bodies, and intergenic CpG islands. Notably, these CpGs were predominantly unmethylated in healthy liver tissue and other normal tissues. Comparative analysis with 27 other tumors revealed both common and HCC-specific hypermethylated CpGs. Interestingly, the HCC-hypermethylated genes showed minimal expression in the different healthy tissues, with marginal changes in the level of expression in the corresponding tumors. These findings confirm previous evidence on the limited influence of DNA hypermethylation on gene expression regulation in cancer. It also highlights the existence of mechanisms that allow the selection of tissue-specific methylation marks in normally unexpressed genes during carcinogenesis. Overall, our study contributes to demonstrate the complexity of cancer epigenetics, emphasizing the need of better understanding the interplay between DNA methylation, gene expression dynamics, and tumorigenesis.

**Graphical Abstract:**

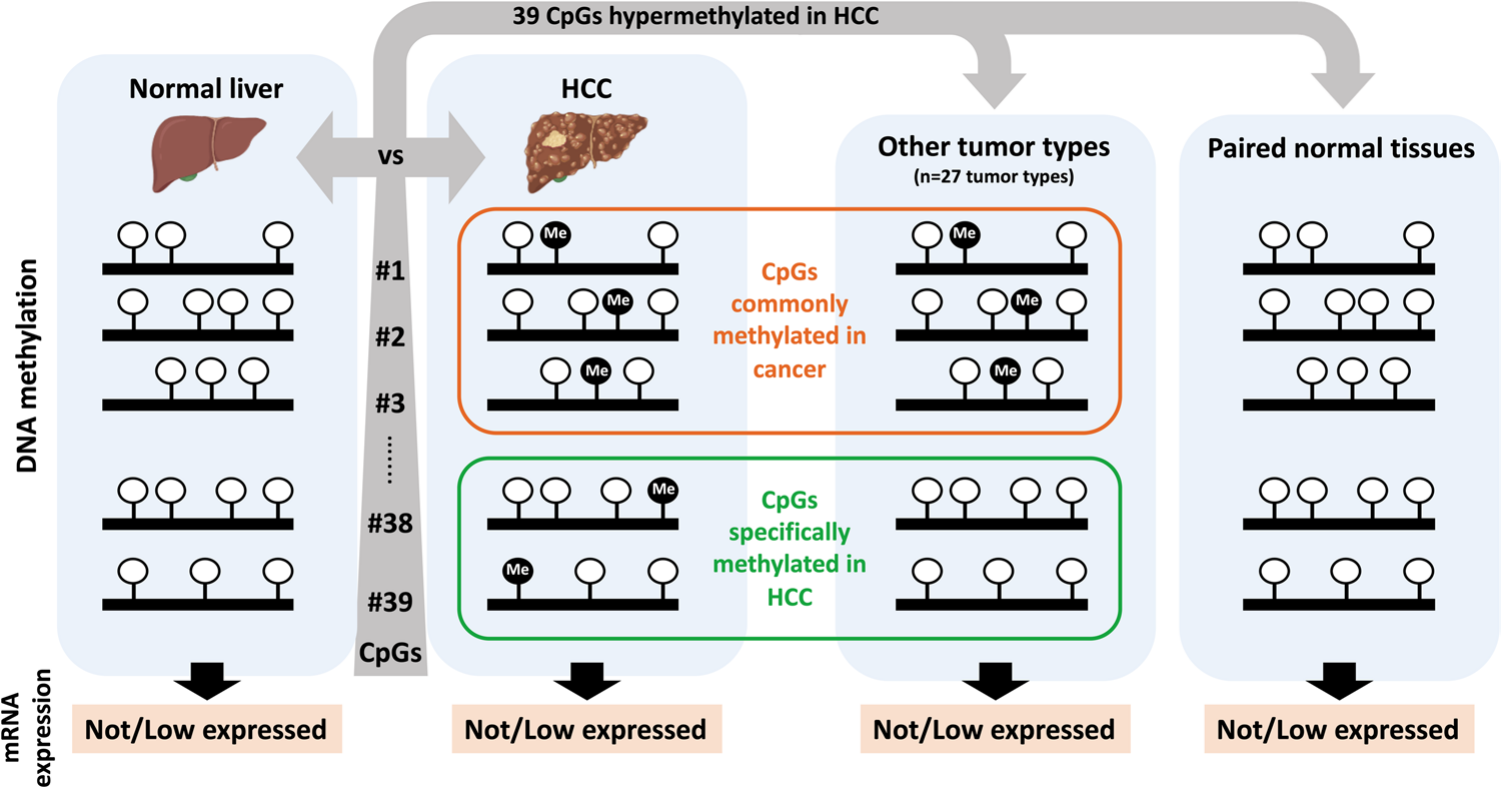

**Supplementary Information:**

The online version contains supplementary material available at 10.1007/s13105-024-01045-8.

## Introduction

DNA methylation, which consists on the addition of a methyl group at carbon 5 of cytosine in cytosine-guanine dinucleotides (CpG), is an essential epigenetic mechanism influencing chromatin structure, regulation of transcription and genome stability [26, 43]. In healthy cells, the bulk of the genome is CpG-deficient and predominantly methylated, while discrete regions called CpG islands (CGIs) are CpG-rich and usually unmethylated [24]. CGIs are located in the promoter region of approximately 60% of genes [24] and promoter-CGI methylation is associated with gene silencing [26, 43]. However approximately half of the annotated CGI are distributed between gene bodies and intergenic regions [24]. Increased CGI gene body methylation positively correlates with transcriptional activation [6, 25, 55], regulates tissue-specific promoter usage [39] or alternative splicing [38]. Therefore, methylation of CpG sites is an essential component of cell type-specific gene regulation, a fundamental mark of cell identity [12, 16, 41], and therefore the unique methylation profile of cells can be used to identify their tissue of origin [33, 59].

Importantly, alterations of methylation patterns are associated with disease development and allow to distinguish healthy from diseased states, including cancer [8, 12, 31]. In fact, an epigenetic reprogramming, including abnormal DNA methylation patterns, is a common alteration in the tumorigenic process, independently of the cell of origin [17]. It has been widely described that during the process of carcinogenesis a global hypomethylation of DNA occurs in parallel with the hypermethylation of CGIs in the promoter regions [26, 43]. Thus, although DNA methylation is considered a tissue-specific mark, many tumor suppressor genes are silenced by aberrant promoter methylation in different types of cancer [14, 26]. For instance, promoter hypermethylation of the DNA-repair gene O6-methylguanine-DNA methyltransferase (*MGMT*) is a common event in human neoplasia [13], including glioblastoma multiforme, colorectal or breast cancer or of *RASSF1A* has been observed in several types of cancer others [36], including HCC, breast, pancreatic or lung cancer, among others. However, in line with the relationship between methylation patterns and cellular identity [12, 16, 41], it has also been described that some DNA methylation patterns are associated with specific types of cancers and therefore can be used to differentiate between various tumor types [56, 59].

During the last decades, the use of high-throughput sequencing analyses has considerably contributed to our understanding of how epigenetic modifications contribute to the tumorigenic process [14]. However, an open question remains as to which of all the DNA methylation modifications that occur during malignant transformation are common across different types of tumors (how many and which types of tumors) or specific to a single tumor type. This includes not only the CpGs within promoter CGIs but also those located in gene bodies and intergenic regions, even outside of CGIs. Furthermore, it is critical to determine the impact of such hypermethylation on gene expression and the tumorigenic process, distinguishing between modifications that act as tumorigenic drivers and those that are merely passenger events [10, 27].

Hepatocellular carcinoma (HCC), the most frequent form of liver cancer, ranks as the sixth most common neoplasm and the fourth leading cause of cancer-related death [32]. HCC development and progression consists in a multistep and complex process with sequential genetic and epigenetic alterations [15, 32]. Several previous studies have reported human DNA methylome data on HCC tissue samples and their non-tumoral liver tissue counterparts [3, 20, 30, 40, 44, 50].

Here, using publicly available methylomes, we identified a set of 39 CpGs hypermethylated in HCC compared to control liver that were located not only in promoter but also in gene bodies and intergenic CGIs. These CpGs were predominantly unmethylated in control liver and other extrahepatic healthy tissues, allowing us to identify CpGs that were hypermethylated exclusively in HCC or in several other tumors. Importantly, we observed that DNA hypermethylation in tumors occurs in genes either not expressed or minimally expressed in healthy tissues, suggesting a potential role of DNA methylation in gene expression regulation and cancer development.

## Methods

### Human samples

The study was approved by the Human Research Review Committee of the University of Navarra (CEI 47/2015). Samples from tumoral (n = 19) and non-tumoral (n = 5) tissues were obtained from HCC patients treated with liver resection. Healthy liver tissues (n = 5) were obtained from surgical resections performed to patients with colon cancer metastasis with normal or minimal liver changes. Samples and data from patients included in the study were provided by the Biobank of the University of Navarra and were processed following standard operating procedures approved by the Ethical and Scientific Committees and by Dr. A Casadei-Gardini (Colection ref.IRST-BO24). Informed consent was obtained from each patient and the study protocol conformed to the ethical guidelines of the 1975 Declaration of Helsinki.

### Public methylome datasets

All methylomes used were carried out with the Illumina's Infinium HumanMethylation450 (HM450) BeadChip array that analyze around 450.000 CpGs. The CpG candidate lists were identified from: GSE56588 [50], GSE54503 [44], TCGA-LIHC [3], and GSE40279 [18]. For non-tumor liver diseases, we utilized datasets GSE61258 [22], GSE48325 [1], and GSE60753 [20]. To validate findings in human HCC tissue samples, we used datasets GSE60753 [20], GSE89852 [30], and GSE157341 [40]; and GSE60753 [20] for primary hepatocytes and HCC cell lines (FLNEO, H801, HCO2, Hep3B, Huh75, LH86, SNU423, SNU449). Methylation data from 28 tumor types from the TCGA database (including TCGA-LIHC; Supplementary Table S4), comprising 8706 tumor and 759 non-tumor samples, were included.

### Identification of differentially methylated CpGs

For identifying hypermethylated CpGs in HCC, 485.512 single CpG sites, present in all samples from GSE56588 [50], GSE54503 [44] and TCGA-LIHC [3] were analyzed. Of these, 144,543 CpGs located within a CGI were retained. β-value ranged from 0 (unmethylated) to 1 (fully methylated). The methylation difference per CpG was calculated as the mean β-value of tumors minus controls. CpGs with a mean methylation difference > 0.2 and an FDR < 0.05 (Student’s two-sided T-test and Benjamini–Hochberg False Discovery Rate) were retained. CpGs are classified as “hypomethylated” with a difference is <  − 0.2 and as “hypermethylated” with a difference > 0.2.

For each study, the β-value of the 39 CpGs of interest was extracted from their database. A CpG was considered unmethylated when its β-value was < 0.2 and methylated when its β-value was > 0.2. Each figure includes a color scale indicating the β-value. Additionally, where indicated, the methylation difference between groups was calculated as explained above. Hypermethylation was considered when the difference was > 0.2.

### Expression in databases

The expression of the genes was analyzed in HCC and non-tumoral liver samples from the TCGA-LIHC dataset (raw RNA-Seq data downloaded from the GDC Data Portal) and in normal tissues from GTEx.

### Hierarchical clustering

Unsupervised hierarchical clustering was performed using the linkage function from the Python Scipy library with Ward distance and plotted using the dendogram function.

### Dimensionality reduction analyses

Uniform Manifold Approximation and Projection (UMAP) of patients CGI methylation across studies using umap Python library.

### Total DNA Isolation

Total DNA from frozen tissues was isolated using the Maxwell® RSC Cultured Cells DNA Purification Kit with a Maxwell® RSC 48 instrument (Promega, Madison, WI, USA; AS1620). DNA purity and concentration were measured using a NanoDrop spectrophotometer (Thermo Fisher Scientific, Waltham, MA, USA).

### PCR and Sanger sequencing

Genomic DNA (1 µg) was bisulfite converted using the EZ DNA Methylation Gold Kit (Zymo Research; D5005) according to the manufacturer’s instructions. PCRs of fragments containing the CpG candidates in bisulfite-converted DNA were performed using 5 ng of bisulfite-converted DNA, the Phusion U Hot Start DNA Polymerase kit (F-555S, Thermo Fisher Scientific), and specific primers. PCR products were electrophoresed and visualized in GelRed Nucleic Acid (Biotium, Fremont, CA, USA) stained gels (2% agarose) under UV light. The resulting bands were sequenced by Sanger and quantified by a modified version of EditR [29]. Primers will be provided upon request.

### Statistical analysis

Statistical analysis was performed using GraphPad Prism software. A descriptive analysis was done to analyze the sample distribution with a D’Agostino normality test. A two-sided unpaired Student’s t-test or Mann–Whitney U-test were used according to sample distribution.

## Results

### In silico identification of CpGs hypermethylated in HCC

**Fig. 1:**
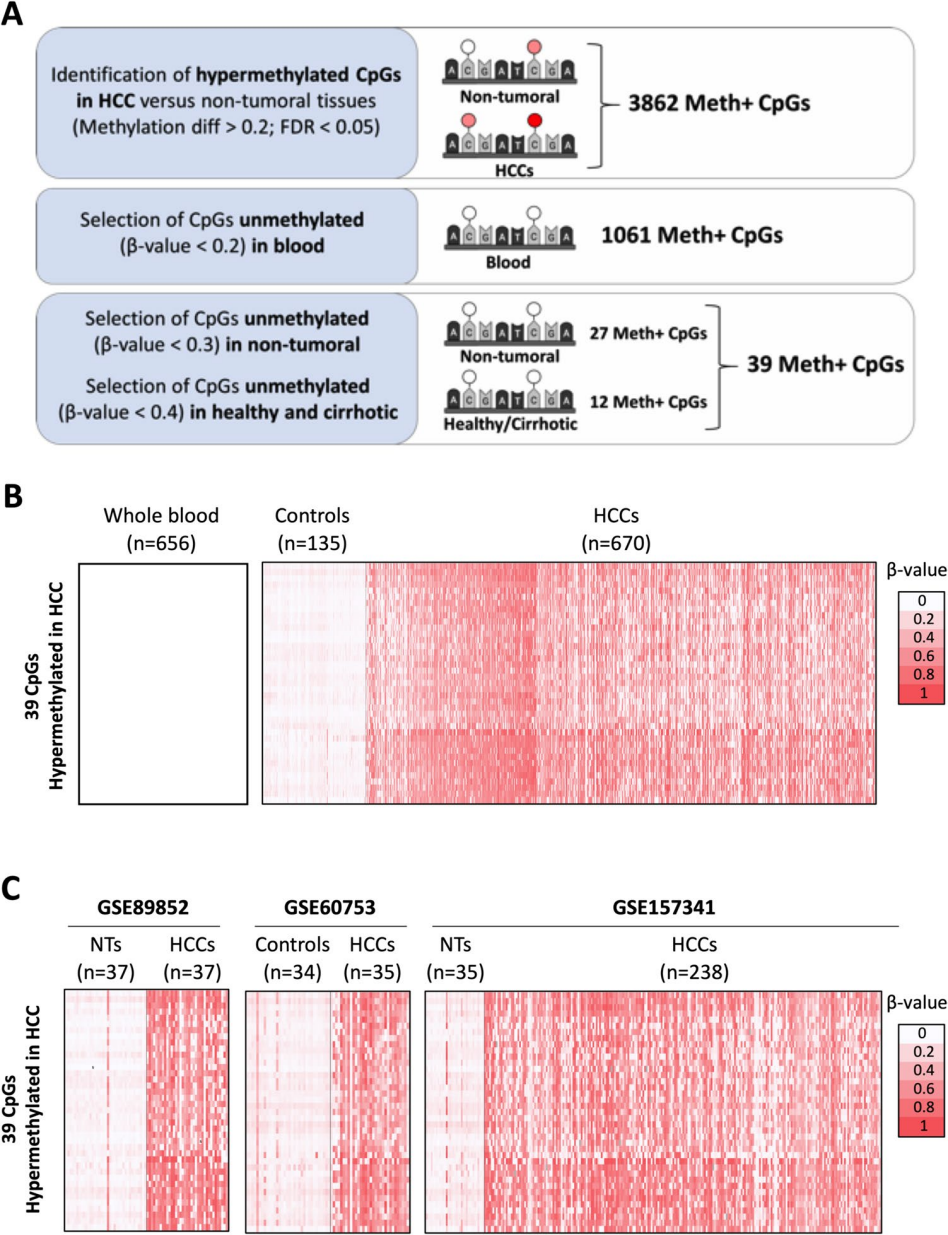
In silico identification of CpGs hypermethylated in HCC. **A** Schematic representation of the workflow followed to identify the list of 39 CpGs hypermethylated in HCC but unmethylated in control liver, cirrhotic tissue or whole blood. **B**, Heatmaps reporting the methylation levels of the 39 CpGs across GSE56588, GSE54503, TCGA-LIHC, GSE40279 datasets, involving 656 healthy individuals' whole blood, 135 liver controls, and 670 HCCs. The 39 CpGs are ranked from the highest to lowest mean methylation difference. **C** Validation datasets GSE89852, GSE60753 and GSE157341. The CpGs are ranked as in panel B. Color scale bar is shown for reference

To identify CpGs differentially methylated in HCC, we used three available liver tissue methylomes: GSE56588 (224 HCC, 9 cirrhotic livers, 10 controls), GSE54503 (66 HCC, 66 peritumoral liver samples), and TCGA-LIHC (380 HCC and 50 peritumoral liver samples), all analyzed using Illumina's Infinium HumanMethylation450 BeadChip array (Supplementary Figure S1A). Hierarchical clustering distinguished two groups, separating controls from patients across all studies (Supplementary Figure S1B). UMAP dimensionality reduction similarly clustered HCC and control liver samples together (Supplementary Figure S1C), indicating minimal batch effect. We therefore decided to integrate the methylome data of the three datasets resulting in 670 HCCs and 135 controls. Focusing on CGIs, and consistent with the literature [15], we observed global hypermethylation enrichment in HCC patients versus controls (p-value = 4.31E-28; Supplementary Figure S1D). We identified 3862 CpGs hypermethylated in HCC compared to controls, with a mean methylation difference > 0.2 and FDR < 0.05 (Student's two-sided T-test with Benjamini–Hochberg correction; Fig. 1A, Supplementary Table S1).

To identify those CpGs methylated mainly in HCC cells we eliminated marks potentially originating from circulating blood cells. We excluded all CpGs methylated (β-value > 0.2) in the whole blood of at least one of the 656 healthy individuals covering a wide age range (aged 19 to 101) analyzed in GSE40279. The resulting list was reduced to 1061 CpGs methylated in HCC tissues but not in any of the blood samples (Fig. 1A, Supplementary Table S2). Moreover, none of these 1061 CpGs were part of the 353 CpG sites constituting the aging clock [21], ruling out the possibility of an age-related methylation effect in the identified CpGs.

From the 1061 CpGs hypermethylated in hepatic cells, we identified (a) 27 CpGs unmethylated (β-value < 0.2) in all 135 non-tumoral samples (healthy, cirrhotic and adjacent non-tumoral tissue) with a methylation difference > 0.3 in HCC; moreover, (b) 12 CpGs unmethylated (β-value < 0.2) in livers not harboring a tumor (all the healthy and cirrhotic patients) and methylated in liver tissue adjacent to HCC and in HCC, with a difference > 0.4. Finally, we selected a set of 39 CpGs that were hypermethylated (β-value > 0.3) in HCC but unmethylated (β-value < 0.2) in control liver, cirrhotic tissue or whole blood (Fig. 1B**, **Supplementary Table S3).

Supporting an association with malignant transformation rather than with liver damage development, none of the 39 CpGs were hypermethylated in livers from obese, NAFLD, NASH, primary biliary cirrhosis, primary sclerosing cholangitis, or cirrhotic patients (GSE61258, GSE48325 and GSE60753; Supplementary Figure S2A).

Accordingly, using independent available methylomes (GSE60753, GSE89852 and GSE157341) we confirmed that all 39 CpGs were hypermethylated in HCC tissues compared to non-tumoral samples (Fig. 1C). Indeed, each CpGs was methylated in at least 54% of the 977 HCC tissues analyzed, ranging from 54.70% to 84.25% (Supplementary Figure S2B).

**Fig. 2:**
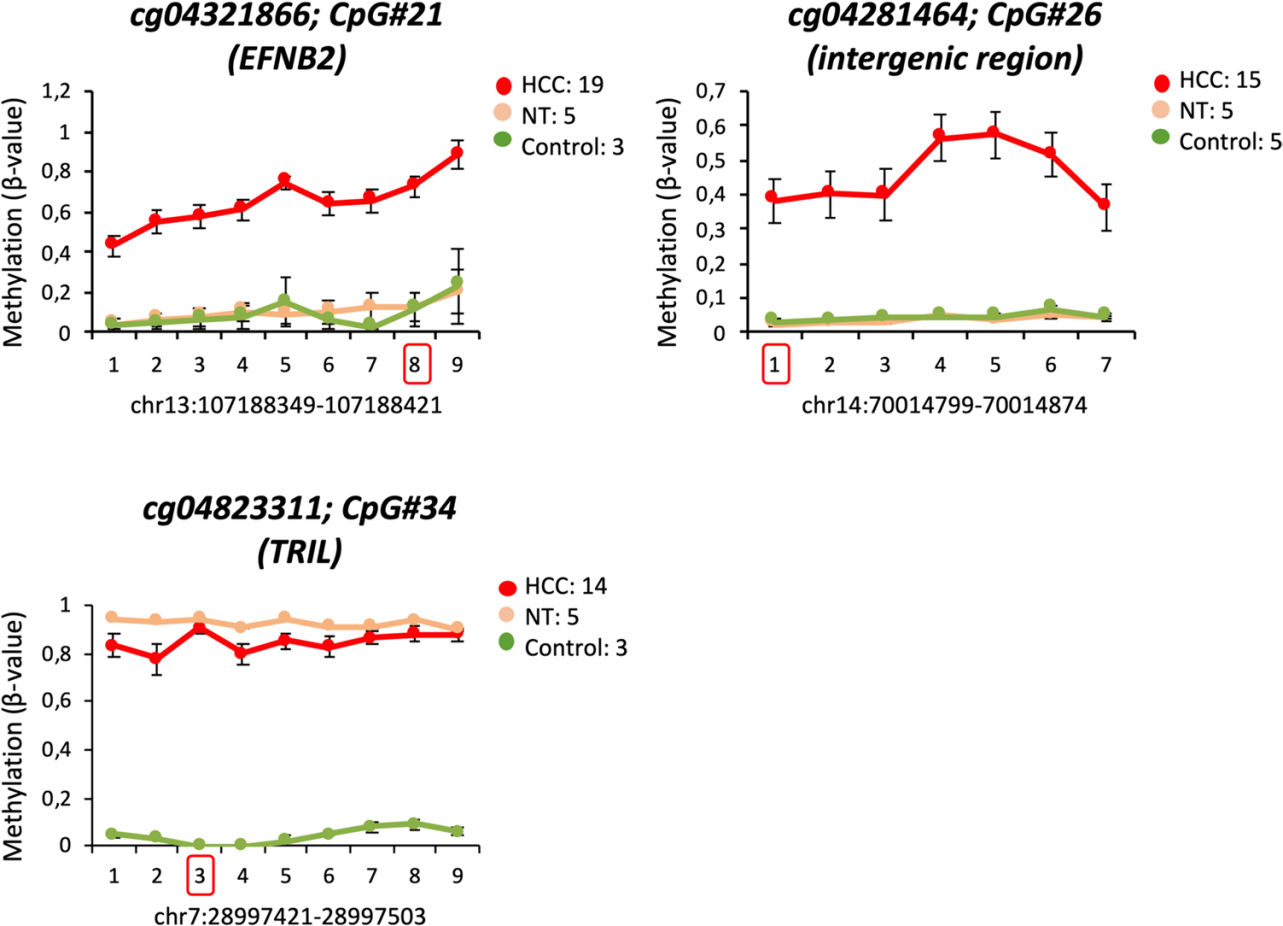
Hypermethylation of three candidate CpGs in human HCC and control liver tissues. Graphs show *β-values* of cg04321866 (CpG#21; *EFNB2*), cg04281464 (CpG#26; intergenic region), and cg04823311 (CpG#34; *TRIL*) across HCC samples (n = 19), non-tumoral tissues (NT, n = 5), and control livers (n = 5). PCR amplification and Sanger sequencing were used to analyze methylation across all CpGs within the DNA fragment, with the candidate CpG indicated by a red square

Moreover, we experimentally confirmed the higher methylation level of three selected CpGs in different HCC samples compared to control liver and paired non-tumoral tissues. Consistent with our criteria, two CpGs (cg04321866 and cg04281464) were hypermethylated in HCC compared to both healthy liver and non-tumoral tissue, while the third CpG (cg04823311) was hypermethylated in both HCC and non-tumoral tissue but not in healthy liver samples (Fig. 2). Interestingly, this validation revealed that not only the CpG interrogated in the Infinium array and selected in silico, but all CpGs within each PCR-amplified fragment were hypermethylated in HCC samples (Fig. 2). This result suggests that hypermethylation of these CpGs may play a role in HCC development.

### Differential methylation of the CpGs hypermethylated in HCC in other tumors

**Fig. 3:**
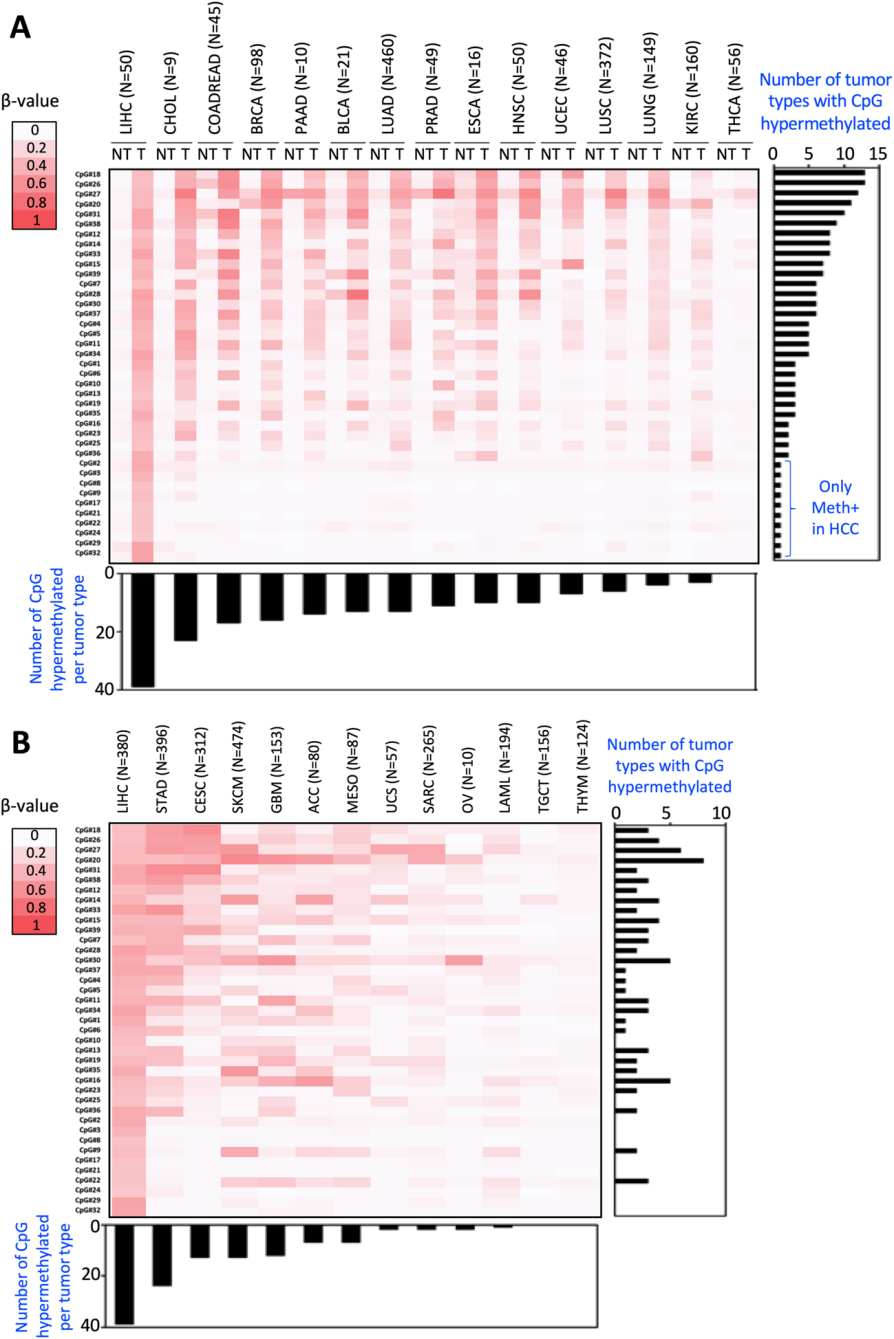
Methylation levels of the identified hypermethylated CpGs in HCC across various non-tumoral and tumoral samples from different tumor types. **A** Heatmap displays the mean methylation levels of the 39 CpGs in non-tumoral (NT) and tumoral tissues (T) from TCGA datasets, including LIHC (Hepatocellular Carcinoma), CHOL (Cholangiocarcinoma), COADREAD (Colorectal Adenocarcinoma), BRCA (Breast Invasive Carcinoma), PAAD (Pancreatic Adenocarcinoma), BLCA (Bladder Urothelial Carcinoma), LUAD (Lung Adenocarcinoma), PRAD (Prostate Adenocarcinoma), ESCA (Esophageal Carcinoma), HNSC (Head and Neck Squamous Cell Carcinoma), UCEC (Uterine Corpus Endometrial Carcinoma), LUSC (Lung Squamous Cell Carcinoma), LUNG (Pan-Lung Cancer), KIRC (Kidney Renal Clear Cell Carcinoma), and THCA (Thyroid Carcinoma). CpGs are ranked by the number of tumors showing a mean methylation difference greater than 0.2 (from most to least), and tumors are organized from left to right based on the number of CpGs hypermethylated (from most to least). **B** Heatmap reporting the mean methylation level of the same CpGs in 13 tumor types with only methylation data of the tumoral tissues (T) from TCGA: LIHC, STAD (Stomach Adenomarcinoma), CESC (Cervical Squamous Cell Carcinoma and Endocervical Adenocarcinoma), SKCM (Skin Cutaneous Melanoma), GBM (Glioblastoma Multiforme), ACC (Adrenocortical Carcinoma), MESO (Mesothelioma), UCS (Uterine Carcinosarcoma), SARC (Sarcoma), OV (Ovarian Serous Cystadenocarcinoma), LAML (Acute Myeloid Leukemia), TGCT (Testicular Germ Cell Tumors) and THYM (Thymoma). CpGs are ranked as in panel A, and tumors are arranged based on the number of hypermethylated CpGs (from most to least) from left to right. The color scale bar is provided on the left side

At this point we wondered whether the methylation of these CpGs was a specific feature of HCC cells (Supplementary Figure S2C), or a general characteristic of cancer cells. To address this issue, we first examined the methylation status of the 39 CpGs in healthy tissues to establish their baseline physiological methylation status. We took advantage of 14 TCGA methylomes with more than 5 peritumoral samples (759 non-tumoral samples distributed across 12 different tissues, in addition to liver; Supplementary Table S4). Thirty-two of the 39 CpGs showed no methylation (mean β-value < 0.2) in any of the tissues examined (Fig. 3A). Additionally, six CpGs (CpG#20, CpG#26, CpG#28, CpG#31, CpG#33, and CpG#39) exhibited methylation (mean β-value > 0.2) in only one of the analyzed tissues, while one CpG (CpG#27) was methylated (mean β-value > 0.2) in five tissues (Fig. 3A). These results suggest that, similar to liver and whole blood, these CpGs are predominantly unmethylated in healthy tissues.

Considering that these CpGs were practically unmethylated in healthy tissues, we further examined whether these CpGs were hypermethylated in their corresponding tumor tissues. Our analysis revealed that five CpGs (CpG#18, CpG#26, CpG#27, CpG#20 and CpG#31) were hypermethylated in 10 or more tumor types compared to their corresponding control tissues (Fig. 3A). This suggests that the methylation of these CpGs may be a widespread change in carcinogenesis. Conversely, ten CpGs (CpG#2, CpG#3, CpG#8, CpG#9, CpG#17, CpG#21, CpG#22, CpG#24, CpG#29, and CpG#32) showed differential methylation exclusively in HCC (Fig. 3A), suggesting that they represent a specific methylation signature of HCC.

In terms of tumor types, cholangiocarcinoma, followed by colorectal adenocarcinoma, breast carcinoma, and pancreatic adenocarcinoma, exhibited the highest number of hypermethylated CpGs compared to their corresponding controls (23, 17, 16 and 14 CpGs out of the 39, respectively; Fig. 3A). In contrast, lung squamous cell carcinoma, lung adenocarcinoma, and kidney renal clear cell carcinoma were the tumors with fewer hypermethylated CpGs compared to control samples (6, 4 and 3, respectively; Fig. 3A). Notably, thyroid carcinoma did not show hypermethylation of any of the examined CpGs (Fig. 3A).

Additionally, the methylation status of these 39 CpGs was assessed across 13 tumor types from TCGA database where non-tumor sample methylation data were unavailable (Supplementary Table S4). While differential methylation compared to controls could not be confirmed, their methylation status in tumor tissues was established. The five CpGs identified as hypermethylated in more than ten tumors (CpG#18, CpG#26, CpG#27, CpG#20 and CpG#31; Fig. 3A) were methylated in three, four, six, eight and two of these tumor types, respectively (Fig. 3B). Importantly, among the ten CpGs exclusively hypermethylated in HCC (CpG#2, CpG#3, CpG#8, CpG#9, CpG#17, CpG#21, CpG#22, CpG#24, CpG#29, and CpG#32; Fig. 3A), eight (CpG#2, CpG#3, CpG#8, CpG#17, CpG#21, CpG#24, CpG#29, and CpG#32) were not methylated in any of the 13 tumor types analyzed (Fig. 3B), reinforcing their specific methylation pattern in HCC. The remaining two CpGs (CpG#9 and CpG#22) were methylated (mean β-value > 0.2) in two and three tumors, respectively (Fig. 3B).

In terms of tumor types, stomach adenocarcinoma, cervical squamous cell carcinoma and endocervical adenocarcinoma, skin cutaneous melanoma, and glioblastoma multiforme showed methylation (mean β-value > 0.2) in more than ten CpG sites (Fig. 3B). Conversely, uterine carcinosarcoma, sarcoma, ovarian serous cystadenocarcinoma, and acute myeloid leukemia exhibited methylation in only two CpG sites, while testicular germ cell tumors and thymoma did not display methylation in any of the CpG sites analyzed (Fig. 3B).

In summary, our results indicate that most of the CpGs hypermethylated in HCC are predominantly unmethylated not only in the normal liver and blood but in the majority of healthy tissues. Additionally, we identified a subset of CpGs specifically hypermethylated in HCC, while many others are hypermethylated across multiple tumor types. Particularly noteworthy are five CpGs hypermethylated in 10 or more tumor types, indicating a non-random mechanism potentially involved in carcinogenesis.

### HCC hypermethylated CpGs and gene expression

**Fig. 4:**
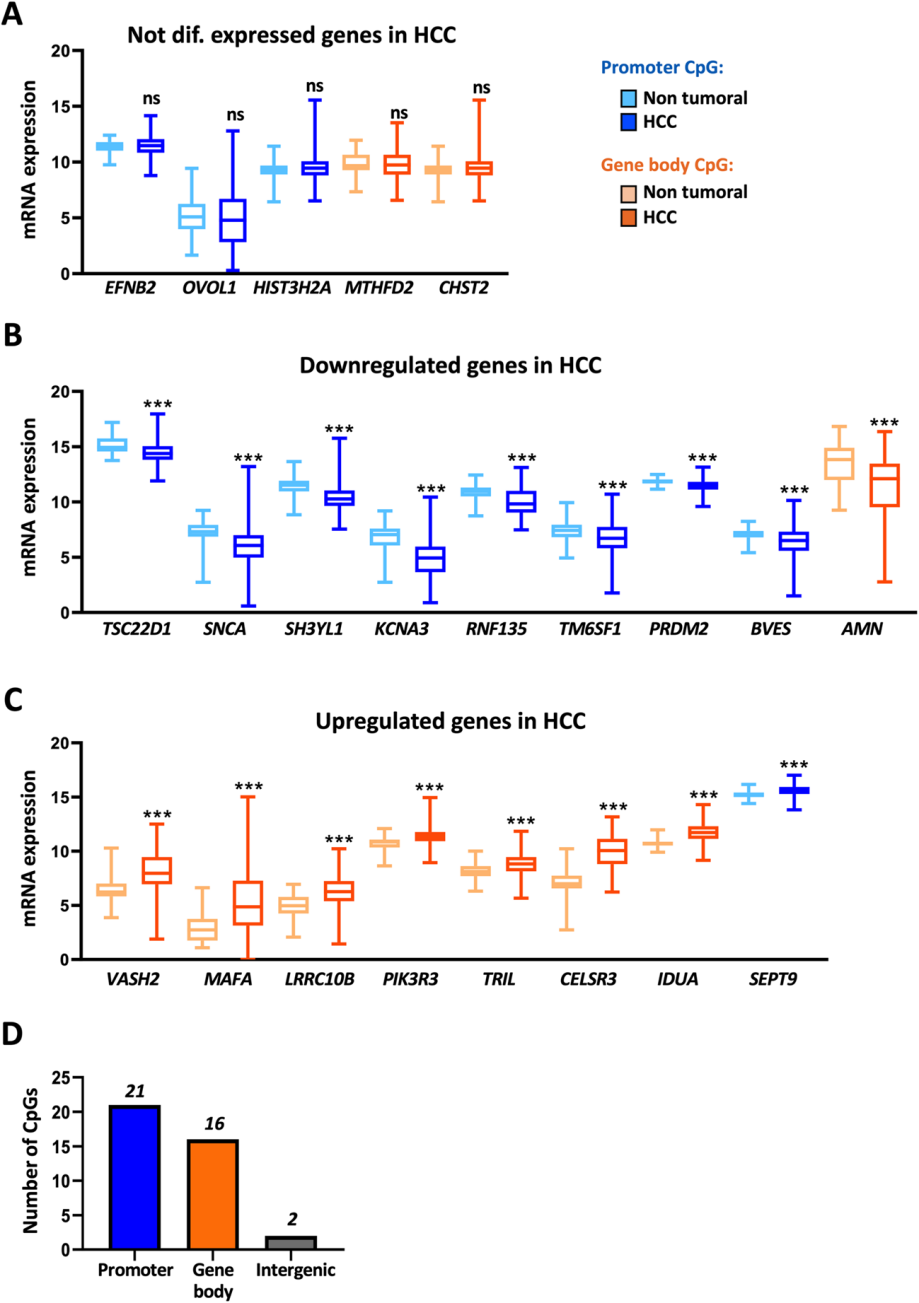
Correlation of DNA methylation, CpG location and gene expression. **A**-**C** Graphs show gene expression levels (log CPM from RNA-seq data) of genes that are not differentially expressed (**A**), downregulated (**B**), or upregulated (**C**) in hepatocellular carcinoma (HCC) compared to non-tumoral tissue, based on TCGA-LIHC data (50 NT and 380 HCC samples). Statistical significance (***p < 0.001) is indicated for each comparison. (**D**) Distribution of the 39 CpGs categorized by their location in promoters (blue), gene bodies (orange), or intergenic regions (grey) within CpG islands (CGIs). The promoter region includes 1500 nucleotides upstream of the transcription start site (TSS1500) to 300 nucleotides downstream of the TSS

In order to have a general view of the impact of CpG hypermethylation on gene expression, we decided to examine the expression levels of the genes containing the selected CpGs in tumor HCC samples and adjacent control liver tissues from the TCGA-LIHC database. The 39 CpG sites were located in 32 distinct genes and two intergenic regions not uniformly distributed across the genome (Supplementary Table S3), as they were predominantly found on the larger chromosomes, with six of them in chromosome 1 (Supplementary Figure S3). Out of the 32 genes, ten genes (*MIXL1*, *INSM2*, *HIST1H3F*, *INA*, *PYY*, *MIR129-2*, *HOXA11*, *PITX3*, *CELF6* and *MCIDAS*) were not expressed in either control liver or HCC tissue samples and five genes (*EFNB2*, *MTHFD2*, *OVOL1, HIST3H2A* and *CHST2*) showed no differences in expression between HCC and non-tumoral samples (Fig. 4A). However, the other 17 genes were significantly differentially expressed (Fig. 4B-C). The expression of nine genes (*TSC22D1*, *SNCA*, *SH3YL1*, *KCNA3*, *RNF135*, *TM6SF1*, *PRDM2, BVES* and *AMN*) was reduced (Fig. 4B), while that of eight genes (*VASH2*, *MAFA*, *LRRC10B*, *PIK3R3*, *TRIL*, *CELSR3*, *IDUA* and *SEPT9*) was increased (Fig. 4C) in HCC compared to control livers. Of note, although these changes were statistically significant, in most cases the differences in the expression levels between control livers and HCC tissues were really small, questioning their biological relevance.

In order to gain mechanistic insight regarding these seemingly arbitrary patterns of gene expression modulation, we decided to examine the location of the CpG sites along the respective gene sequence. It is well-known that promoter CGI hypermethylation represses transcription [43, 45], whereas hypermethylation at gene body CGIs activates gene expression [6, 55]. We found that in addition to the two CpGs located in intergenic regions, 21 CpGs were located within promoter CGIs, while the remaining 16 were within gene body CGIs (Fig. 4D, Supplementary Table S3). In agreement with the literature, genes whose expression was downregulated in HCC had the hypermethylated CpGs located in promoter CGIs, whereas the upregulated genes had hypermethylated CpGs located in gene body CGIs. We observed two exceptions, *AMN* and *SEPT9*, whose CpGs were located in the gene body and the promoter CGIs respectively but their expression was reduced and induced (Fig. 4B-C**, **Table 1). Remarkably, within the genes that were not expressed and those that showed no difference in expression between non tumoral and HCC samples, we found CpG sites in CGIs located in both promoter and gene body regions (Fig. 4A, Table 1). This result highlights a complex interplay between DNA methylation, gene expression, and tumorigenesis in HCC, suggesting that other regulatory mechanisms may influence the expression of these genes and that the role of differential methylation in these regions remains to be determined.

**Table 1:** Table reporting the number of CpGs and genes hypermethylated in HCC versus non-tumoral tissue from the TCGA-LIHC database

				Nº genes expressed		
	Nº CpGs	Nº genes	Nº genes not expressed in liver & HCC	Not diff exp HCC/Liver	Downreg HCC/Liver	Upreg HCC/Liver
Total	39	32	10	5	9	8
Promoter	21	17	5	3	8	1
Gene body	16	15	5	2	1	7
Intergenic	2	0				

### Genes specifically hypermethylated in HCC were not expressed, or were expressed at low levels, in the normal liver

**Fig. 5:**
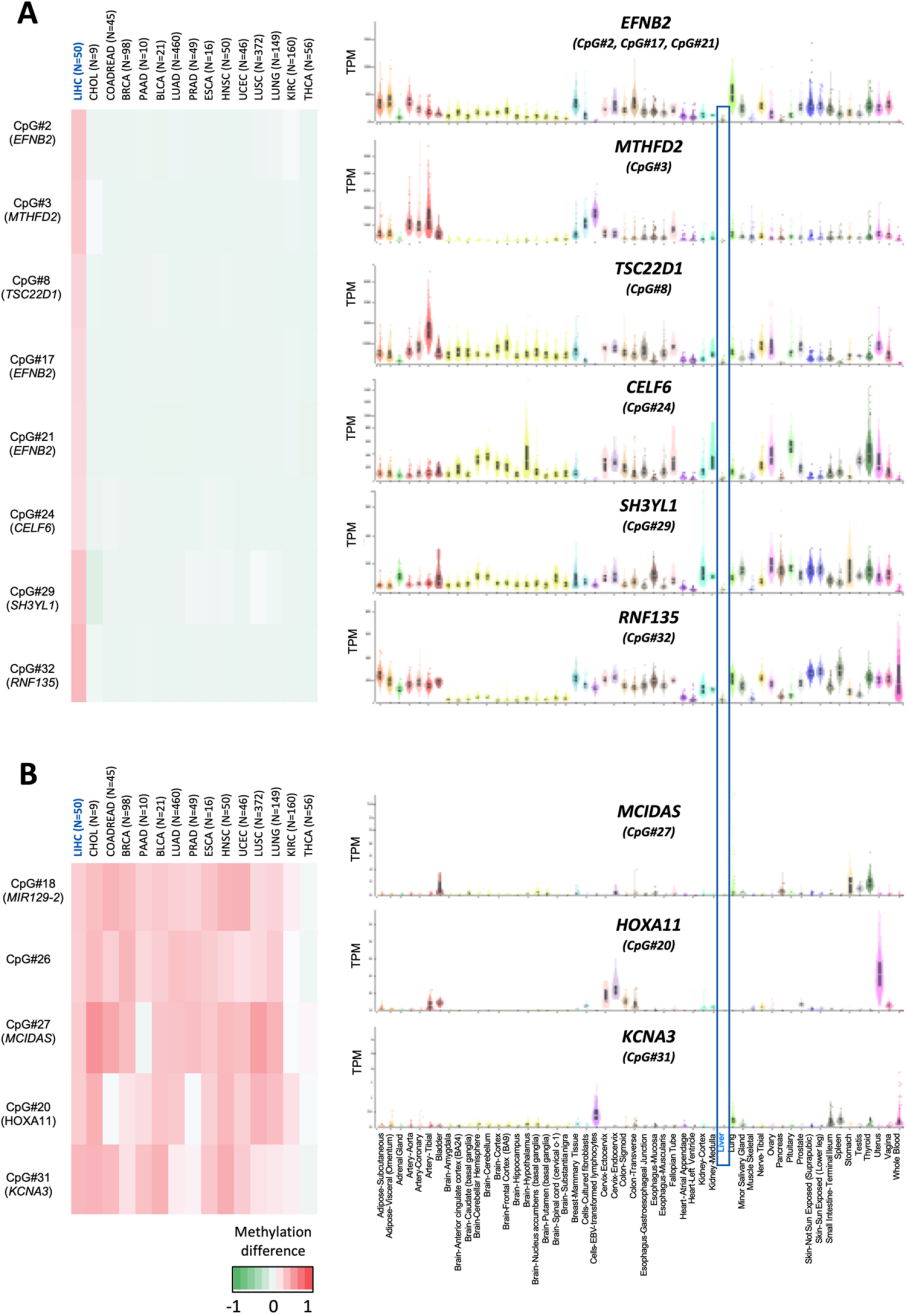
Methylation and expression of genes specifically hypermethylated in HCC or in 10 or more tumors. **(Right)** Heatmap showing the mean methylation difference of the eight CpGs specifically hypermethylated in HCC (*EFNB2*, *MTHFD2*, *TSC22D1*, *CELF6*, *SH3YL1* and *RNF135)* (**A**) or the five CpGs hypermethylated in 10 or more tumors (*MIR129-2*, intergenic region, *MCIDAS*, *HOXA11* and *KCNA3)* (**B**) The color scale bar is provided below. **(Left)** Tissue-wide gene expression profiles across 54 tissues from GTEx are shown in TPM, with the liver highlighted by a blue square

At this point, focusing on the six genes that are specifically hypermethylated in HCC (Fig. 5A**-right**), and noting that one of them is not expressed in either liver or HCC (*CELF6*), two others are not associated with changes in expression (*EFNB2* and *MTHFD2*), and the remaining three are slightly downregulated in HCC (*TSC22D1*, *SH3YL1* and *RNF135*), we questioned whether this hypermethylation status may indeed have an impact on gene expression. In this regard, while a substantial body of literature supports the association of DNA methylation with the regulation of gene expression and its implication in tumor progression [6, 26, 45, 55], it has also been proposed that the majority of hypermethylated genes in cancer are genes already transcriptionally repressed in the pre-cancerous tissue [48, 49]. Moreover, it has been shown that genes specifically repressed in one tissue are prone to be hypermethylated in the corresponding tumor, which may help distinguishing the tissue of origin of the tumor [48, 49]. To investigate this, we examined the expression of these genes in control liver tissue and compared it with their expression in other tissues using the GTEx database. We found that the expression of the genes hypermethylated in HCC (Fig. 5A**-right**) was markedly lower in liver tissue compared to other healthy tissues (Fig. 5A**-left**), suggesting that these genes specifically hypermethylated in HCC are also specifically repressed in the normal liver.

To confirm if as proposed genes hypermethylated in cancer correspond to genes little expressed in the corresponding non-tumoral tissue [48, 49], we decided to examine the expression of the five genes that were hypermethylated in 10 or more tumors (Fig. 5B-right) in different healthy tissues (Fig. 5B-left). Among the five CpGs hypermethylated in 10 or more tumors, only three correspond to coding genes (*MCIDAS*, *HOXA11* and *KCNA3*). We found that, in agreement with the correlation found in the genes specifically methylated in HCC, these three genes are generally not expressed in healthy tissues (Fig. 5B-left). Interestingly, as mentioned above these three genes are not hypermethylated in thyroid carcinoma (THCA) and at least two of them are expressed in the normal thyroid tissue. Altogether, these findings are in line with the emerging notion that genes normally repressed in a healthy tissue become hypermethylated in the corresponding tumor, a situation that would be at odds with the commonly recognized role of DNA hypermethylation in gene expression regulation.

## Discussion

DNA methylation is a key epigenetic modification that regulates gene expression and plays an essential role in cancer development [17, 26]. The explosion of high-throughput analyses in recent decades, including methylome studies, has generated massive amount of data from numerous patients across centers and countries [4]. In oncology, these data have significantly enhanced our understanding on the mechanisms underlying tumorigenesis and facilitated the identification of new therapeutic targets and diagnostic biomarkers [4]. Moreover, the free availability of these datasets enables further analyses, helping to increase our knowledge. However, in general, studies are focused on events within a particular tumor type, leaving uncertainty about whether these changes are specific to that tumor or associated with the general carcinogenesis process. Additionally, DNA methylation changes have been typically studied in gene promoters in association with gene expression alterations, rather than considering a broader view of differential DNA methylation at any CpG site across the genome.

In this study, we identify a set of 39 CpGs located within CGIs in promoter, gene body and intergenic regions that are hypermethylated in HCC. Importantly, our analysis revealed that the 39 CpGs were unmethylated not only in the normal liver and blood, but in hepatic tissues from different liver diseases, supporting the association of these methylation marks with the malignant transformation of the liver rather than with the development of liver damage. We evaluated whether the hypermethylation of the 39 CpGs was common to different tumor types or if it was HCC-specific. The TCGA project includes methylation data on 28 tumor types, with more than 8500 tumor and 750 peritumoral samples, allowing to study whether the methylation patterns identified are tissue-specific, cancer-related or cancer-specific. This analysis firstly revealed that the 39 CpGs were also unmethylated in most non-tumoral tissues, being therefore ubiquitously unmethylated CpGs in physiological conditions. When we analyzed whether these 39 CpGs were methylated in other tumors, we identified a group of methylation sites that were common to many tumor types, while others were specific to HCC. Of the CpGs methylated in numerous tumors, five (CpG#18, CpG#26, CpG#27, CpG#20 and CpG#31) were notably hypermethylated in ten or more tumor types analyzed. CpG#26 was located in an intergenic region and the other four CpGs were located in the *MIR129-2*, *MCIDAS, HOXA11/HOXA11-AS* and *KCNA3* genes. In agreement with our findings, *MIR-129–2* has been reported as hypermethylated not only in HCC [34], but also in gastric [2], breast [37] or esophageal cancer [35]. *HOXA-11* was found hypermethylated in HCC [23] and also in lung [57], glioblastoma [46], or endometrial [53] carcinoma. Similarly, *KCNA3* was shown as hypermethylated in esophageal squamous cell carcinoma [9], colorectal cancer [7] or oropharyngeal squamous cell carcinoma [42]. However, *MCIDAS* methylation has been only associated with colorectal cancer, being shown hypermethylated in serrated polyps, likely colorectal cancer precursors [5], and identified as one of the 23 DNA-methylation sites proposed to predict progression in patients with colorectal cancer [11]. In our analysis, we found hypermethylation of *MCIDAS* in HCC, cholangiocarcinoma, and colorectal, breast, prostate, pancreas, lung, esophagus or head and neck carcinomas, among others.

Additionally, our study reveals eight CpGs specifically hypermethylated in HCC and located in six genes, *EFNB2* (CpG#2, CpG#17, CpG#21), *MTHFD2* (CpG#3), *TSC22D1* (CpG#8), *CELF6* (CpG#24), *SH3YL1* (CpG#29) and *RNF135* (CpG#32). All these genes, except *CELF6*, were also identified as hypermethylated in HCC by Song et al. [47]. Moreover, *EFNB2* [19] and *RNF135* [51] were described hypermethylated in HCC in two additional studies. In contrast, in another study *CELF6* was found hypomethylated instead of hypermethylated in HCC [54]. However, and according to our data, there are no studies describing the methylation of these genes in other tumors, with the exception of one publication describing the hypermethylation of *CELF6* in oral squamous cell carcinoma [28] and that of *MTHFD2* in esophageal carcinoma [52]. Altogether, and in agreement with previous reports [58], our analysis identified common and tumor-specific epigenetic events.

Another aim of our study was to assess the impact of these methylation changes on gene expression. Importantly, although we selected 39 single CpGs, our validation process revealed that hypermethylation in HCC occurs not only in the selected CpG, but also in the surrounding CpGs within the amplified PCR fragments suggesting a role in gene expression regulation. In this regard, it is widely accepted that the level of methylation in the regions known as CGI regulates gene expression, and that the location of these CGIs determines the direction of this regulation [6, 43, 45, 55]. As expected, and in agreement with the literature [6, 43, 45, 55], we found hypermethylated CpGs located within promoter CGIs that correlated with downregulation of gene expression and hypermethylated CpGs located in gene body CGIs associated with gene transcription activation. However, the changes in gene expression were really small. Moreover, and unexpectedly, among the 32 hypermethylated genes, we found ten that were not expressed in either liver or HCC, and five that despite being hypermethylated did not show any change in expression in HCC. In fact, it has been described that aberrant DNA methylation in cancer occurs in genes normally repressed in the corresponding normal tissues [48, 49], and importantly, that genes repressed in a specific tissue are prone to hypermethylation in cancers derived from that tissue [48, 49]. Our results are in line with these notions, as we found that the genes hypermethylated in several tumors were either not expressed or minimally expressed in control livers and other healthy tissues. However, the expression of the six genes (*EFNB2*, *MTHFD2*, *TSC22D1*, *CELF6*, *SH3YL1*, and *RNF135*) which exhibited DNA hypermethylation exclusively in HCC and not in other tumor types, was markedly low in liver tissue compared to other extrahepatic healthy tissues, suggesting a specific transcriptional repression in the liver.

All these findings raise intriguing questions about the role of DNA methylation in gene expression regulation and its implication in cancer development. They support the hypothesis that hypermethylation may be a “passenger” event, reflecting epigenetic dysregulation in cancer rather than contributing to its development [48, 49]. While it appears that hypermethylation does not have a significant impact on gene expression, particularly in genes that are already transcriptionally repressed, another question arises as to whether this methylation occurs randomly across CpG sites. However, in line with previous studies [14, 26] our results support that hypermethylation across multiple tumor types occurs predominantly in genes that are specifically repressed in the tissue of origin, suggesting a non-random pattern of methylation and a possible role in the epigenetic regulation of cancer. For instance, we cannot exclude that these marks remain as passengers reflecting other epigenetic changes in the tumor. Moreover, those marks could also impact the expression of distant genes through the modulation of chromatin architecture or the recruitment of chromatin and gene expression modulators. Finally, we can speculate a mechanistic role in transformation, and this hypermethylation could be required to ensure the repression of normally not expressed genes, which might otherwise interfere with the malignant transformation process if activated. Irrespective of their function, tumor hypermethylation marks may provide insights into their tissue of origin, and therefore have implications for the development of tissue-specific biomarkers, liquid biopsy approaches and targeted therapies.

In summary, further to the identification of CpGs specifically hypermethylated in HCC our study provides new data concerning the complex interplay between DNA methylation, gene expression, and tumorigenesis. This work underscores the need for further studies aimed at elucidating the underlying mechanisms and functional consequences of cancer-wide and tumor type-specific epigenetic alterations in cancer.

It categorizes these findings based on gene expression status (not expressed, not differentially expressed, downregulated, upregulated) and the location within CpG islands (CGIs).

## Supplementary Information

Below is the link to the electronic supplementary material.

Supplementary file1 (PDF 790 KB)

## Data Availability

No datasets were generated or analysed during the current study.

## Abbreviations

BRCA: Breast carcinoma
CESC: Cervical squamous cell carcinoma and endocervical adenocarcinoma
CGI: CpG Island
COADREAD: Colorectal adenocarcinoma
FDR: False discovery rate
GBM: Glioblastoma multiforme
GTEx: Genotype-Tissue Expression
HCC: Hepatocellular carcinoma
HM450: HumanMethylation450 BeadChip array
KIRC: Kidney renal clear cell carcinoma
LIHC: Liver hepatocellular carcinoma
LAML: Acute myeloid leukemia
LUNG: Lung adenocarcinoma
LUSC: Lung squamous cell carcinoma
NAFLD: Non-alcoholic fatty liver disease
NASH: Non-alcoholic steatohepatitis
OV: Ovarian serous cystadenocarcinoma
PCR: Polymerase Chain Reaction
SARC: Sarcoma
SKCM: Skin cutaneous melanoma
STAD: Stomach adenocarcinoma
TCGA: The Cancer Genome Atlas
THCA: Thyroid carcinoma
THYM: Thymoma
TGCT: Testicular germ cell tumors
UCS: Uterine carcinosarcoma
UMAP: Uniform manifold approximation and projection
