# Supplementary material for: Comprehensive in silico CpG methylation analysis in hepatocellular carcinoma identifies tissue- and tumor-type specific marks disconnected from gene expression
Source: J Physiol Biochem. 2024 Sep 21;80(4):865–79. doi: 10.1007/s13105-024-01045-8 (PMC11682006; doi:10.1007/s13105-024-01045-8)
Supplement: Supplementary file 1 — Supplementary file1 (PDF 790 KB) [file 13105_2024_1045_MOESM1_ESM.pdf]

Supplementary Information

Comprehensive *in silico* CpG methylation analysis in hepatocellular carcinoma identifies tissue- and tumor-type specific marks disconnected from gene expression

Idoia Bilbao[1], Miriam Recalde[2], Fabrice Daian[3], José Maria Herranz[2,4], María Elizalde[2], Mercedes Iñarrairaegui[1,4,5], Matteo Canale[6], Maite G Fernández-Barrena[2,4,5], Andrea Casadei-Gardini[7,8], Bruno Sangro[1,4,5], Matías A Ávila[2,4,5], Manuel F Landecho Acha[9], Carmen Berasain[2,4,#], María Arechederra[2,4,5,#].

- **Supplementary Figure Legends**
- **Supplementary Table Legends**
- **Supplementary Figures**
- **Supplementary Tables**

SUPPLEMENTARY FIGURE LEGENDS.

Supplementary Figure S1. Global analyses of the three HCC tissue methylomes used for the identification of the hypermethylated CpG list. (A) Schematic representation of the HCC tissue methylomes used for the *in silico* identification of hypermethylated CpGs in HCC. **(B)** Hierarchical clustering of the tumor and non-tumoral subgroups of the three studies indicated in Figure S1A performed using the overall DNA methylation content. **(C)** Dimensionality reduction by UMAP represented as a scatterplot using the different non-tumoral and HCC tissues of the three studies indicated in Figure S1A. **(D)** CGI mean methylation (β-values) in control samples (n=135) compared to HCC samples (n=670). T-test p-value = 4.31E-28.

Supplementary Figure S2. Analyses of the methylation level of the identified hypermethylated CpGs in different liver diseases. (A) Heatmap reporting the mean methylation levels of each of the 39 CpGs hypermethylated in HCC in the three datasets used to analyze methylation status in different non-tumor liver disease GSE61258, GSE60753 and GSE48325. The CpGs are ranked as in Figure 1B. The color scale bar is shown on the right. (NAFLD: non alcoholic fatty liver disease; NASH: nonalcoholic steatohepatitis; PBC: primary biliary cirrhosis; PSC: primary sclerosing cholangitis). **(B)** Graph showing for each of the 39 selected CpGs, the percentage of patients with the CpG methylated (β-value >0.2). The 39 CpGs are ranked from the CpG methylated in the highest (84.95%) percentage of patients to the lowest (54.70%). **(C)** Heatmap reporting the mean methylation levels of each of the 39 CpGs hypermethylated in HCC in primary hepatocytes (n=17) and HCC cell lines (FLNEO, H801, HCO2, Hep3B, Huh75, LH86, SNU423, SNU449) from GSE60753. The color scale bar is shown on the left.

Supplementary Figure S3. Chromosomal distribution of the 39 identified CpGs. Graph showing the chromosomal distribution of the 39 selected CpGs.

SUPPLEMENTARY TABLE LEGENDS.

Supplementary Table S1. Table that contains information from the 3862 CpGs hypermethylated (with a median methylation difference >0.2 and an FDR <0.05) in HCC compared to non-tumoral tissues from GSE56588 (224 HCC, 9 cirrhotic livers and 10 control livers), GSE54503 (66 HCC and 66 peritumoral liver samples), and TCGA-LIHC (380 HCC and 50 peritumoral liver samples). CpG site (hg19) in the Illumina 450K array, CGI (hg19) and gene name is reported.

Supplementary Table S2. Table that contains information from the 1061 CpGs methylated in HCC tissues but not in any of the blood samples analyzed. CpG site (hg19) in the Illumina 450K array, CGI (hg19) and gene name is reported.

Supplementary Table S3. Table that contains information from the 39 CpGs selected as only hypermethylated (β-value >0.3) in HCC and not methylated (β-value <0.2) in either control liver, cirrhotic tissue or whole blood. CpG ID, CpG site (hg19) in the Illumina 450K array, CpG site (hg19), CGI (hg19), gene name, genomic region is reported.

Supplementary Table S4. Table that contains information from the 28 methylomes available in the TCGA. Study acronym, full name, number of non-tumor and tumor samples.

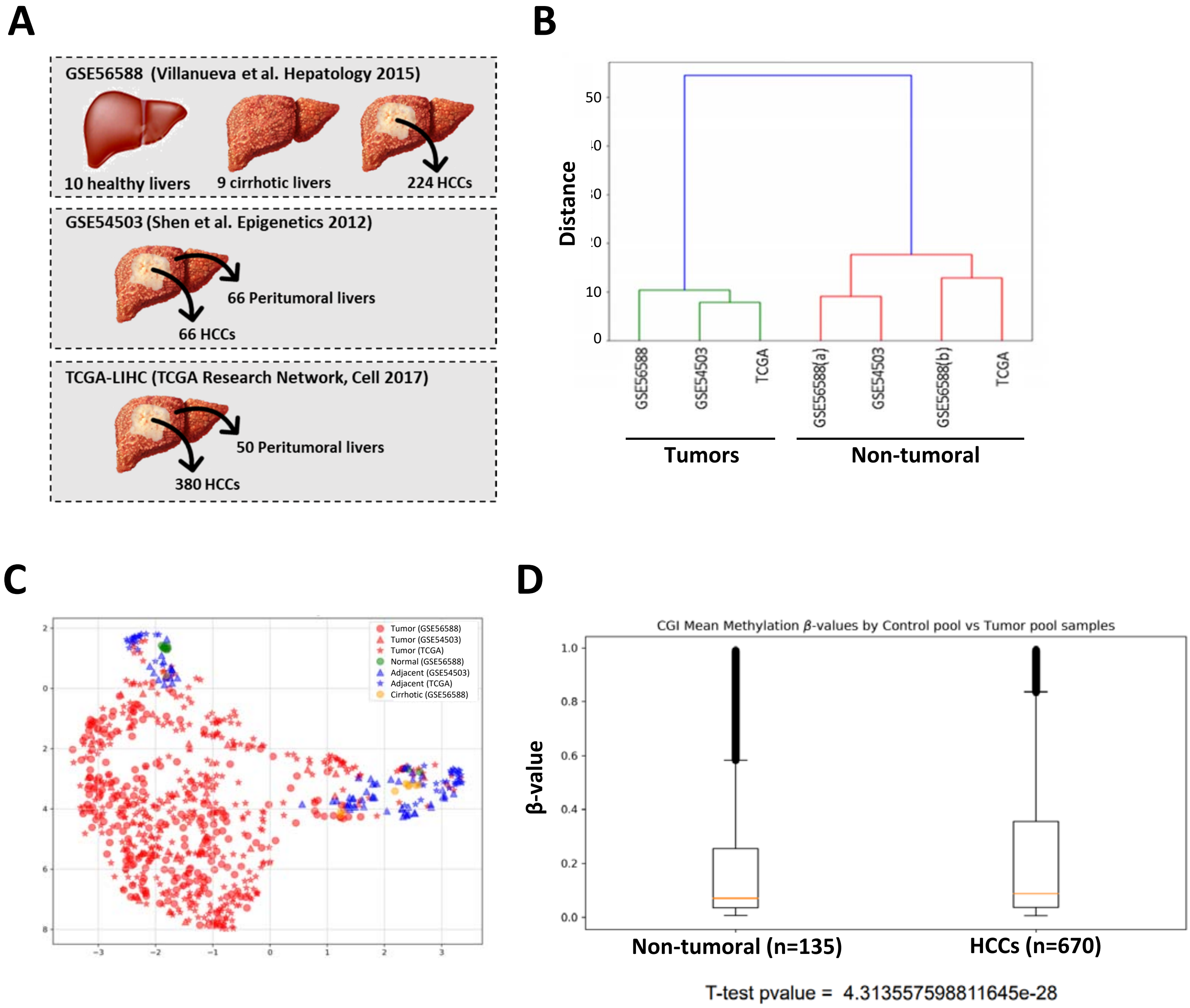

Supplementary Figure S1

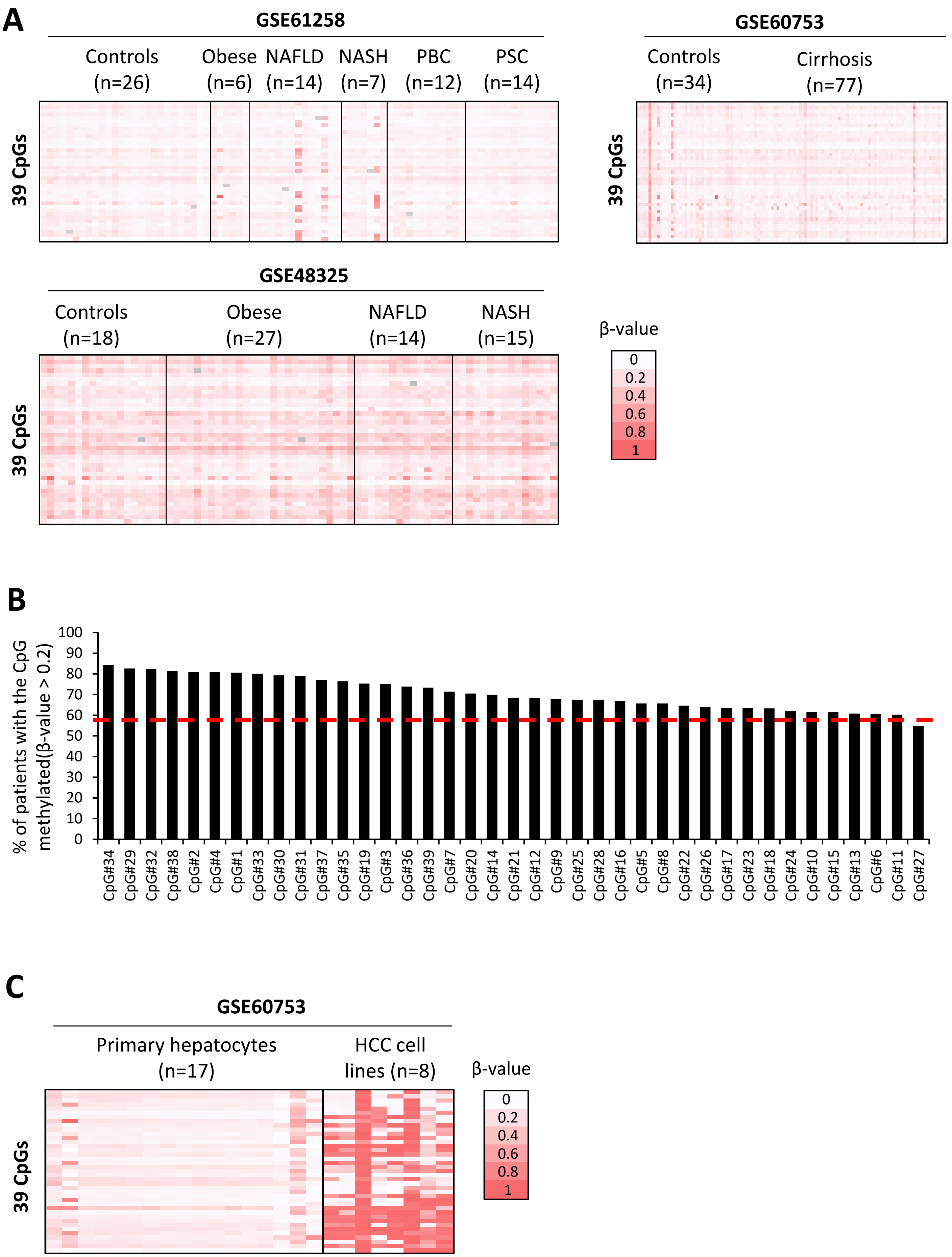

Supplementary Figure S2

A

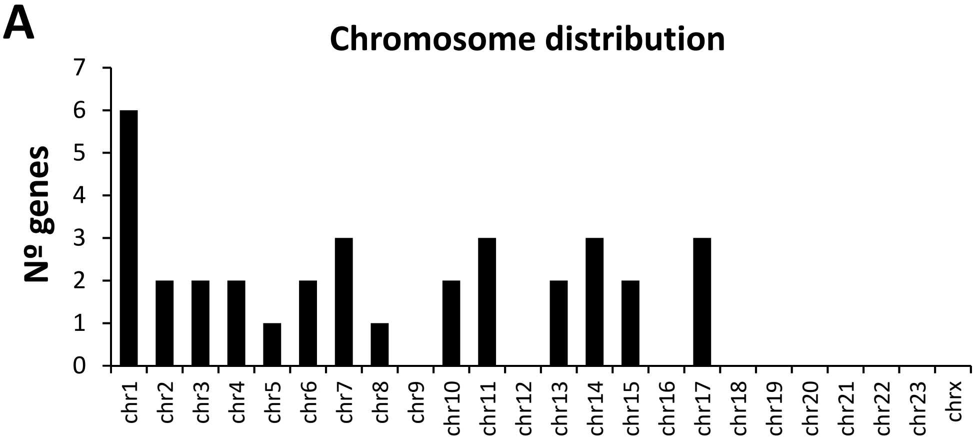

Supplementary Figure S3

Table S1

CpG site (hg19)_Illumina 450K Array	CGI (hg19)	Gene name
cg00002719	chr1:169396622-169396869	CCDC181
cg00022911	chr19:19371676-19372393	HAPLN4
cg00029353	chr12:52240773-52242547	
cg00030422	chr3:38080629-38081187	DLEC1
cg00032205	chr8:98289605-98290404	TSPYL5
cg00035636	chr13:21900426-21900637	MIPEPP3
cg00037457	chr17:59531724-59535254	TBX4
cg00043095	chr2:223288923-223290013	SGPP2
cg00044796	chr1:146552329-146552577	
cg00056074	chr2:131485150-131485460	
cg00058329	chr12:133194869-133195544	P2RX2
cg00090261	chr8:101117923-101118693	RGS22
cg00094518	chr7:130417913-130419378	KLF14
cg00097146	chr2:45169506-45171884	SIX3
cg00108164	chr2:263401-265238	SH3YL1
cg00114029	chr1:35350879-35351854	DLGAP3
cg00117172	chr1:25255528-25259005	RUNX3
cg00121634	chr3:169529876-169530435	LRRC34
cg00121843	chr13:33923931-33924781	STARD13
cg00133595	chr16:31225957-31228264	TRIM72
cg00152008	chr1:50884229-50891471	
cg00155526	chr5:32709911-32714450	NPR3
cg00155882	chr8:141107838-141110984	TRAPPC9
cg00157796	chr6:159589637-159591319	
cg00158789	chr17:59531724-59535254	TBX4
cg00164949	chr17:43037167-43037740	C1QL1
cg00166216	chr3:194406441-194409045	FAM43A
cg00169412	chr6:27463177-27463379	
cg00172597	chr1:223536843-223538628	
cg00177388	chr5:72676121-72678421	
cg00187933	chr4:995483-997541	IDUA
cg00221382	chr16:54317822-54324604	
cg00249511	chr11:626729-628037	SCT
cg00278547	chr2:177036255-177037213	HOXD3
cg00292664	chr10:71077947-71079377	HK1
cg00295794	chr13:100641335-100642188	
cg00298065	chr19:58715221-58716033	ZNF274
cg00303982	chr8:145555343-145562310	SCRT1
cg00307214	chr14:69726251-69728393	GALNT16
cg00314240	chr1:92495368-92495964	EPHX4
cg00318796	chr3:68056900-68057167	FAM19A1
cg00332937	chr5:72740052-72740800	
cg00338735	chr4:53728038-53729000	RASL11B
cg00346208	chr1:20669567-20670018	VWA5B1
cg00356183	chr7:751713-752150	PRKAR1B
cg00366850	chr2:45231782-45232550	SIX2

cg00370229	chr6:29521111-29521833	
cg00384539	chr8:70981874-70984888	PRDM1 4
cg00388812	chr8:99960498-99961438	OSR2
cg00393798	chr11:8615398-8615889	STK33
cg00393825	chr20:42135794-42137062	L3MBTL1
cg00396667	chr5:134363093-134365146	PITX1
cg00399175	chr2:26395417-26395620	
cg00421139	chr8:97172635-97173880	GDF6
cg00421363	chr19:49646093-49646308	PPFIA3
cg00437985	chr16:21294981-21295414	CRYM
cg00450824	chr11:8615398-8615889	STK33
cg00456086	chr3:197281606-197283128	BDH1
cg00458878	chr7:27260102-27260467	
cg00460049	chr1:66998729-66999060	SGIP1
cg00478479	chr19:40904445-40904955	PRX
cg00489401	chr5:180075689-180076906	FLT4
cg00495860	chr21:38065180-38066185	
cg00497232	chr4:6665672-6666089	
cg00500564	chr10:128993506-128995167	FAM196A
cg00527440	chr2:242549374-242549995	THAP4
cg00529958	chr13:100620242-100624348	ZIC5
cg00544436	chr6:34202568-34206193	HMGA1
cg00544449	chr15:79724100-79725643	KIAA1024
cg00553149	chr7:99774734-99775583	STAG3
cg00554413	chr1:59042014-59043295	TACSTD2
cg00556112	chr11:636907-640628	DRD4
cg00560542	chr10:75407414-75407706	SYNPO2L
cg00573330	chr4:330163-332068	ZNF141
cg00582971	chr5:178421226-178422337	GRM6
cg00584713	chr1:213123648-213125092	VASH2
cg00599393	chr8:22457124-22457753	C8orf58
cg00612595	chr21:47717236-47718059	YBEY
cg00619126	chr2:105459128-105461770	LOC100506421
cg00626110	chr10:134597358-134602649	NKX6-2
cg00637687	chr5:95768875-95769080	PCSK1
cg00642460	chr5:176827082-176827754	PFN3
cg00648301	chr20:20344401-20350605	INSM1
cg00650809	chr19:17438055-17440318	ANO8
cg00667751	chr6:28956107-28956578	
cg00667789	chr1:59042014-59043295	TACSTD2
cg00674365	chr19:57018744-57019506	ZNF471
cg00674679	chr2:176931576-176932663	
cg00674706	chr6:29521111-29521833	
cg00688297	chr8:145751956-145756326	C8orf82;LCRR24
cg00689492	chr4:1303491-1303835	MAEA
cg00699945	chr10:134597358-134602649	NKX6-2
cg00714725	chr10:94825547-94826320	CYP26C1
cg00723271	chr14:102247661-102248279	PPP2R5C
cg00723994	chr12:115104849-115105548	
cg00727630	chr20:50721311-50721912	ZFP64

cg00731785	chr4:15704641-15705000	BST1
cg00741609	chr10:102893661-102895059	TLX1
cg00741624	chr14:93896737-93897466	UNC79
cg00748373	chr16:68676451-68676843	
cg00752195	chr4:1396292-1401730	
cg00753478	chr12:21810489-21810766	LDHB
cg00755043	chr9:91149946-91150648	NXNL2
cg00767010	chr9:99481903-99482470	
cg00772020	chr7:100806280-100809064	VGF
cg00773413	chr10:77155129-77169600	ZNF503-AS2
cg00773902	chr19:19006032-19007546	CERS1
cg00788177	chr6:159360626-159360952	
cg00800512	chr19:58094740-58095764	ZIK1
cg00800993	chr20:58514072-58515636	PPP1R3D; FAM21 7B
cg00803088	chr10:43600147-43601165	RET
cg00806198	chr17:72968168-72969164	HID1
cg00807871	chr6:37616723-37617179	MDGA1
cg00817367	chr12:52400468-52401696	GRASP
cg00824018	chr10:105036629-105038084	INA
cg00849610	chr17:59528980-59530266	TBX4
cg00864183	chr17:48045789-48047207	DLX4
cg00865356	chr4:91048412-91049682	CCSER1
cg00866399	chr2:73143056-73148260	EMX1
cg00871706	chr7:138666359-138666972	
cg00875989	chr18:44772993-44775577	SKOR2
cg00884093	chr22:46929318-46934861	CELSR1
cg00890257	chr14:100625320-100626461	DEGS2
cg00891278	chr3:126113548-126113967	CCDC37
cg00894757	chr3:170136243-170137886	CLDN11
cg00896540	chr8:82192379-82193685	FABP5
cg00907283	chr22:20286319-20287630	
cg00909646	chr8:67873389-67875600	TCF24
cg00914041	chr8:91997231-91997906	LOC100127983
cg00922376	chr1:14026482-14027200	PRDM2
cg00926400	chr2:198649752-198651599	BOLL
cg00939495	chr4:9783036-9784960	DRD5
cg00956199	chr10:8077830-8078378	
cg00958884	chr17:74533282-74534566	PRCD; CYGB
cg00959249	chr5:95768875-95769080	PCSK1
cg00963169	chr1:50513645-50514320	ELAVL4
cg00966099	chr4:184718264-184719754	
cg00971050	chr17:40573741-40575526	PTRF
cg00981879	chrX:39680141-39681418	
cg00983948	chr6:158937286-158937491	
cg00987080	chr1:32930501-32931061	ZBTB8B
cg00995327	chr3:142837886-142840838	CHST2
cg01003961	chr7:20823308-20825662	SP8
cg01009697	chr9:87283179-87285704	NTRK2
cg01025774	chr5:42944267-42944582	
cg01031101	chr6:28226980-28227483	NKAPL; ZKSCAN4

cg01047586	chr2:176933132-176933449	
cg01048445	chr2:241771656-241772194	
cg01060026	chr3:129323916-129325332	PLXND1
cg01072076	chr5:1930755-1931986	AC124852
cg01076997	chr22:37815804-37816477	ELFN2
cg01097384	chr11:1357627-1359081	
cg01108118	chr6:391189-393790	IRF4
cg01112965	chr19:38852312-38853485	CATSPERG
cg01120173	chr17:5019638-5019879	ZNF232
cg01135780	chr5:158527375-158527983	AK123543
cg01142668	chr16:2086421-2086892	SLC9A3R2
cg01143804	chr4:40751843-40752493	NSUN7
cg01163842	chr14:95234613-95236569	GSC
cg01172903	chr19:12203029-12203503	ZNF788
cg01175020	chr10:102896343-102896665	TLX1
cg01178624	chr11:65359293-65360328	KCNK7
cg01178680	chr15:74425092-74428821	ISLR2
cg01181227	chr4:1396292-1401730	
cg01196531	chr15:53080459-53083699	ONECUT1
cg01204305	chr3:49755764-49757317	AMIGO3; GMPPB; RNF123
cg01204606	chr11:116450660-116451912	
cg01215762	chr7:27212417-27214396	HOXA10
cg01237333	chr9:112917-114839	
cg01240444	chr12:124246525-124247254	DNAH10
cg01246835	chr7:6703504-6704075	AK123300
cg01268683	chr19:53141176-53141813	ZNF83
cg01281911	chr1:47881897-47883065	FOXE3
cg01288372	chr2:209271338-209271987	PTH2R
cg01294058	chr11:2905024-2907024	CDKN1C
cg01295392	chr12:108168987-108169570	ASCL4
cg01303723	chr10:134597358-134602649	NKX6-2
cg01312482	chr5:178450631-178451216	ZNF879
cg01321673	chr22:50438258-50438776	IL17REL
cg01323148	chr2:206549600-206551316	NRP2
cg01348293	chr7:27260102-27260467	
cg01357429	chr7:27284640-27286237	EVX1
cg01370541	chr4:41882451-41882964	BC025350
cg01373292	chr1:7764594-7765856	CAMTA1
cg01379240	chr3:138663719-138666346	FOXL2; FOXL2NB
cg01381846	chr7:27203916-27206462	HOXA9
cg01384488	chr10:134597358-134602649	NKX6-2
cg01419831	chr2:162283582-162284677	
cg01420426	chr1:41831977-41832542	
cg01422476	chr16:1921898-1922426	MEIOB
cg01423964	chr1:111216245-111217937	KCNA3
cg01428589	chr19:33165267-33168099	RGS9BP
cg01435344	chr10:99609426-99610116	GOLGA7B
cg01436998	chr19:38307879-38308526	LOC644554
cg01438365	chr17:59473061-59483266	TBX2
cg01439876	chr1:119543057-119543454	

cg01445942	chr7:72847934-72850032	FZD9
cg01447112	chr7:6703504-6704075	AKT123300
cg01449136	chr14:61108955-61109786	
cg01452847	chr17:46710813-46711419	
cg01480180	chr7:90893568-90896683	FZD1
cg01482645	chr8:104510871-104513913	RIMS2
cg01485998	chr1:213123648-213125092	VASH2
cg01490894	chr6:26550783-26551114	
cg01499217	chr18:10454454-10455268	APCDD1
cg01503065	chr4:155410624-155413393	DCHS2
cg01513307	chr6:108478524-108479558	AF520419
cg01527459	chr10:82116203-82117120	DYDC2
cg01529637	chr20:50721311-50721912	ZFP64
cg01532168	chr8:140714586-140718259	KCNK9
cg01532436	chr19:48983563-48984175	CYTH2
cg01535733	chr6:153450993-153452792	RGS17
cg01538344	chrX:100546064-100546550	TAF7L
cg01541645	chr14:61103979-61104663	
cg01543654	chr17:46800534-46800746	HOSXB-AS5
cg01550055	chr1:119543057-119543454	
cg01556502	chr14:103655242-103655928	LINC00605
cg01557547	chr3:157822974-157823836	SHOX2
cg01558212	chr2:200320503-200329681	SATB2
cg01561869	chr5:140725052-140725891	PCDHGA1
cg01565320	chr4:42399153-42400802	SHISA3
cg01571001	chr3:128215213-128216905	
cg01576677	chr2:45029729-45030035	
cg01579950	chr11:64480200-64481344	NRXN2
cg01592801	chr8:99438693-99440425	KCNS2
cg01597066	chr19:49127389-49127720	SPHK2
cg01603290	chr17:40805675-40805957	
cg01610632	chr3:147126989-147128999	ZIC1
cg01627847	chr17:43339125-43339832	SPATA32; MAP3K14-AS1
cg01629716	chr15:45996454-45996735	
cg01635405	chr10:124894076-124897366	HMX3
cg01642521	chr11:20618198-20619920	SLC6A5
cg01645753	chr15:83315117-83317541	CPEB1
cg01649597	chr2:232394764-232395337	NMUR1
cg01658421	chr2:176944088-176948446	EVX2
cg01684881	chr17:42634830-42636851	FZD2
cg01692233	chr18:76737006-76741244	SALL3
cg01696193	chr6:56818874-56820308	BEND6
cg01718447	chr7:30721373-30722445	CRHR2
cg01720033	chr17:62774367-62777797	BPTFP1-KPNA2P3; ARHGAP2
cg01723148	chr12:58021295-58022037	B4GALNT1
cg01725199	chr9:101569906-101570349	GALNT12
cg01730970	chr1:27687025-27687743	MAP3K6
cg01734112	chr2:26624604-26625057	DRC1
cg01759761	chr5:7849946-7850439	C5orf49
cg01762827	chr16:69139629-69141900	HAS3

cg01765249	chr17:59473061-59483266	TBX2
cg01777643	chr1:224803638-224805314	CNIH3
cg01781974	chr14:52534582-52536722	NID2
cg01791874	chr5:16179065-16180420	MARCH11
cg01806520	chr8:93114057-93115979	RUNX1T1
cg01815671	chr3:126113548-126113967	CCDC37
cg01817364	chr5:43037260-43037520	
cg01824969	chr8:67873389-67875600	TCF24
cg01833196	chr10:105452339-105453230	SH3PXD2A
cg01851088	chr3:48698336-48701667	CELSR3
cg01860753	chr1:206730398-206730908	RASSF5
cg01870826	chr7:5111621-5112088	RBAKDN
cg01881524	chr5:54179364-54180171	
cg01885071	chr14:90167783-90168352	
cg01893212	chr7:49813009-49815752	VWC2
cg01901107	chr7:150747977-150748519	ASIC3
cg01912921	chr6:1604607-1615866	FOXC1
cg01913455	chr17:59473061-59483266	TBX2
cg01921432	chr1:91182510-91182857	BARHL2
cg01923218	chr11:93063599-93064076	CCDC67
cg01927745	chr5:72676121-72678421	
cg01939477	chr11:43602546-43603215	MIR129-2
cg01959562	chr4:57975861-57976916	IGFBP7; IGFBP7-AS1
cg01961447	chr22:38220590-38221619	GALR3
cg01964152	chr6:30095174-30095610	
cg01965047	chr16:2139804-2142170	PKD1
cg01972418	chr14:37125220-37125661	
cg01981601	chr8:60032533-60032746	
cg01992935	chrX:108867763-108868486	KCNE1L
cg01992990	chr1:202779791-202780684	KDM5B-AS1
cg01995480	chr22:45403037-45406372	PHF21B
cg01996714	chr20:3218579-3220930	SLC4A11
cg02009088	chr5:139227606-139228279	NRG2
cg02011074	chr1:171810468-171811325	DNM3
cg02017200	chr19:49255779-49256495	FUT1
cg02018277	chr15:65116014-65116567	PIF1
cg02037307	chr5:134363093-134365146	PITX1
cg02038168	chr22:39784355-39785104	TAB1
cg02052142	chr14:60794577-60794867	
cg02062480	chr6:108485672-108490539	NR2E1
cg02063759	chr1:119543057-119543454	
cg02063817	chr7:27252682-27253835	
cg02065637	chr20:61806255-61810867	
cg02071005	chr2:175199464-175202639	SP9
cg02072400	chr12:127630512-127631199	
cg02081266	chr17:59528980-59530266	TBX4
cg02090762	chr17:79503587-79504181	FSCN2
cg02093732	chr15:30515267-30518445	DLKZP434L187
cg02096396	chr2:26395417-26395620	GAREML
cg02109484	chr2:74875129-74875562	M1AP

cg02119792	chr21:47009997-47011163	
cg02119927	chr10:71077947-71079377	HK1
cg02132470	chr7:98467024-98468058	TMEM130
cg02143877	chr20:52789253-52790986	CYP24A1
cg02147764	chr2:45169506-45171884	SIX3
cg02149189	chr6:29521111-29521833	UBD
cg02164046	chr3:187387915-187388176	SST
cg02164129	chr5:76925446-76926875	OTP
cg02168303	chr7:27265159-27265493	
cg02177231	chr1:119529820-119530712	TBX15
cg02215070	chr7:134143116-134144063	AKR1B1
cg02227496	chr9:33750520-33751160	PRSS3
cg02227867	chr8:22457124-22457753	C8orf58
cg02246609	chr6:28583935-28584289	ZBED9
cg02253760	chr3:147077116-147077672	
cg02262187	chr16:31498651-31500564	SLC5A2
cg02266771	chr6:27218793-27219813	PRSS16
cg02267270	chr6:37616394-37616621	MDGA1
cg02280532	chr2:160760605-160761452	LY75
cg02288341	chr5:137610106-137610311	GFRA3
cg02293118	chr7:44349112-44349716	CAMK2B
cg02295078	chr17:16472075-16472800	ZNF287
cg02306630	chr10:134597358-134602649	NKX6-2
cg02320543	chr14:61108955-61109786	
cg02320862	chr17:75368689-75370506	SEPTIN9
cg02325540	chr10:94833273-94835256	CYP26A1
cg02330121	chr6:118228043-118229811	SLC35F1
cg02337836	chr1:110210582-110210956	GSTM2
cg02339682	chr6:56818874-56820308	DST
cg02346492	chr17:9142981-9143839	NTN1
cg02349096	chr19:11450017-11450414	RAB3D
cg02362385	chr19:19738573-19739821	LPAR2
cg02373152	chr6:52226754-52228006	PAQR8
cg02380334	chr14:70038109-70040302	CCDC177
cg02392619	chr1:213123648-213125092	VASH2
cg02396496	chr17:74533282-74534566	PRCD; CYGB
cg02399524	chr11:63258379-63258804	HRASLS5
cg02401454	chr16:230302-231761	HBQ1
cg02408775	chr12:48206722-48207126	HDAC7
cg02409878	chr8:99960498-99961438	OSR2
cg02428119	chr19:58520755-58521224	
cg02430935	chr10:124907284-124911035	HMX2
cg02433564	chr6:37673309-37673774	
cg02434443	chr11:15136059-15136545	INSC
cg02446647	chr6:133562087-133563586	EYA4
cg02447304	chr6:74024403-74025020	
cg02452966	chr19:39055500-39056379	RYR1
cg02457680	chr1:154474108-154475699	TDRD10
cg02466113	chr4:76555367-76556079	CDKL2
cg02467990	chr7:49813009-49815752	VWC2

cg02473540	chr19:58570394-58571779	ZNF135
cg02478448	chr13:37005582-37006453	CCNA1
cg02487331	chr1:146550291-146551260	
cg02511231	chr5:170735170-170739863	TLX3
cg02523270	chr3:193987426-193987758	
cg02527669	chr2:220416864-220417869	OBSL1
cg02532096	chr11:111410933-111412199	LAYN
cg02533423	chr7:27231806-27233097	
cg02553663	chr17:80291021-80292394	SECTM1
cg02557695	chr7:90893568-90896683	FZD1
cg02567119	chr10:102891011-102891794	TLX1NB
cg02571816	chr19:38746639-38747379	PPP1R14A
cg02578368	chr6:26250437-26250827	HIST1H3F
cg02583418	chr2:105468852-105473488	POU3F3
cg02583525	chr2:26624604-26625057	DRC1
cg02586551	chr11:73693703-73694802	UCP2
cg02587316	chr19:37095681-37096589	ZNF382
cg02590401	chr19:48983563-48984175	CYTH2
cg02592271	chr2:27665252-27665670	KRTCAP3
cg02598441	chr17:62774367-62777797	BPTFP1-KPNA2P3; ARHGAP2
cg02604524	chr3:128719866-128721245	EFCC1
cg02622316	chr6:28367125-28367651	ZSCAN12
cg02623400	chr1:50513645-50514320	ELAVL4
cg02624770	chr7:28995306-28998541	TRIL
cg02629281	chr11:8102359-8102913	TUB
cg02631838	chr1:33358470-33359449	HPCA
cg02636041	chr10:43697778-43698177	RASGEF1A
cg02637078	chr19:55684877-55685317	SYT5
cg02637318	chr8:38965014-38965423	ADAM32
cg02647941	chr3:147126989-147128999	ZIC1
cg02659086	chr11:67350929-67351953	GSTP1
cg02659794	chr12:21810489-21810766	LDHB
cg02668581	chr2:216877900-216878352	MREG
cg02675308	chr4:76555367-76556079	CDKL2
cg02683759	chr2:157176252-157177346	
cg02685896	chr17:27038085-27038919	PROCA1
cg02694017	chr19:54485304-54486322	CACNG8
cg02694427	chr2:176964063-176965509	HOXD2
cg02699694	chr2:9143128-9144630	MBOAT2
cg02710296	chr1:182921820-182922644	SHCBP1L
cg02712145	chr7:30721373-30722445	CRHR2
cg02716516	chr3:45837481-45838256	SLC6A20
cg02728595	chr3:196255496-196256013	
cg02732252	chr16:3067522-3068358	CLDN6
cg02741521	chr3:9745500-9746782	CPNE9
cg02745211	chr3:132756986-132757895	TMEM108
cg02745636	chr14:61103979-61104663	
cg02746869	chr10:17270431-17272617	VIM
cg02747748	chr17:43506829-43510380	ARHGAP27
cg02759846	chr19:51227662-51228883	CLEC11A

cg02770054	chr19:36048557-36049673	ATP4A
cg02776659	chr21:47717236-47718059	YBEY
cg02776664	chr19:12978360-12978785	MAST1
cg02781618	chr4:42152767-42154984	BEND4
cg02782426	chr3:40428652-40429015	ENTPD3
cg02787991	chr17:80291021-80292394	SECTM1
cg02788401	chr17:48585386-48586167	MYCBPAP
cg02792538	chr22:38476837-38478839	SLC16A8
cg02798576	chr10:101290026-101290338	LINC01475
cg02801359	chr20:58507756-58509517	FAM217B
cg02811627	chr14:103394398-103397070	AMN
cg02823783	chr12:124246525-124247254	DNAH10
cg02826439	chr3:120003884-120004426	GPR156
cg02829688	chr1:119526783-119527192	TBX15
cg02831587	chr11:13689589-13690724	FAR1
cg02849693	chr19:54401705-54402472	PRKCG
cg02858118	chr3:48693119-48694768	CELSR3
cg02863489	chr16:2765620-2765855	PRSS27
cg02863856	chr13:33923931-33924781	STARD13
cg02874376	chr14:101192852-101193499	DLK1
cg02881929	chr4:41882451-41882964	LINC00682
cg02884176	chr2:88751336-88752865	FOXI3
cg02884239	chr17:75368689-75370506	SEPTIN9
cg02886408	chr10:133794899-133796598	BNIP3
cg02893180	chr22:50919454-50920376	ADM2
cg02900766	chr15:58357310-58358776	ALDH1A2
cg02909790	chr6:26271346-26271588	HIST1H3G
cg02912326	chr6:97284831-97285782	GPR63
cg02919936	chr8:70981874-70984888	PRDM14
cg02928664	chr19:54974176-54976819	CDC42EP5
cg02935627	chr19:290595-292249	PLPP2
cg02938303	chr3:45187027-45187946	CDCP1
cg02952008	chr1:228603707-228604567	TRIM17
cg02969243	chrX:11445528-11446182	ARHGAP6
cg02970297	chr7:102920310-102921514	DPY19L2P2
cg02970696	chr3:125075916-125076481	ZNF148
cg02973171	chr11:82443436-82444976	FAM181B
cg03005603	chr19:52873085-52873572	ZNF880
cg03011535	chr20:42543098-42545137	TOX2
cg03018796	chr22:37730552-37731415	
cg03023152	chr8:102505513-102506430	GRHL2
cg03024619	chr3:71802561-71804209	EIF4E3
cg03045635	chr4:9783036-9784960	DRD5
cg03048083	chr17:43339125-43339832	SPATA32; MAP3K14-AS1
cg03058660	chr19:48919174-48919412	GRIN2D
cg03059131	chr15:60296136-60298520	FOXB1
cg03062252	chr6:37616394-37616621	MDGA1
cg03063639	chr15:83775862-83776922	TM6SF1
cg03066050	chr9:110249749-110252660	KLF4
cg03070194	chr1:110210582-110210956	GSTM2

cg03070297	chr10:128993506-128995167	DOCK1
cg03073402	chr19:42927286-42928598	LIPE
cg03109306	chr11:123524734-123525685	SCN3B
cg03109827	chr10:133109194-133111052	TCERG1L
cg03113878	chr7:100091181-100091598	NYAP1
cg03127334	chr21:40032244-40033665	ERG
cg03129384	chr10:128993506-128995167	DOCK1
cg03141620	chr2:172949243-172950126	
cg03157698	chr10:113943284-113943657	GPAM
cg03166753	chr6:38682950-38683265	DNAH8
cg03166835	chr19:50393070-50393998	IL4I1
cg03172947	chr20:44034459-44035954	DBNDD2
cg03190219	chr7:25898238-25898771	
cg03192598	chr19:12305553-12306304	
cg03200052	chr1:223536843-223538628	SUSD4
cg03204605	chr4:7940564-7941853	AFAP1
cg03208016	chr1:180123556-180124584	QSOX1
cg03209642	chr15:91427541-91427905	FES
cg03216846	chr1:119543822-119544339	
cg03217795	chr16:23846942-23848102	PRKCB
cg03228132	chr7:129780475-129781597	
cg03241244	chr12:122016171-122017693	KDM2B
cg03244411	chr6:99295742-99296454	
cg03255182	chr12:54423428-54423712	HOXC6
cg03257575	chr1:46951169-46951792	
cg03289872	chr19:56988314-56989741	ZNF667
cg03306374	chr16:23846942-23848102	PRKCB
cg03306486	chr19:1465207-1471241	APC2
cg03308628	chr12:95941907-95942979	USP44
cg03309253	chr19:41119032-41120394	LTBP4
cg03310242	chr12:2903390-2905189	FKBP4
cg03313945	chr13:100620242-100624348	ZIC5
cg03321592	chr1:19991147-19992788	HTR6
cg03323636	chr11:9025096-9026315	NRIP3
cg03323696	chr5:59189047-59189894	PDE4D
cg03326059	chr11:13689589-13690724	FAR1
cg03333634	chr8:11555100-11555603	GATA4
cg03336086	chr5:79330929-79331488	THBS4
cg03340649	chr3:44626326-44626794	ZNF660
cg03348397	chr8:54789582-54790665	RGS20
cg03348461	chr1:7022002-7022308	CAMTA1
cg03354992	chr10:88149267-88149738	
cg03361164	chr6:100915603-100915883	
cg03363161	chr3:147138917-147139564	LOC440982
cg03397307	chr12:3862069-3862606	CRACR2A
cg03405315	chr11:8615398-8615889	STK33
cg03407747	chr17:6898821-6900427	ALOX12-AS1
cg03409187	chr6:29521111-29521833	
cg03410231	chr7:143042509-143042867	CLCN1
cg03422911	chr1:237205127-237206644	RYR2

cg03423149	chr11:63766059-63768666	MACROD1
cg03444245	chr11:45376932-45377243	LOC101928812
cg03451670	chr2:236578081-236580153	AGAP1
cg03455458	chr1:213123648-213125092	VASH2
cg03467725	chr16:1921898-1922426	MEIOB
cg03468349	chr2:263401-265238	SH3YL1
cg03496114	chr17:80329067-80330208	
cg03499234	chr1:47909713-47911020	
cg03499808	chr11:73018453-73020563	ARHGEF17
cg03506033	chr11:75378417-75379882	MAP6
cg03509671	chr3:101497831-101498648	NXPE3
cg03517376	chr16:30389036-30390631	MYLPF
cg03524572	chr20:62283517-62284835	STMN3
cg03526459	chr1:146549851-146550075	
cg03536474	chr12:54366816-54369103	HOTAIR
cg03544320	chr4:5894072-5895116	CRMP1
cg03562414	chr19:52390842-52391368	ZNF577
cg03585419	chr19:54369388-54369809	MYADM
cg03593550	chr19:54485304-54486322	CACNG8
cg03599078	chr10:8077830-8078378	
cg03607359	chr5:176056521-176057494	SNCB
cg03609960	chr12:99288223-99289374	ANKS1B
cg03611452	chr19:38182794-38183327	ZNF781
cg03612722	chr22:27053082-27053629	MIAT
cg03614193	chr18:55019708-55021605	ST8SIA3
cg03640756	chr5:140864528-140864748	,8,9,10,11,12; PCDHGB1,2,3
cg03646234	chr3:46742765-46743086	TMIE
cg03650119	chr1:228345378-228346700	GJC2
cg03650674	chr8:102505513-102506430	GRHL2
cg03651493	chr5:32709911-32714450	NRP3
cg03656099	chr17:59473061-59483266	TBX2
cg03666741	chr11:9025096-9026315	NRIP3
cg03671191	chr19:52207133-52207731	SPACA6
cg03673965	chr6:108497596-108497996	NR2E1
cg03679755	chr10:101874822-101875139	TPM4P1
cg03695666	chr19:38210108-38210692	ZNF607
cg03698009	chr7:27203916-27206462	HOXA9
cg03699623	chr2:121200504-121200788	
cg03705926	chr19:6740670-6741203	TRIP10
cg03710719	chr19:36389546-36389967	NFKBID
cg03720581	chr11:506354-508009	RNH1
cg03728046	chr7:6566067-6566650	GRID2IP
cg03729251	chr4:151500764-151501299	LRBA
cg03730428	chr11:32008803-32009044	RCN1
cg03731268	chr1:62660556-62660946	L1TD1
cg03733219	chr19:38886110-38886615	SPRED3
cg03733760	chr11:69517841-69519929	FGF19
cg03734595	chr18:9333897-9334244	
cg03738025	chr6:105388191-105389545	LIN28B; LIN28B-AS1
cg03738707	chr2:128453175-128453446	

cg03740978	chr18:24126781-24131138	KCTD1
cg03753391	chr17:59473061-59483266	TBX2
cg03755177	chr7:44349112-44349716	CAMK2B
cg03762994	chr17:6898821-6900427	ALOX12-AS1
cg03766620	chr2:127783082-127783311	
cg03768777	chr1:213123648-213125092	VASH2
cg03776464	chr2:222436035-222438941	EPHA4
cg03780132	chr5:42951077-42952410	
cg03780851	chr8:102504479-102504841	GRHL2
cg03786933	chr16:69760294-69760506	NQO1
cg03787864	chr16:88716990-88717606	CYBA
cg03803071	chr19:22034395-22034943	ZNF43
cg03804136	chr17:75368689-75370506	SEPTIN9
cg03813164	chr10:11059443-11060524	CELF2
cg03816707	chr20:43726298-43727372	KCNS1
cg03817667	chr7:54609735-54610140	VSTM2A
cg03817911	chr12:104697349-104697984	TXNRD1
cg03825010	chr5:159399005-159399928	ADRA1B
cg03840594	chr6:99279318-99283842	POU3F2
cg03848381	chr15:40573629-40576118	ANKRD63
cg03853987	chr2:101033607-101034296	CHST1
cg03858673	chr6:28583935-28584289	ZBED9
cg03864000	chr11:111410933-111412199	LAYN
cg03884132	chr15:33009531-33011696	GREM1
cg03885037	chr10:135089970-135090491	ADAM8
cg03905847	chr10:134597358-134602649	NKX6-2
cg03929741	chr13:107186469-107189024	EFNB2
cg03932723	chr11:63258379-63258804	HRASLS5
cg03940848	chr1:229542944-229543770	
cg03947459	chr4:1685406-1686743	FAM53A
cg03957481	chr22:50984973-50988141	KLHDC7B
cg03963198	chr5:1881925-1887743	IRX4
cg03970036	chr2:220173871-220174283	PTPRN
cg03976877	chr7:158936508-158938492	VIPR2
cg04008888	chr11:68622723-68623252	
cg04012924	chr11:13689589-13690724	FAR1
cg04014105	chr6:110797298-110798201	SLC22A16
cg04017533	chr18:55019708-55021605	ST8SIA3
cg04021697	chr1:3566446-3569636	TP73
cg04035559	chr10:71077947-71079377	HK1
cg04037038	chr2:183731820-183732093	
cg04065086	chr1:151103686-151106100	SEMA6C
cg04084088	chr19:33165267-33168099	RGS9BP
cg04085025	chr7:30028519-30029822	SCRN1
cg04088969	chr10:11059443-11060524	CELF2
cg04092800	chr5:40679503-40682081	PTGER4
cg04093225	chr1:166916866-166917215	ILRD2
cg04100696	chr11:12029738-12030841	DKK3
cg04111064	chr3:138663719-138666346	FOXL2; FOXL2NB
cg04113200	chr12:131303094-131303836	STX2

cg04118306	chr8:24770909-24772547	NEFM
cg04122873	chr6:28956107-28956578	HCG16
cg04138502	chr3:123166219-123168567	ADCY5
cg04143876	chr15:76638473-76639823	
cg04147049	chr1:33219428-33220028	KIAA1522
cg04148762	chr1:203598472-203598853	ATP2B4
cg04166294	chr16:54317822-54324604	IRX3
cg04167239	chr4:3767073-3769789	ADRA2C
cg04167595	chr8:11555100-11555603	GATA4
cg04176888	chr19:41595896-41596140	CYP2A13
cg04177426	chrX:150863180-150864078	PRRG3
cg04184836	chr15:83315117-83317541	CPEB1
cg04188273	chr4:110222971-110224257	COL25A1
cg04192867	chr10:93647053-93647393	
cg04203238	chr4:16084196-16085735	PROM1
cg04209650	chr17:19648140-19648492	ALDH3A1
cg04217778	chr7:5111621-5112088	RBAK-RBAKDN
cg04221886	chr2:44058766-44059232	ABCG5
cg04223420	chr5:134363093-134365146	PITX1
cg04224064	chr14:36991595-36992488	NKX2-1-AS1
cg04235837	chr3:13114628-13115245	
cg04245057	chr17:59473061-59483266	TBX2
cg04246763	chr1:87617693-87617986	LINC01140
cg04265797	chr14:38678246-38680937	SSTR1
cg04281464	chr14:70014544-70014993	
cg04283162	chr15:29862036-29863649	FAM189A1
cg04285275	chr16:31548738-31549184	
cg04293085	chr20:4202149-4202765	ADRA1D
cg04293902	chr10:134597358-134602649	NKX6-2
cg04299389	chr20:25128765-25129610	LOC284798
cg04316624	chr2:177036255-177037213	HOXD3
cg04317962	chr16:79623492-79623957	
cg04321866	chr13:107186469-107189024	EFNB2
cg04324758	chr19:16435203-16438064	KLF2
cg04328413	chr19:59049779-59050304	ZBTB45
cg04332534	chr19:37095681-37096589	ZNF382
cg04337653	chr6:100905445-100905697	SIM1
cg04344565	chr8:140714586-140718259	KCNK9
cg04359418	chr10:76573196-76573507	
cg04361852	chr11:73693703-73694802	UCP2
cg04362586	chr2:45231782-45232550	SIX2
cg04363624	chr1:47696505-47698197	TAL1
cg04365224	chr3:72788073-72788416	
cg04369341	chr20:42543098-42545137	TOX2
cg04380519	chr17:61776891-61778733	LOC729683
cg04384626	chr4:41882451-41882964	LINC00682
cg04386144	chr6:43970074-43970922	c6orf223
cg04395369	chr19:19738573-19739821	LPAR2
cg04407470	chr6:108485672-108490539	NR2E1
cg04408595	chr3:71802561-71804209	EIF4E3

cg04415176	chr2:176957055-176958279	HOXD13
cg04425920	chr10:50602990-50606783	DRGX
cg04426311	chr3:3840514-3842772	LRRN1
cg04431629	chr1:50884229-50891471	DMRTA2
cg04431946	chr2:47796924-47799166	KCNK12
cg04442576	chr6:118228043-118229811	SLC35F1
cg04453180	chr5:140855387-140856620	PCDHGA1
cg04460984	chr15:78933188-78933821	CHRNB4
cg04467162	chr3:128764541-128766055	
cg04481779	chr6:137365300-137366126	IL20RA
cg04487931	chr1:28199032-28199257	THEMIS2
cg04495670	chr11:69632034-69634710	FGF3
cg04497176	chr19:34972055-34973645	WTIP
cg04502985	chr5:77268351-77268787	
cg04508482	chr4:995483-997541	IDUA
cg04515001	chr6:24357720-24358309	DCDC2; KAAG1
cg04515996	chr6:85472703-85474132	TBX18
cg04528060	chr4:57371583-57372022	ARL9
cg04532033	chr3:157822974-157823836	SHOX2
cg04547675	chr11:13984067-13986349	SPON1
cg04550737	chr1:119529820-119530712	TBX15
cg04552470	chr1:20669567-20670018	VWA5B1
cg04557125	chr4:41883493-41884570	LINC00682
cg04564920	chr3:122296613-122296828	PARP15
cg04573550	chr14:38678246-38680937	SSTR1
cg04580344	chr6:127796287-127797356	SOGA3
cg04590978	chr10:97802872-97804262	ENTPD1-AS1
cg04595393	chrX:102000690-102001558	BHLHB9
cg04597433	chr4:9783036-9784960	DRD5
cg04599403	chr5:926587-927401	
cg04600618	chr6:43612715-43613366	RSPH9
cg04609245	chr4:41646293-41646582	LIMCH1
cg04612444	chr8:57358127-57359415	PENK
cg04618002	chr7:5632336-5634555	FSCN1
cg04618333	chr17:46800534-46800746	PRAC2
cg04621893	chr10:22623351-22625875	
cg04638468	chr4:122685861-122686565	TMEM155
cg04645150	chr6:28583935-28584289	ZBED9
cg04645914	chr19:52452317-52452543	ZNF350-AS1
cg04651781	chr4:1396292-1401730	NKX1-1
cg04652621	chr7:150671807-150672370	KCNH2
cg04654530	chr2:63282515-63283122	OTX1
cg04669574	chr12:50297581-50297988	FAIM2
cg04673590	chr7:27135343-27136736	HOTAIRM1
cg04686953	chr4:1107126-1107728	RNF212
cg04701034	chr14:52534582-52536722	NID2
cg04707408	chr7:27231806-27233097	
cg04710571	chr19:38852312-38853485	CATSPERG
cg04738965	chr3:147126989-147128999	ZIC1
cg04742135	chr4:1800145-1800351	FGFR3

cg04743654	chr3:50377804-50378540	RASSF1
cg04751631	chr7:149916641-149918264	
cg04759439	chr3:56502612-56502815	
cg04766371	chr10:43857487-43858205	
cg04778012	chr7:99774734-99775583	STAG3
cg04779720	chr1:11919116-11919555	
cg04781274	chr16:67034471-67035340	CES4A
cg04784471	chr16:2765620-2765855	PRSS27
cg04786287	chr16:54970302-54972846	
cg04786857	chr2:29033352-29034011	SPDYA
cg04787024	chr2:99552186-99553831	KIAA1211L
cg04792712	chr8:17270604-17271120	MTMR7
cg04797323	chr12:93963964-93967395	SOCS2
cg04804877	chr12:3862069-3862606	CRACR2A
cg04805181	chr15:66914242-66914884	LINC01169
cg04805619	chr3:167097928-167098198	ZBBX
cg04819499	chr17:43044370-43046424	C1QL1
cg04819760	chr10:22764709-22767050	
cg04823311	chr7:28995306-28998541	TRIL
cg04837832	chr10:101290626-101291178	LINC01475
cg04839289	chr14:24044887-24046760	JPH4
cg04863005	chr1:59042014-59043295	TACSTD2
cg04863892	chr7:27182614-27185562	HOXA-AS3
cg04864807	chr2:121411984-121412315	
cg04878000	chr1:32237828-32238661	
cg04895288	chr7:98099883-98100289	
cg04907523	chr1:213123648-213125092	VASH2
cg04917181	chr8:98289605-98290404	LOC101927066
cg04920358	chr8:49468684-49468959	LOC101929268
cg04920951	chr11:67350929-67351953	GSTP1
cg04957628	chr6:137242316-137245442	SLC35D3
cg04970117	chr3:45837481-45838256	SLC6A20
cg04972745	chr10:101287163-101287920	LINC01475
cg04974290	chr17:5000370-5001205	
cg04981611	chr2:47796924-47799166	MSH2
cg04991805	chr10:99789615-99791320	CRTAC1
cg04992127	chr5:2748369-2757024	IRX2; C5orf38
cg04992638	chr17:43339125-43339832	SPATA32; MAP3K14-AS1
cg04998420	chr1:153508425-153508840	S100A6
cg05012676	chr16:88564053-88564394	ZFPM1
cg05020604	chr19:37095681-37096589	ZNF382; ZNF529
cg05022673	chr6:56818874-56820308	DST; BEND6
cg05036656	chr4:41875446-41875794	
cg05040544	chr13:107186469-107189024	EFNB2
cg05045981	chr7:129418287-129422509	
cg05052633	chr17:29248848-29249926	ADAP2
cg05053327	chr17:40837023-40837525	CNTNAP1
cg05057720	chr14:38724255-38725537	CLEC14A
cg05057910	chr7:27260102-27260467	
cg05062087	chr8:145806259-145806713	ARHGAP39

cg05065507	chr1:59042014-59043295	TACSTD2
cg05068987	chr19:12978360-12978785	MAST1
cg05071046	chr16:54325041-54325703	
cg05080154	chr18:76737006-76741244	SALL3
cg05085230	chr6:133562087-133563586	EYA4
cg05095158	chr11:13030331-13032584	RASSF10
cg05099508	chr10:22634001-22634862	SPAG6
cg05106897	chr6:34433412-34434371	PACSIN1
cg05107535	chr16:3242623-3242912	OR1F1
cg05118482	chr16:23193717-23194773	SCNN1G
cg05136263	chr2:45235512-45237792	SIX2
cg05152874	chrX:50556225-50557179	SHROOM4
cg05158615	chr7:24323559-24325080	NPY
cg05166490	chr8:41753341-41755250	ANK1
cg05180443	chr17:48545571-48546900	ACSF2; CHAD
cg05183931	chr11:17717506-17717767	LINC02729
cg05184456	chr8:145555343-145562310	SCRT1
cg05184938	chr17:75368689-75370506	SEPTIN9
cg05196969	chr15:76627524-76627838	
cg05201970	chr5:45695395-45696510	HCN1
cg05211768	chr15:91427541-91427905	FES
cg05233128	chr19:3659642-3659949	PIP5K1C
cg05233674	chr6:34433412-34434371	PACSIN1
cg05237485	chr8:11555100-11555603	GATA4
cg05270634	chr17:41177337-41177593	RND2
cg05297854	chr22:50623165-50625226	TRABD
cg05306745	chr17:76920685-76921961	TIMP2
cg05309948	chr11:2290105-2292932	
cg05327835	chr4:995483-997541	IDUA
cg05336395	chr13:53419898-53422872	PCDH8
cg05337743	chr19:54369388-54369809	MYADM
cg05346841	chr1:14026482-14027200	PRDM2
cg05347898	chr6:24357720-24358309	DCDC2; KAAG1
cg05349062	chr2:74740456-74743795	TLX2
cg05350411	chr4:568514-570488	TMEM271
cg05352500	chr19:34972055-34973645	WTIP
cg05353869	chr11:75139455-75139817	KLHL35
cg05361559	chr8:22547487-22553427	EGR3
cg05362548	chr8:11204826-11205689	TDH
cg05364567	chr17:5019638-5019879	ZNF232
cg05364759	chr1:21835549-21836344	ALPL
cg05372113	chr19:41595896-41596140	CYP2A13
cg05376374	chr11:43602546-43603215	MIR129-2
cg05377226	chr1:171810468-171811325	DNM3
cg05386493	chr3:138663719-138666346	FOXL2; FOXL2NB
cg05413277	chr2:74641734-74643350	C2orf81
cg05415020	chr6:110797298-110798201	SLC22A16
cg05436231	chr1:27709601-27709831	CD164L2
cg05452406	chr6:10881847-10882051	GCM2
cg05457344	chr6:26251899-26252233	HIST1H2BH

cg05475524	chr11:66326640-66326840	ACTN3
cg05488523	chr17:79478236-79482426	ACTG1
cg05490712	chr11:31831621-31839038	PAX6
cg05495949	chr7:4922708-4923576	RADIL
cg05506365	chr2:105468852-105473488	POU3F3
cg05514256	chr2:45231782-45232550	SIX2
cg05519781	chr21:40032244-40033665	ERG
cg05520409	chr16:1029879-1035327	LMF1; SOX8
cg05527869	chr17:35291900-35300875	LHX1
cg05529506	chr18:44526867-44527137	KATNAL2
cg05537653	chr11:47611139-47612438	C1QTNF4
cg05545132	chr2:144694667-144695180	LOC101928386
cg05546863	chr16:66612750-66613412	KLF-CMTM1; CMTM1; CNMT
cg05554936	chr19:11450017-11450414	RAB3D
cg05569109	chr2:166649910-166650966	GALNT3
cg05575054	chr10:115803359-115805468	ADRB1
cg05617413	chr12:133065844-133066393	
cg05618934	chr4:1407447-1408014	
cg05621343	chr11:134145560-134147180	GLB1L3
cg05627083	chr18:10454454-10455268	APCDD1
cg05629186	chr6:27356493-27357020	ZNF391
cg05639679	chr1:33219428-33220028	KIAA1522
cg05661282	chr19:58220190-58220517	ZNF154
cg05663341	chr7:96634623-96634915	DLX6-AS1
cg05675373	chr1:110752257-110754794	KCNC4
cg05684891	chr9:124460968-124461361	DAB2IP
cg05686497	chr5:127872564-127874945	FBN2
cg05697849	chr1:50513645-50514320	ELAVL4
cg05711235	chr8:65498604-65498828	
cg05714219	chr2:31360322-31361821	GALNT14
cg05720261	chr1:40105011-40105707	HEYL
cg05721365	chr17:62774367-62777797	BPTFP1-KPNA2P3; ARHGAP2
cg05732750	chr7:6703504-6704075	AK123300
cg05733135	chr11:27740645-27741236	BDNF
cg05739190	chr10:97802872-97804262	CCNJ; ENTPD1-AS1
cg05751100	chr6:26550783-26551114	
cg05756489	chr10:92616821-92618034	HTR7
cg05766140	chr6:391189-393790	IRF4
cg05768427	chr3:127633994-127634588	KBTBD12
cg05770238	chr1:26686517-26687281	
cg05771369	chr12:58021295-58022037	B4GALNT1
cg05774229	chr11:76032912-76033392	
cg05774978	chr1:146649623-146649852	CHD1L
cg05783139	chr2:198649752-198651599	BOLL
cg05788681	chrX:103498707-103499843	ESX1
cg05789704	chr8:38965014-38965423	ADAM32
cg05804220	chr15:45421237-45422394	DUOXA1; DUOX1
cg05817758	chr11:13030331-13032584	RASSF10
cg05828690	chr8:145751956-145756326	C8orf82
cg05832051	chr19:54369388-54369809	MYADM

cg05835105	chr4:111542063-111544464	PITX2
cg05843841	chr11:76381450-76382295	LRRC32
cg05844937	chr6:1604607-1615866	FOXC1
cg05860723	chr12:81102035-81102716	MYF6
cg05863502	chr9:140771301-140773513	LOC100133077
cg05871997	chr6:56818874-56820308	DST; BEND6
cg05884032	chr18:76737006-76741244	SALL3
cg05904135	chr10:134597358-134602649	NKX6-2
cg05918002	chr17:5019051-5019551	ZNF232; USP6
cg05920961	chr14:102247661-102248279	PPP2R5C
cg05922253	chrX:50213659-50213983	DGKK
cg05922610	chr6:108495655-108495986	NR2E1
cg05928342	chr19:9473590-9474001	ZNF559-ZNF177
cg05931423	chr5:115697135-115697589	
cg05933904	chr16:1029879-1035327	SOX8
cg05933935	chr1:6051596-6052955	NPHP4; KCNAB2
cg05937737	chr3:170303533-170303768	SLC7A14; SLC7A14-AS1
cg05938001	chr2:172952810-172953160	DLX1
cg05939970	chr3:127794370-127796136	RUVBL1; RUVBL1-AS1
cg05945059	chr18:56939625-56941540	RAX
cg05962092	chr19:49575130-49576076	KCNA7
cg05971966	chr5:40679503-40682081	PTGER4; TTC33
cg05977669	chr7:27225051-27225629	HOXA11-AS
cg05981335	chr12:81102035-81102716	MYF6
cg05983315	chr19:58545116-58545897	ZSCAN1
cg05994148	chr6:34433412-34434371	PACSIN1
cg06001519	chr13:23500590-23501398	
cg06007282	chr2:45231212-45231482	
cg06010020	chr7:5111621-5112088	RBAK-RBAKDN; RBAKDN
cg06034933	chr1:228645197-228646434	HIST3H2A
cg06036912	chr16:3067522-3068358	CLDN6
cg06040206	chr2:172959268-172962054	
cg06048524	chr10:44879715-44882391	CXCL12
cg06065125	chr4:85402765-85403175	
cg06073471	chr3:101497831-101498648	NXPE3
cg06081518	chr16:3220439-3221356	
cg06100807	chr15:90029932-90030328	RHCG
cg06122660	chr12:8025365-8025908	SLC2A14
cg06158650	chr1:119522386-119522638	TBX15
cg06169131	chr10:103535637-103536505	FGF8
cg06183338	chr5:178003624-178004247	COL23A1
cg06186698	chr3:138663719-138666346	FOXL2NB; FOXL2
cg06188229	chr1:226411008-226411880	MIXL1
cg06188545	chr1:156863416-156863711	PEAR1
cg06196147	chr3:128712405-128713058	KIAA1257
cg06211893	chr1:171810468-171811325	DNM3
cg06215569	chr1:110610266-110613303	ALX3
cg06218338	chr7:27231806-27233097	
cg06220235	chr16:11348542-11350803	SOCS1
cg06223767	chr6:391189-393790	IRF4

cg06226630	chr4:48492118-48493589	ZAR1
cg06230736	chr10:8091375-8098329	GATA3; GATA3-AS1
cg06239355	chr5:32709911-32714450	NPR3
cg06241292	chr3:49314438-49314815	USP4
cg06246357	chr6:1524154-1524543	
cg06263495	chr11:2290105-2292932	ASCL2
cg06268694	chr22:46929318-46934861	CELSR1
cg06270244	chr15:78933188-78933821	CHRNB4
cg06275859	chr4:1407447-1408014	
cg06291867	chr10:92616821-92618034	HTR7
cg06302025	chrX:102000690-102001558	BHLHB9
cg06304097	chr10:133109194-133111052	TCERG1L
cg06319822	chr16:214344-216720	HBM
cg06329574	chr11:69632034-69634710	FGF3
cg06338710	chr1:92945908-92952609	GFI1
cg06341513	chr11:61275826-61277329	LRRC10B
cg06346974	chr1:65775019-65775746	DNAJC6
cg06355129	chr1:156646293-156647260	NES
cg06365535	chr17:59531724-59535254	TBX4
cg06366062	chr17:30813178-30815239	CDK5R1
cg06367311	chr16:57317968-57318964	PLLP
cg06368590	chr8:116679699-116679936	TRPS1
cg06372962	chr1:173638663-173639045	ANKRD45
cg06376520	chr12:81110726-81111126	MYF5
cg06376715	chr1:3634634-3635101	TP73
cg06377278	chr1:25255528-25259005	RUNX3
cg06382344	chr2:162279836-162280709	TBR1
cg06382559	chr10:102896343-102896665	TLX1
cg06390536	chr17:73583839-73586337	MYO15B
cg06391932	chr2:45159894-45160112	LINC01833
cg06392169	chr6:391189-393790	IRF4
cg06396724	chr14:61188117-61190772	SIX4
cg06399148	chr8:104152727-104153390	BAALC
cg06404175	chr20:3052098-3053103	OXT
cg06425919	chr11:17740790-17743779	MYOD1
cg06428055	chrX:129243675-129245575	ELF4
cg06431702	chr11:66623621-66626614	LRFN4; PC
cg06433023	chr6:108485672-108490539	NR2E1
cg06444282	chr1:20669567-20670018	VWA5B1
cg06445348	chr1:166916866-166917215	ILDR2
cg06454760	chr19:58570394-58571779	ZNF135
cg06458358	chr8:1771362-1772760	ARHGEF10
cg06463958	chr6:166579974-166583423	TBXT
cg06469345	chr4:9783036-9784960	DRD5
cg06470822	chr6:28175283-28175605	
cg06472476	chr19:54369388-54369809	MYADM
cg06488443	chr2:162279836-162280709	TBR1
cg06523224	chr15:83951981-83953930	BNC1
cg06525651	chr10:128993506-128995167	DOCK1
cg06535115	chr6:26550783-26551114	

cg06552366	chr2:161263872-161264460	RBMS1
cg06558014	chr6:100051019-100051972	
cg06561329	chr22:17600564-17602611	LINC01664
cg06570224	chr3:157812054-157812764	
cg06572160	chr19:50831455-50832070	KCNC3
cg06572465	chr5:140800761-140801072	PCDHGA1
cg06575065	chr16:68676451-68676843	
cg06594404	chr4:379900-380399	
cg06598091	chr12:11653233-11653775	
cg06610849	chr19:58520755-58521224	
cg06611810	chr19:41316544-41317318	CYP2T1P
cg06616710	chr10:99473085-99473291	
cg06632762	chr1:110230239-110230614	GSTM1
cg06644428	chr2:233283398-233285959	
cg06647751	chr10:88149267-88149738	
cg06648277	chr10:134597358-134602649	NKX6-2
cg06655810	chr7:27260102-27260467	
cg06657050	chr15:53080459-53083699	ONECUT1
cg06665109	chr19:54369388-54369809	MYADM
cg06666025	chr16:66612750-66613412	KLF-CMTM1; CMTM1; CNMT
cg06673178	chrX:50556225-50557179	SHROOM4
cg06680243	chr14:103655242-103655928	LINC00605
cg06686029	chr20:30777757-30778329	TSPY26P
cg06720669	chrX:108867763-108868486	KCNE5
cg06741043	chr15:68870634-68871974	CORO2B
cg06749789	chr2:242549374-242549995	THAP4
cg06763054	chr8:17270604-17271120	MTMR7
cg06769546	chr10:134597358-134602649	NKX6-2
cg06779449	chr20:42543098-42545137	TOX2
cg06782035	chr5:16179065-16180420	MARCH11; MARCHF11
cg06782692	chr10:134120448-134122422	STK32C
cg06784108	chr13:107186469-107189024	EFNB2
cg06790860	chr16:69760294-69760506	NQO1
cg06793562	chr6:85482570-85484718	
cg06818307	chr20:6750696-6750957	BMP2
cg06818605	chr7:148982080-148982675	LOC155060; ZNF783
cg06825142	chr11:636907-640628	DRD4
cg06829686	chr1:47909713-47911020	
cg06829830	chr5:80255905-80257006	RASGRF2-AS1
cg06848185	chr17:75368689-75370506	SEPTIN9
cg06864047	chr7:97839863-97840222	
cg06871074	chr1:146551477-146551764	
cg06893138	chr1:228645197-228646434	HIST3H2A
cg06894334	chr4:1396292-1401730	NKX1-1
cg06897264	chr6:28979247-28979499	
cg06905692	chr2:30142832-30144624	ALK
cg06905726	chr19:18209912-18210268	MAST3
cg06907708	chr19:18335073-18337375	PDE4C
cg06913330	chr4:2059929-2063181	NAT8L
cg06917617	chr1:207818390-207818952	CR1L

cg06921552	chr11:69258150-69258544	
cg06922635	chr4:57371583-57372022	ARL9
cg06933574	chr5:134368579-134370466	PITX1
cg06936155	chr1:91190490-91192804	
cg06943667	chr11:12029738-12030841	DKK3
cg06947913	chr12:50297581-50297988	FAIM2
cg06951245	chr2:209271338-209271987	PTH2R
cg06953252	chr1:40782221-40783145	COL9A2
cg06954658	chr18:76737006-76741244	SALL3
cg06962177	chr1:63782395-63790471	FOXD3
cg06962944	chr20:42543098-42545137	TOX2
cg06966811	chr14:57274608-57276840	OTX2
cg06973615	chr2:175208416-175209076	
cg06982190	chr7:27199662-27200960	
cg06984903	chr16:73099813-73100791	
cg06992285	chr8:99952021-99954686	STK3
cg06995920	chr12:64061837-64062774	DPY19L2
cg07005055	chr1:113050814-113052301	WNT2B
cg07014523	chr20:50721311-50721912	ZFP64
cg07030339	chr15:101419262-101421133	ALDH1A3
cg07036412	chr6:24360030-24360411	DCDC2
cg07036524	chr18:77557781-77558948	
cg07039560	chr5:140683196-140683773	SLC25A2
cg07044708	chr15:75470912-75471212	
cg07049552	chr16:30204696-30206485	SLX1A
cg07060551	chr19:51198144-51198460	SHANK1
cg07061260	chr16:68676451-68676843	CDH3
cg07065111	chr2:5831188-5831413	
cg07065220	chr3:193987426-193987758	
cg07068756	chr4:41258760-41259867	UCHL1
cg07071389	chr20:30777757-30778329	TSPY26P
cg07074543	chr3:119041383-119042255	ARHGAP31
cg07076076	chr16:1458144-1458435	UNKL
cg07095995	chr4:110222971-110224257	COL25A1
cg07095997	chr14:61188117-61190772	SIX4
cg07113642	chr20:2780979-2781497	CPXM1
cg07124687	chr6:28956107-28956578	
cg07128416	chr3:113160300-113160641	WDR52
cg07137955	chr5:42992034-42992955	AK056817
cg07138355	chr6:26550783-26551114	
cg07139509	chr14:70038109-70040302	CCDC177
cg07149609	chr1:170630457-170630851	PRRX1
cg07166654	chr12:52652019-52652743	KRT86
cg07167423	chr1:36042433-36043444	TFAP2E
cg07171111	chr4:10462833-10463689	ZNF518B
cg07203423	chr2:74740456-74743795	TLX2
cg07204280	chr18:12254148-12255089	CIDEA
cg07212778	chr2:119914127-119916663	C1QL2
cg07219542	chr1:110210582-110210956	GSTM2
cg07230107	chr1:224803638-224805314	CNIH2

cg07242540	chr1:12600348-12600556	
cg07247419	chr20:21376359-21378245	NKX2-4
cg07251711	chr10:134597358-134602649	NKX6-2
cg07265743	chr17:59531724-59535254	TBX4
cg07274716	chr5:134363093-134365146	PITX1
cg07276415	chr11:46354092-46355190	DGKZ
cg07279070	chr2:73143056-73148260	EMX1
cg07333715	chr11:2290105-2292932	ASCL2
cg07336350	chr16:54317822-54324604	IRX3
cg07339783	chr2:176952696-176953297	
cg07340580	chr7:107301206-107302416	SLC26A4
cg07347092	chr12:124246525-124247254	DNAH10
cg07352345	chr5:175792356-175793505	ARL10
cg07363880	chr16:29465354-29467143	SLX1A
cg07371589	chr1:151103686-151106100	SEMA6C
cg07376547	chr1:48058794-48059230	
cg07379055	chr19:12305553-12306304	
cg07380496	chr5:71402878-71404868	MAP1B
cg07382554	chr18:76737006-76741244	SALL3
cg07382920	chr1:3566446-3569636	TP73
cg07399369	chr3:142837886-142840838	CHST2
cg07406897	chr17:27346854-27347222	
cg07420190	chr1:220701508-220702348	MARK1
cg07435294	chr11:65553750-65555573	OVOL1
cg07437373	chr3:134125488-134125871	
cg07451080	chr3:44036408-44041104	
cg07451524	chr10:123922851-123923542	TACC2
cg07462756	chr4:4859633-4860191	
cg07484354	chr5:157078328-157079564	SOX30
cg07489048	chr14:29236836-29237832	FOXG1
cg07495363	chr2:198649752-198651599	BOLL
cg07512361	chr7:101943786-101944557	SH2B2
cg07513622	chr7:18125915-18127009	HDAC9
cg07515989	chr1:43250763-43251082	TMEM269
cg07519536	chr6:26501726-26502107	BTN1A1
cg07519816	chr18:53447075-53447842	LINC01415
cg07520506	chr12:7592407-7593030	CD163L1
cg07522644	chr6:43142014-43142217	SRF
cg07522913	chr7:27150031-27150418	HOXA3
cg07523553	chr11:1768980-1770712	IFITM10
cg07528613	chr19:53073309-53074039	ZNF701
cg07533148	chr1:248020331-248021252	TRIM58
cg07539798	chr5:140810495-140812617	PCDHGA1
cg07540103	chr17:6679206-6679710	FBXO39
cg07544187	chr19:19650684-19651274	CILP2
cg07558472	chr5:139047906-139048235	CXXC5
cg07561017	chr12:48397890-48398731	COL2A1
cg07565505	chr5:1881925-1887743	IRX4
cg07566169	chr2:176973428-176973718	HOXD11
cg07570142	chr6:132722094-132722901	MOXD1

cg07573209	chr15:76630030-76630970	ISL2
cg07584066	chr17:57642721-57643294	DHX40
cg07586008	chr19:49636225-49636694	PPFIA3
cg07589773	chr7:50343758-50344519	IKZF1
cg07595776	chr5:77253833-77254049	LOC101929154
cg07599133	chr19:35068407-35068897	SCGB1B2P
cg07600636	chr11:13030331-13032584	RASSF10
cg07622404	chr3:101497831-101498648	NXPE3
cg07636117	chr2:237071795-237078762	GBX2
cg07641160	chr6:117085740-117086942	FAM162B
cg07671858	chr10:75407414-75407706	SYNPO2L
cg07687119	chr12:54423428-54423712	HOXC6
cg07688604	chr12:133065844-133066393	
cg07689503	chr2:74425445-74426423	MTHFD2
cg07696699	chr4:17782643-17783624	FAM184B
cg07697597	chr22:43738883-43740285	SCUBE1
cg07697895	chr7:116962842-116964803	WNT2
cg07702750	chr7:28995306-28998541	TRIL
cg07703401	chr16:230302-231761	HBQ1
cg07721203	chr15:58357310-58358776	ALDH1A2
cg07723510	chr6:30227321-30228255	HCG17
cg07766263	chr3:170303045-170303249	SLC7A14; SLC7A14-AS1
cg07779120	chr15:99190447-99194559	IGF1R
cg07783282	chr12:95941907-95942979	USP44
cg07790615	chr1:237205127-237206644	RYR2
cg07791578	chr6:24357720-24358309	DCDC2
cg07799366	chr6:52226754-52228006	PAQR8
cg07800665	chr19:21657570-21658035	
cg07813142	chr2:171569878-171573904	SP5; LOC101926913
cg07816687	chr16:67196793-67199988	FBXL8; HSF4
cg07822928	chr19:615692-623505	HCN2
cg07835424	chr2:200320503-200329681	SATB2
cg07844931	chr16:58018401-58019471	TEPP
cg07846311	chr4:57371583-57372022	ARL9
cg07847863	chr13:53419898-53422872	PCDH8
cg07850527	chr16:89267824-89268087	
cg07850604	chr14:36002599-36005013	INSM2
cg07852757	chr10:64574661-64578778	EGR2
cg07860213	chr8:70981874-70984888	PRDM14
cg07871590	chr7:127671159-127672853	SND1
cg07878486	chr19:58951215-58952250	ZNF132
cg07879739	chr22:38220590-38221619	GALR3
cg07882671	chr5:1881925-1887743	IRX4
cg07888856	chr6:137242316-137245442	SLC35D3
cg07891531	chr16:3220439-3221356	
cg07892597	chr1:119531992-119532196	TBX15
cg07904475	chr4:151500764-151501299	LRBA
cg07920503	chr13:25743999-25746127	AMER2
cg07926482	chr16:84650961-84651565	COTL1
cg07931391	chr13:100608123-100609686	LOC101927437

cg07936950	chr2:172952810-172953160	DLX1
cg07938743	chr2:63283937-63284147	OTX1
cg07943461	chr15:32604648-32607825	DKFZp434L187
cg07951347	chr6:27463177-27463379	
cg07952813	chr2:39186778-39187968	ARHGEF33
cg07960067	chr2:9143128-9144630	MBOAT2
cg07973435	chr6:100905445-100905697	SIM1
cg07974511	chr2:63282515-63283122	OTX1
cg07983905	chr20:3073225-3074232	
cg07987843	chr12:3308813-3310270	TSPAN9
cg07990546	chr2:74740456-74743795	TLX2
cg07990749	chr10:47008085-47008410	
cg07993547	chr19:18209912-18210268	MAST3
cg08017858	chr11:74022429-74022703	P4HA3
cg08024029	chr1:20669567-20670018	VWA5B1
cg08032924	chr16:66612750-66613412	CMTM1; CMTM2
cg08034070	chr17:40805675-40805957	
cg08034379	chr2:228736231-228736544	DAW1
cg08042316	chr7:139930066-139930356	
cg08047907	chr1:169396622-169396869	CCDC181
cg08049853	chr4:41875446-41875794	
cg08065231	chr19:9473590-9474001	ZNF559-ZNF177
cg08066943	chr3:147136904-147137328	
cg08074534	chr6:100895008-100895246	SIM1
cg08074851	chr2:47796924-47799166	KCNK12
cg08081805	chr19:49242019-49242962	RASIP1
cg08100565	chr3:140660335-140661602	SLC25A36
cg08101407	chr20:30777757-30778329	TSYP26P
cg08104202	chr1:169396622-169396869	CCDC181
cg08107689	chr15:29033879-29034710	LOC100289656
cg08126211	chr6:24357720-24358309	DCDC2; KAAG1
cg08136772	chr13:53419898-53422872	PCDH8
cg08139247	chr14:38724255-38725537	CLEC14A
cg08146483	chr1:75601999-75602878	LHX8
cg08154437	chr19:17007102-17008857	CPAMD8
cg08160350	chr4:995483-997541	IDUA
cg08162372	chr14:54420185-54422958	BMP4
cg08165971	chr2:233791784-233793922	NGEF
cg08171351	chr22:17600564-17602611	CECR6
cg08173692	chr6:166720240-166722275	PRR18
cg08187418	chr2:74425445-74426423	MTHFD2
cg08196842	chr14:65016517-65016909	PPP1R36
cg08196968	chr19:38746639-38747379	PPP1R14A
cg08204023	chr5:37834672-37835128	GDNF
cg08207377	chr19:19280999-19281560	MEF2B; BORCS2-MEF2B
cg08214808	chr11:45921388-45922167	MAPK8IP1
cg08218734	chr10:128993506-128995167	DOCK1
cg08226111	chr14:36002599-36005013	INSM2
cg08228715	chr17:79894669-79895533	PYCR1
cg08231709	chr8:99438693-99440425	KCNS2

cg08235864	chr5:2748369-2757024	IRX2; c5orf38
cg08247376	chr17:43339125-43339832	SPATA32; MAP3K14-AS1
cg08249424	chr6:4775132-4777550	CDYL
cg08258000	chr19:49652213-49654260	PPFIA3
cg08258526	chr8:49647703-49647988	EFCAB1
cg08274637	chr13:51417372-51418149	DLEU7; DLEU-AS1
cg08289140	chr1:1534332-1536136	FNDC10
cg08296680	chr17:80186136-80189990	SLC16A3
cg08296831	chr11:65553750-65555573	OVOL1
cg08303146	chr11:2466248-2466818	KCNQ1
cg08305551	chr19:12978360-12978785	MAST1
cg08307816	chr14:100625320-100626461	DEGS2
cg08315202	chr7:98245806-98247759	NPTX2
cg08328777	chr15:45421237-45422394	DUOXA1
cg08343042	chr2:135475699-135476993	TMEM163
cg08347183	chr10:8091375-8098329	GATA3
cg08347500	chr16:54315603-54316104	
cg08350776	chr5:54516268-54516919	MCIDAS
cg08369368	chr5:176558853-176561652	NSD1
cg08376141	chr6:32116591-32117229	PRRT1
cg08382774	chr10:134597358-134602649	NKX6-2
cg08393041	chr3:147108512-147111703	ZIC4
cg08404009	chr13:109792494-109793699	MYO16
cg08407114	chr5:36690208-36690658	
cg08423533	chr10:28030183-28035211	MKX
cg08430009	chr22:19136294-19138512	GSC2
cg08430329	chr2:73429284-73430647	NOTO
cg08441806	chr10:134597358-134602649	NKX6-2
cg08444220	chr10:134597358-134602649	NKX6-2
cg08445802	chr6:85482570-85484718	
cg08452658	chr15:83775862-83776922	TM6SF1
cg08460435	chr1:109203594-109204378	HENMT1
cg08461949	chr19:52104419-52104998	
cg08464190	chr16:49314038-49316543	CBLN1
cg08465862	chr10:3108651-3111419	PFKP
cg08482682	chr11:107798959-107799980	RAB39A
cg08489324	chrX:52004916-52005456	
cg08493294	chr2:25499764-25500429	DNMT3A
cg08494905	chr6:37663762-37667102	MDGA1
cg08495115	chr15:33009531-33011696	GREM1
cg08496953	chr17:42733487-42734626	MEIOC; C17orf104
cg08513472	chr6:1624186-1625468	GMDS
cg08526991	chr2:5831188-5831413	LINC01248
cg08538258	chr21:46706692-46707049	POFUT2
cg08548396	chr6:30095174-30095610	
cg08551725	chr7:30028519-30029822	SCRN1
cg08553437	chr4:122685861-122686565	TMEM155
cg08555653	chr3:138656628-138659107	LINC01391
cg08557649	chr15:76638473-76639823	
cg08558397	chr7:751713-752150	PRKAR1B

cg08572611	chr7:100253783-100254150	ACTL6B
cg08578136	chr2:207139337-207140031	ZDBF2
cg08582356	chr8:86350766-86351196	CA3
cg08584627	chr2:98703355-98703889	VWA3B
cg08592305	chr19:12978360-12978785	MAST1
cg08607018	chr19:31839636-31843049	TSHZ3
cg08613144	chr2:133426653-133428870	LYPD1
cg08616702	chr6:108485672-108490539	NR2E1
cg08631504	chr15:89942593-89943853	LOC105371031
cg08632164	chr7:65970023-65971421	
cg08637618	chr7:27278946-27279469	
cg08640609	chr1:7764594-7765856	CAMTA1
cg08640634	chr6:108485672-108490539	NR2E1
cg08655589	chr3:14443630-14444577	SLC6A6
cg08681432	chr16:23193717-23194773	SCNN1G
cg08682544	chr16:66638255-66639561	CMTM3
cg08690859	chr2:200333688-200334172	SATB2; SATB2-AS1
cg08694014	chr16:68480865-68482822	SMPD3
cg08701621	chr19:58570394-58571779	ZNF135
cg08703872	chr1:151812254-151812525	C2CD4D; C2CD4D-AS1
cg08706670	chr8:99438693-99440425	KCNS2
cg08714590	chr7:90893568-90896683	FZD1
cg08734647	chr16:1921898-1922426	MEIOB
cg08741214	chr2:74425445-74426423	MTHFD2
cg08749305	chr6:106441987-106442912	
cg08753373	chr10:133998913-134001500	DPYSL4
cg08763705	chr5:139525538-139526227	
cg08768852	chr3:69591012-69592050	FRMD4B
cg08772752	chr3:48631883-48632901	COL7A1
cg08777316	chr5:158531376-158531648	LINC02202
cg08781140	chr17:73583839-73586337	MYO15B
cg08789053	chr6:28979247-28979499	
cg08793877	chr2:71115928-71116412	LINC01143
cg08812897	chrX:20284128-20286811	RPS6KA3
cg08827307	chr8:38965014-38965423	ADAM32
cg08832624	chr7:98099883-98100289	
cg08832906	chr7:139208443-139209069	CLEC2L
cg08833577	chr10:118899248-118900329	
cg08844848	chr3:3840514-3842772	LRRN1
cg08848774	chr17:43047548-43047954	
cg08854306	chr4:91048412-91049682	CCSER1
cg08857144	chr2:70994448-70995385	ADD2
cg08858437	chr3:142837886-142840838	CHST2
cg08862890	chr5:169064271-169064702	DOCK2
cg08863953	chr10:22540708-22542739	LOC100130992
cg08871855	chr2:88751336-88752865	FOXI3
cg08876434	chr11:13030331-13032584	RASSF10
cg08890418	chr1:21043833-21044771	KIF17
cg08892600	chr1:223536843-223538628	SUSD4
cg08892705	chr15:99645031-99646444	SYNM

cg08894761	chr2:31805294-31806403	SRD5A2
cg08902977	chr16:57317968-57318964	PLLP
cg08934846	chr16:3067522-3068358	CLDN6
cg08938584	chr5:140797163-140797701	2,3,4,5,6,7,8,9,10; PCDHGB1,
cg08940787	chr17:62774367-62777797	3PTFP1-KPNA2P3 y ARHGAP2
cg08949408	chr10:16561605-16563822	C1QL3
cg08956724	chr16:29624388-29625391	SMG1P2
cg08964780	chr7:27208872-27209616	HOXA10-HOXA9; HOXA10-AS
cg08988797	chr8:102505513-102506430	GRHL2
cg08992305	chr4:165878037-165878446	FAM218A
cg09011231	chr2:133013778-133015740	ANKRD30BL
cg09024126	chr4:16084196-16085735	PROM1
cg09053680	chr10:135043222-135044560	UTF1
cg09054633	chr5:136834017-136835146	SPOCK1
cg09059319	chr8:99985734-99986983	
cg09062550	chr20:20344401-20350605	INSM1
cg09082903	chr4:164252955-164253471	NPY1R
cg09090724	chr3:132756986-132757895	TMEM108
cg09118312	chr12:21810489-21810766	LDHB
cg09142313	chr5:72676121-72678421	
cg09149648	chr20:44642095-44642406	MMP9; SLC12A5-AS1
cg09151754	chr17:5019051-5019551	ZNF232; USP6
cg09163958	chr1:213123648-213125092	VASH2
cg09172548	chr12:3862069-3862606	CRACR2A; EFCAB4B
cg09177106	chr11:69451137-69458596	CCND1
cg09187505	chr6:106433985-106434459	
cg09187933	chr12:127630512-127631199	
cg09193347	chr15:79381805-79384209	RASGRF1
cg09200738	chr15:30864783-30865586	ULK4P2
cg09224821	chr17:80329067-80330208	
cg09229918	chr16:2041826-2043026	SYNGR3
cg09232937	chr5:3594468-3603054	IRX1
cg09233013	chr16:66638255-66639561	CMTM3
cg09236163	chr15:81426500-81426702	CFAP161; C15orf26
cg09241929	chr20:57463653-57467739	GNAS
cg09248054	chr1:967967-970238	AGRN
cg09252528	chr19:42901017-42901375	LIPE-AS1
cg09253581	chr19:44302666-44303176	LYPD5
cg09257635	chr1:228345378-228346700	GJC2
cg09258813	chr8:37822487-37824008	ADRB3
cg09260089	chr10:134597358-134602649	NKX6-2
cg09270675	chr6:56818874-56820308	DST y BEND6
cg09275704	chr16:54316904-54317149	
cg09279988	chr3:196255496-196256013	
cg09323727	chr2:176936247-176936809	
cg09336406	chr2:20068429-20068907	LINC00954
cg09341491	chr16:50707292-50708041	SNX20
cg09341793	chr14:94405236-94406170	ASB2
cg09344183	chr2:175204844-175207553	
cg09371281	chr9:8857486-8858708	PTPRD

cg09374293	chr21:48081242-48081849	PRMT2
cg09374949	chr19:18543829-18549161	ISYNA1; SSBP4
cg09392940	chr2:109744586-109746833	SH3RF3; SH3RF3-AS1
cg09403666	chr10:23461301-23461610	
cg09418984	chr19:54369388-54369809	MYADM
cg09420439	chr7:27135343-27136736	HOTAIRM1
cg09422450	chr16:79623492-79623957	
cg09433135	chr19:46001831-46002686	PPM1N
cg09461837	chr7:3341451-3341733	SDK1
cg09465698	chr5:140787448-140788044	PCDHGA1
cg09472203	chr15:83378008-83379068	AP3B2
cg09476130	chr1:159869902-159870143	CFAP45; CCDC19
cg09480047	chr16:54325041-54325703	
cg09484039	chr2:183730809-183731357	FRZB
cg09489306	chr19:44203559-44203987	
cg09489445	chr19:12203029-12203503	ZNF788P
cg09492451	chr16:54962423-54967805	IRX5; CRNDE
cg09493505	chr7:49813009-49815752	VWC2
cg09504320	chr1:157164539-157165135	
cg09511896	chr1:228246632-228247197	WNT3A
cg09515953	chr19:38746639-38747379	PPP1R14A
cg09523275	chr6:28226980-28227483	NKAPL
cg09534159	chr4:10458130-10459353	ZNF518B
cg09537620	chr11:31825744-31826967	PAX6
cg09554951	chr11:2890389-2891337	KCNQ1DN
cg09556826	chr6:28956107-28956578	HCG15
cg09557462	chr8:26721643-26724566	ADRA1A
cg09567439	chr15:60296136-60298520	FOXB1
cg09570682	chr6:106429112-106429772	
cg09578475	chr19:9473590-9474001	ZNF559-ZNF177
cg09614300	chr10:124894076-124897366	HMX3
cg09614653	chr10:22540708-22542739	LOC100130992
cg09616463	chr15:72611947-72612802	CELF6
cg09617579	chr11:82443436-82444976	FAM181B
cg09635053	chr16:1029879-1035327	LMF1 y SOX8
cg09636525	chr4:7052318-7052613	TADA2B
cg09639151	chr5:140810495-140812617	4,5,6,7,8,9,10,11,12; PCDHG
cg09642925	chr2:114260096-114261794	
cg09645574	chr6:42879280-42879623	
cg09649610	chr1:235811842-235814066	GNG4
cg09652652	chr3:194406441-194409045	FAM43A
cg09654997	chr11:46259265-46260048	
cg09655666	chr17:34819845-34820754	
cg09656389	chr11:31839364-31839813	PAX6-AS1; PAX6
cg09656848	chr11:61275826-61277329	LRRC10B
cg09661010	chr10:73846807-73848233	SPOCK2
cg09661370	chr7:27225051-27225629	HOXA11-AS1
cg09667001	chr17:60214776-60216785	
cg09686443	chr3:126113548-126113967	CFAP100; CCDC37-DT
cg09691340	chr17:42402788-42403266	

cg09695735	chr17:58498712-58499332	
cg09700085	chr3:45837481-45838256	SLC6A20
cg09737314	chr17:6898821-6900427	ALOX12; ALOX12-AS1
cg09753657	chr1:159869902-159870143	CFAP45; CCDC19
cg09754845	chr7:1408517-1408878	
cg09761040	chr19:13208344-13210525	LYL1; NFIX
cg09765089	chr7:27291120-27292197	
cg09768093	chr1:32930501-32931061	ZBTB8B
cg09774842	chr20:3052098-3053103	OXT
cg09775582	chr17:45810590-45811388	TBX21
cg09785344	chr15:101389733-101390260	LOC105369201
cg09792926	chr16:49314038-49316543	CBLN1
cg09793584	chr1:156390404-156391581	C1orf61
cg09799983	chr2:38301277-38304518	CYP1B1; CYP1B1-AS1
cg09812070	chr4:46995129-46995872	GABRA4
cg09813525	chr13:53419898-53422872	PCDH8
cg09827761	chr15:66947361-66947574	LINC01169
cg09848789	chr1:85462586-85463435	MCOLN2
cg09849405	chr16:66612750-66613412	KLF-CMTM1; CMTM1; CNMT
cg09851951	chr16:54317822-54324604	IRX3
cg09854734	chr16:66612750-66613412	KLF-CMTM1; CMTM1; CNMT
cg09858188	chr7:156810573-156814593	
cg09866102	chr16:31213567-31214287	PYCARD-AS1; PYCARD
cg09868451	chr2:74425445-74426423	MTHFD2
cg09871079	chr1:33358470-33359449	HPCA
cg09885735	chr15:60296136-60298520	FOXB1
cg09887059	chr12:113916434-113916717	LHX5-AS1
cg09894698	chr11:2290105-2292932	ASCL2
cg09907936	chr19:58570394-58571779	ZNF135
cg09921610	chr4:41258760-41259867	UCHL1
cg09935388	chr1:92945908-92952609	GFI1
cg09938462	chr22:19705511-19706557	SEPTIN5; SEPT5-GP1BB
cg09940032	chr8:142215692-142216299	
cg09952395	chr11:63258379-63258804	HRASLS5
cg09969277	chr15:89920794-89922768	MIR9-3HG
cg09979478	chr8:145925411-145926101	
cg09980058	chr19:18898229-18898449	COMP
cg09992116	chr10:71077947-71079377	HK1
cg10000952	chr11:116450660-116451912	
cg10013716	chr3:179168736-179169593	GNB4
cg10023272	chr1:110610266-110613303	ALX3
cg10034364	chr13:28491489-28492518	
cg10034890	chr2:230578063-230579981	DNER
cg10043865	chr17:35291900-35300875	LHX1 y LHX1-DT
cg10056132	chr10:50887566-50887862	C10orf53
cg10064339	chr11:73693703-73694802	UCP2
cg10084644	chr7:99774734-99775583	STAG3
cg10091335	chr10:43332388-43332817	
cg10091994	chr12:4378367-4382222	CCND2; CCND2-AS1
cg10094078	chr19:1465207-1471241	APC2

cg10106804	chr1:35258565-35258791	GJA4
cg10113101	chr7:30028519-30029822	SCRN1
cg10119082	chr7:98990158-98990922	ARPC1B
cg10122865	chr2:63283937-63284147	OTX1
cg10132208	chr19:58545116-58545897	ZSCAN1
cg10154122	chr19:38754847-38755699	SPINT2
cg10162691	chr17:41177337-41177593	RND2
cg10168149	chr2:200335498-200336413	SATB2; SATB2-AS1
cg10169241	chr19:1465207-1471241	APC2
cg10171347	chr10:134597358-134602649	NKX6-2
cg10171448	chr10:134597358-134602649	NKX6-2
cg10181419	chr2:263401-265238	chr2:263401-265238
cg10182697	chr10:134597358-134602649	NKX6-2
cg10184889	chr8:86350766-86351196	CA3
cg10188823	chr22:29875920-29877242	NEFH
cg10191210	chr5:42992034-42992955	
cg10192893	chr4:41747509-41747944	PHOX2B
cg10222027	chr16:31580560-31581023	YBX3P1
cg10253847	chr6:28226980-28227483	NKAPL; ZKSCAN4
cg10269548	chr4:53728038-53729000	RASL11B
cg10276465	chr22:42470036-42470669	PHETA2
cg10277651	chr20:623089-623398	
cg10290276	chr11:2290105-2292932	ASCL2
cg10297491	chr10:11059443-11060524	CELF2
cg10304922	chr12:133065844-133066393	
cg10333808	chr12:22486836-22488666	ST8SIA1
cg10334767	chr4:10458130-10459353	ZNF518B
cg10344081	chr4:76555367-76556079	CDKL2
cg10347032	chr3:183542497-183543804	MAP6D1
cg10355837	chr17:64961008-64962321	CACNG4
cg10356613	chr17:35291900-35300875	LHX1; LHX1-DT
cg10363661	chr16:89641054-89643415	CPNE7
cg10374084	chr19:50666148-50666514	IZUMO2
cg10375078	chr10:22623351-22625875	
cg10376598	chr19:19738573-19739821	LPAR2
cg10379890	chr6:391189-393790	IRF4
cg10386298	chr1:221050449-221050864	HLX-AS1
cg10387890	chr1:47489227-47489633	CYP4X1
cg10389229	chr12:66582696-66583345	IRAK3
cg10397440	chr8:57358127-57359415	PENK
cg10409981	chr8:99984585-99985072	
cg10420161	chr10:102899823-102900263	TLX1NB
cg10428938	chr7:98990158-98990922	ARPC1B
cg10429922	chr15:33009531-33011696	GREM1
cg10435816	chr5:140797163-140797701	2,3,4,5,6,7,8,9,10; PCDHGB1,
cg10451772	chr3:127347683-127348859	PODXL2
cg10454246	chr4:41883493-41884570	LINC00682
cg10479082	chr7:28995306-28998541	TRIL
cg10480343	chr15:48936811-48938577	FBN1
cg10481660	chr7:155246391-155251955	EN2

cg10485664	chr7:121945346-121946235	FEZF; FEZF-AS1
cg10501093	chr14:103593235-103593923	TNFAIP2
cg10505257	chr16:4731562-4731835	MGRN1
cg10507275	chr3:3840514-3842772	LRRN1
cg10508760	chr1:166853254-166853790	
cg10511618	chr4:113435899-113438002	NEUROG2; NEUROG2-AS1
cg10521852	chr19:19738573-19739821	LPAR2
cg10523966	chr1:39956425-39958137	BMP8A
cg10530851	chr14:37049334-37051726	NKX2-8
cg10530883	chr5:3594468-3603054	IRX1
cg10534507	chr15:82335473-82339937	MEX3B
cg10541864	chr4:174450047-174451469	HAND2; HAND2-AS1
cg10546219	chr17:29335398-29336452	
cg10555583	chr20:1783825-1784552	
cg10556064	chr16:68480865-68482822	SMPD3
cg10572355	chr1:119549145-119551320	
cg10572969	chr11:13689589-13690724	FAR1; FAR1-IT1
cg10573386	chr3:48698336-48701667	CELSR3
cg10581449	chrX:51238707-51240104	NUDT11
cg10583297	chr15:45421237-45422394	DUOXA1; DUOX1
cg10598034	chr19:53073309-53074039	ZNF701
cg10599693	chr2:24300061-24300294	FAM228B; SF3B6
cg10601287	chr11:119293321-119293943	THY1; USP2-AS1
cg10603275	chr3:238392-240140	CHL1; CHL1-AS2
cg10605681	chr6:30227321-30228255	HLA-L; HCG17
cg10608596	chr19:11785043-11785282	ZNF833P
cg10611186	chr17:30813178-30815239	CDK5R1
cg10617909	chr5:180527369-180528782	
cg10623840	chr3:194406441-194409045	FAM43A
cg10633601	chr1:47696505-47698197	TAL1
cg10634619	chr1:3662964-3664085	TP73-AS1
cg10635122	chr19:52390842-52391368	ZNF577; ZNF649-AS1
cg10637512	chr15:99645031-99646444	SYNM
cg10659805	chr7:96631384-96631800	DLX6-AS1
cg10659886	chr19:58629310-58630241	ZSCAN18
cg10695848	chr12:48206722-48207126	HDAC7
cg10700424	chr11:134201785-134202407	GLB1L2
cg10703826	chr1:119531992-119532196	TBX15
cg10715223	chr8:101661672-101662022	SNX31
cg10715425	chr1:6051596-6052955	NPHP4; KCNAB2
cg10716835	chr10:94833273-94835256	CYP26A1
cg10717463	chr18:76737006-76741244	SALL3
cg10723962	chr6:26240698-26240951	H4C6; HIST1H4F
cg10732215	chr10:22623351-22625875	
cg10741025	chr14:37131182-37132785	PAX9
cg10741422	chr19:21769190-21769786	
cg10755058	chr3:40428652-40429015	ENTPD3
cg10776919	chr1:50884229-50891471	DMRTA2
cg10783469	chr19:52390842-52391368	ZNF577; ZNF649-AS1
cg10795666	chr13:49794126-49795542	MLNR

cg10800464	chr12:109162409-109162722	
cg10801328	chr17:1106813-1107379	ABR
cg10802132	chr5:178487147-178487921	ZNF354C
cg10804656	chr10:22623351-22625875	
cg10808783	chr19:1465207-1471241	APC2
cg10823473	chr15:76630030-76630970	ISL2
cg10828284	chr22:50528202-50529085	MOV10L1
cg10859133	chr2:63281035-63281347	OTX1
cg10869581	chrX:102000690-102001558	BHLHB9; ARMCX5-GPRASP2
cg10879116	chr1:85358360-85359187	LPAR3
cg10882304	chr10:123922851-123923542	TACC2
cg10886442	chr3:142837886-142840838	CHST2
cg10890302	chr6:32063534-32065044	TNXB
cg10900437	chr6:149068180-149069775	UST
cg10900455	chr20:42543098-42545137	TOX2
cg10903903	chr6:27647794-27648005	
cg10904986	chr8:145555343-145562310	SCRT1
cg10922935	chr17:78449508-78452783	NPTX1
cg10932018	chr5:54516268-54516919	MCIDAS
cg10941566	chr22:30116082-30117160	CABP7
cg10959198	chr15:93631429-93633173	RGMA
cg10961604	chr1:27709601-27709831	CD164L2
cg10964367	chr8:1771362-1772760	ARHGEF10
cg10979880	chr6:105584149-105585621	BVES; BVES-AS1
cg10981464	chr1:156828805-156831102	NTRK1; INSRR
cg10982364	chr2:200320503-200329681	SATB2
cg11002404	chr10:11059443-11060524	CELF2
cg11012584	chr2:242549374-242549995	THAP4
cg11013726	chr15:65116014-65116567	PIF1
cg11015251	chr7:27169573-27170638	HOXA3; HOXA4; HOXA4-AS2
cg11026555	chr12:124246525-124247254	DNAH10
cg11042561	chr9:99449111-99449578	
cg11051055	chr8:11057685-11058479	XKR6
cg11068343	chr6:24360030-24360411	DCDC2
cg11068946	chr10:134597358-134602649	NKX6-2
cg11070069	chr6:105388191-105389545	LIN28B; LIN28B-AS1
cg11073571	chr4:78978366-78979256	FRAS1
cg11077516	chr13:100620242-100624348	ZIC5
cg11098493	chr3:49314438-49314815	USP4
cg11102782	chr19:18543829-18549161	ISYNA1; SSBP4
cg11134430	chr3:193987426-193987758	
cg11139646	chr1:40253684-40255172	BMP8B
cg11144753	chr14:103739579-103740892	
cg11172693	chr20:37434207-37435592	PPP1R16B
cg11173146	chr13:107186469-107189024	EFNB2
cg11174855	chr10:134597358-134602649	NKX6-2
cg11176990	chr2:39186778-39187968	ARHGEF33
cg11183365	chr6:27064629-27065004	
cg11184748	chr3:36805950-36806214	DCLK3
cg11198128	chr11:65601070-65601551	SNX32

cg11207307	chr12:8025365-8025908	SLC2A14
cg11213690	chr7:149112307-149112545	
cg11214757	chr5:176023778-176024651	GPRIN1
cg11219917	chr4:818647-820970	CPLX1
cg11223367	chr3:101497831-101498648	NXPE3
cg11229513	chr8:70981874-70984888	PRDM14
cg11229862	chr3:126113548-126113967	CFAP100; CCDC37-DT
cg11235741	chr15:82335473-82339937	MEX3B
cg11254700	chr19:53561216-53561812	
cg11258043	chr6:73972820-73973027	KHDC1
cg11260097	chr7:121940007-121940648	FEZF1
cg11267955	chr5:101632050-101632373	SLCO4C1
cg11279444	chr1:53098630-53099303	SHISAL2A
cg11281641	chr2:171672311-171675447	GAD1
cg11282657	chr17:79614851-79615559	TSPAN10
cg11294513	chr19:58220190-58220517	ZNF154
cg11296759	chr12:7592407-7593030	CD163L1
cg11299854	chr5:132082874-132083911	CCNI2
cg11312408	chr13:58206527-58208930	PCDH17
cg11325578	chrX:9733155-9734064	GPR143
cg11326255	chr19:3785512-3786653	MATK
cg11331678	chr20:44540446-44540957	PLTP
cg11342452	chr10:134597358-134602649	NKX6-2
cg11343211	chr17:15686219-15686474	IL6STP1
cg11361344	chr2:220361463-220363254	SPEGNB
cg11364273	chr11:63766059-63768666	MACROD1
cg11364468	chr7:100806280-100809064	VGF
cg11376305	chr19:49127389-49127720	SPHK2
cg11377136	chr22:46658398-46659332	PKDREJ
cg11377730	chr8:17270604-17271120	MTMR7
cg11398523	chr19:50316212-50316469	FUZ
cg11410023	chr3:96532024-96533625	EPHA6
cg11413039	chr11:125774293-125774584	DDX25
cg11431957	chr3:38035702-38036000	VILL
cg11439596	chr5:1930755-1931986	AC124852
cg11456756	chr6:30227321-30228255	HLA-L; HCG17
cg11459603	chr6:26273291-26273557	HIST1H2BI; H2BC10
cg11472279	chr10:11059443-11060524	CELF2
cg11474250	chr5:107005131-107008191	EFNA5
cg11480800	chr15:55879793-55881228	PYGO1
cg11481534	chr6:116691828-116692868	DSE
cg11485595	chr5:32709911-32714450	NPR3
cg11500797	chr7:96651964-96652246	DLX5
cg11502745	chr11:18742691-18743513	IGSF22
cg11509907	chr2:235404503-235406541	ARL4C
cg11510060	chr4:330163-332068	ZNF141
cg11510523	chr19:12305553-12306304	
cg11521404	chr13:20735044-20736157	GJA3
cg11525280	chr2:26624604-26625057	DRC1
cg11531021	chr2:220416864-220417869	OBSL1

cg11536474	chr2:63285950-63287097	
cg11549953	chr5:43017969-43018668	
cg11563680	chr6:100917206-100917523	
cg11569407	chr10:130008559-130009620	
cg11576274	chr1:146552329-146552577	
cg11592503	chr7:145813031-145814084	CNTNAP2
cg11594731	chr3:46940055-46940394	PTH1R
cg11595545	chr1:111216245-111217937	KCNA3
cg11597475	chr4:57975861-57976916	IGFBP7; IGFBP7-AS1
cg11600807	chr11:17373020-17373665	NCR3LG1
cg11621464	chr19:41119032-41120394	LTBP4
cg11629889	chr17:40838850-40839062	CNTNAP1
cg11630554	chr4:165878037-165878446	FAM218A
cg11660826	chr6:150284683-150286515	ULBP1
cg11664500	chr6:133562087-133563586	EYA4
cg11667020	chr20:21376359-21378245	NKX2-4
cg11668749	chr1:229567207-229570684	ACTA1
cg11671688	chr6:110299366-110301267	GPR6
cg11672054	chr1:228645197-228646434	HIST3H2A; HIST3H2BB
cg11673291	chr1:36786501-36789402	EVA1B; SH3D21
cg11684022	chr1:247494250-247495840	ZNF496
cg11686528	chr17:1082377-1083833	ABR
cg11691298	chr2:26396103-26397204	GAREM2
cg11694752	chr11:75378417-75379882	MAP6
cg11697427	chr10:102590123-102590402	
cg11699265	chr7:98990158-98990922	ARPC1B
cg11701471	chr8:54163304-54164443	OPRK1
cg11716665	chr5:149569593-149569945	SLC6A7
cg11721803	chr12:48397890-48398731	COL2A1
cg11723850	chr14:24020003-24020251	ZFHX2
cg11724135	chr20:25128765-25129610	
cg11726648	chr2:19560964-19561650	
cg11737742	chr1:110752257-110754794	KCNC4
cg11753158	chr10:22540708-22542739	
cg11756095	chr1:32410189-32410630	
cg11763509	chr1:39956425-39958137	BMP8A
cg11804775	chr19:19729128-19729814	PBX4
cg11806178	chr20:39316551-39319987	MAFB
cg11823511	chr1:91183241-91184540	BARHL2
cg11827910	chr11:31825744-31826967	PAX6
cg11849086	chr17:62774367-62777797	27P1-BPTFP1-KPNA2P3; ARH
cg11855325	chr7:30028519-30029822	SCRN1
cg11856897	chr1:47998900-47999517	
cg11857704	chr2:3750829-3751927	DCDC2C
cg11869770	chr19:34972055-34973645	WTIP
cg11880855	chr10:16561605-16563822	C1QL3
cg11889769	chr8:104383410-104384109	CTHRC1
cg11891395	chr7:96651964-96652246	DLX5
cg11891579	chr5:158478379-158478630	EBF1
cg11915388	chr22:42470036-42470669	PHETA2

cg11929643	chr3:48698336-48701667	CELSR3
cg11940285	chr3:129693128-129694841	TRH
cg11942956	chr6:133562087-133563586	EYA4
cg11945824	chr6:32116591-32117229	PRRT1
cg11956953	chr17:27346854-27347222	
cg11957331	chr19:42827744-42829149	TMEM145
cg11973177	chr10:17270431-17272617	VIM; VIM-AS1
cg11979589	chr4:110222971-110224257	COL25A1
cg11996428	chr9:135464587-135466240	BARHL1
cg12002303	chr15:68113242-68113520	SKOR1
cg12002882	chr20:62461267-62462037	ZBTB46
cg12004787	chr11:69517841-69519929	FGF19
cg12007166	chr6:30227321-30228255	HLA-L; HCG17
cg12018098	chr19:38885234-38885505	SPRED3
cg12022772	chr2:131129719-131130511	PTPN18
cg12028548	chr20:590223-591222	TCF15
cg12035092	chr2:149632683-149633882	KIF5C
cg12040486	chr19:11959578-11960064	ZNF439
cg12041056	chr22:27053082-27053629	MIAT
cg12043818	chr10:47008085-47008410	GPRIN2
cg12053155	chr13:107186469-107189024	EFNB2
cg12058947	chr15:83875649-83877079	HDGFL3
cg12060499	chr14:102172004-102172705	
cg12060744	chr19:58094740-58095764	ZIK1
cg12068816	chr22:40390485-40391490	FAM83F
cg12078320	chr1:54940347-54940753	
cg12078929	chr22:42896637-42897041	SERHL
cg12086936	chr11:31831621-31839038	PAX6
cg12091732	chr10:82116203-82117120	DYDC2
cg12098872	chr19:47742726-47743151	
cg12099952	chr20:3052098-3053103	OXT
cg12111714	chr13:26042694-26043486	ATP8A2
cg12115991	chr6:108437999-108441208	
cg12117141	chr8:11557853-11558252	GATA4
cg12121643	chr3:185911345-185912228	DGKG
cg12127472	chr17:42733487-42734626	MEIOC
cg12142445	chr19:53970803-53971473	ZNF813
cg12148647	chr19:3687787-3688262	PIP5K1C
cg12162377	chr2:232260101-232261134	B3GNT7
cg12169233	chr19:11959578-11960064	ZNF439
cg12170314	chr11:15136059-15136545	INSC
cg12174341	chr8:41424342-41425300	
cg12175729	chr8:140714586-140718259	KCNK9
cg12177220	chr3:38080629-38081187	DLEC1
cg12178578	chr1:45792420-45793301	HPDL
cg12188986	chr11:93063599-93064076	DEUP1
cg12191938	chr10:88730555-88731632	AGAP11; ADIRF
cg12193731	chr12:52300802-52301583	ACVRL1
cg12204732	chr10:128993506-128995167	DOCK1
cg12206199	chr2:39186778-39187968	ARHGEF33

cg12212311	chr18:77557781-77558948	
cg12212657	chr3:3840514-3842772	LRRN1
cg12213062	chr2:220299484-220300243	SPEG
cg12215739	chr2:177014949-177015214	MIR10B; HOXD4
cg12217400	chr6:29521111-29521833	OR2I1P
cg12220691	chr19:41641308-41642082	
cg12229632	chr17:2607736-2608005	CLUH
cg12230728	chr12:58021295-58022037	B4GALNT1
cg12233363	chr6:100912072-100913337	SIM1
cg12233379	chr1:151812254-151812525	C2CD4D
cg12248614	chr19:41018526-41019486	SPTBN4
cg12269002	chr11:77122737-77123088	PAK1
cg12271981	chr13:88326601-88326919	SLITRK5
cg12272837	chr19:290595-292249	PLPP2
cg12281657	chr14:65016517-65016909	PPP1R36
cg12298268	chr3:139257713-139257949	RBP1
cg12306414	chr4:13548571-13549956	
cg12307840	chr1:223536843-223538628	SUSD4
cg12315713	chr11:93063599-93064076	DEUP1
cg12318501	chr22:50919454-50920376	ADM2
cg12319602	chr5:43017969-43018668	
cg12323723	chrX:136655910-136657085	ZIC3
cg12338417	chr3:32858195-32860506	TRIM71
cg12346592	chr13:20138856-20139323	
cg12356890	chr10:11059443-11060524	CELF2
cg12377139	chr10:22634001-22634862	SPAG6
cg12414557	chr4:1107126-1107728	RNF212
cg12414653	chr12:8123347-8123561	
cg12439325	chr12:51236634-51236924	TMPRSS12
cg12441126	chr7:751713-752150	PRKAR1B
cg12448161	chr3:48631883-48632901	COL7A1
cg12452512	chrX:100740222-100740601	ARMCX4
cg12456714	chr5:16179065-16180420	MARCH11
cg12457909	chr7:39649254-39649510	YAE1
cg12477533	chr2:98703355-98703889	VWA3B
cg12497914	chr20:2672905-2674698	EBF4
cg12508343	chr3:125075916-125076481	ZNF148
cg12508451	chr15:37172474-37173018	
cg12518410	chr2:109744586-109746833	SH3RF3; SH3RF3-AS1
cg12534216	chr6:30095174-30095610	
cg12539796	chr5:7849946-7850439	c5orf49
cg12547166	chr4:206378-206892	ZNF876P
cg12549600	chr1:113286333-113287172	
cg12552771	chr22:29709282-29712013	RASL10A
cg12558012	chr19:19280999-19281560	BORCS8-MEF2B; MEF2B
cg12590600	chr20:643976-645233	SCRT2
cg12595013	chr3:147126989-147128999	ZIC1
cg12597389	chr7:8481975-8482762	NXPH1
cg12598635	chr4:818647-820970	CPLX1
cg12604950	chr1:180881317-180882592	KIAA1614

cg12610471	chr10:22634001-22634862	SPAG6
cg12615766	chr8:59058168-59059414	FAM110B
cg12629875	chr1:247170869-247171434	ZNF625; ZNF625-ZNF20
cg12630461	chr15:91499953-91500818	RCCD1
cg12630714	chr4:54975388-54976202	
cg12631351	chr5:80255905-80257006	RASGRF2; RASGRF2-AS1
cg12640000	chr1:62660556-62660946	L1TD1
cg12642693	chrX:50556225-50557179	SHROOM4
cg12646649	chr10:102984183-102990063	LBX1; LBX1-AS1
cg12648201	chr2:27664940-27665151	KRTCAP3; NRBP1
cg12659883	chr1:119543057-119543454	
cg12668309	chr3:53527928-53530106	CACNA1D
cg12673103	chr5:134363093-134365146	PITX1
cg12690148	chr1:63782395-63790471	FOXD3; FOXD3-AS1
cg12696328	chr1:227729516-227730492	
cg12710510	chr12:3862069-3862606	CRACR2A; EFCAB4B
cg12727374	chr3:183542497-183543804	MAP6D1
cg12727398	chr2:237086728-237088463	
cg12741994	chr3:170136243-170137886	CLDN11
cg12744820	chr6:137814356-137815202	OLIG3
cg12756396	chr1:50884229-50891471	DMRTA2
cg12758687	chr11:113345069-113346328	DRD2
cg12781700	chr17:42733487-42734626	MEIOC
cg12782180	chr7:127880751-127881375	LEP
cg12792509	chr17:27493204-27493880	MYO18A
cg12796229	chr18:12407716-12408242	SLMO1
cg12804791	chr11:126286452-126286870	
cg12810212	chr2:232378949-232379666	
cg12812233	chr4:40752692-40752896	NSUN7
cg12816961	chr10:134597358-134602649	NKX6-2
cg12820681	chr7:155164558-155167854	BLACE
cg12825804	chr14:77227570-77228691	VASH1
cg12828075	chr11:15136059-15136545	INSC
cg12840502	chr22:30116082-30117160	CABP7
cg12847554	chr6:100909211-100909444	SIM1
cg12855166	chr17:30845904-30846702	MYO1D
cg12863545	chr17:8868470-8869372	PIK3R5
cg12872693	chr3:179168736-179169593	GNB4
cg12877723	chr8:57358127-57359415	PENK
cg12883980	chr13:50707586-50708019	DLEU1
cg12892506	chr3:147113609-147114479	ZIC4
cg12927498	chr5:141030919-141031177	FCHSD1
cg12928379	chr11:636907-640628	DRD4
cg12929487	chr4:41869175-41869459	
cg12936335	chr6:150284683-150286515	ULBP1
cg12940439	chr1:67600391-67600715	C1orf141
cg12940822	chr5:127872564-127874945	FBN2
cg12945444	chr8:11536768-11538961	GATA4
cg12966367	chr3:50377804-50378540	RASSF1
cg12973941	chr4:13543563-13546494	NKX3-2

cg12974388	chr2:237071795-237078762	GBX2
cg12975230	chr18:73167403-73167920	
cg12978433	chr20:52789253-52790986	CYP24A1
cg12986271	chr19:31839636-31843049	TSHZ3
cg12992720	chr19:19738573-19739821	LAPAR2
cg12997720	chr7:27225051-27225629	HOXA11; HOXA11-AS
cg12998491	chr9:134151854-134153015	FAM78A
cg13001868	chr17:43339125-43339832	SPATA32; MAP3K14-AS1
cg13021333	chr6:6002472-6005125	NRN1
cg13024368	chr11:12029738-12030841	DKK3
cg13040693	chr13:45885755-45886103	
cg13045555	chr8:105342214-105342558	
cg13056495	chr7:134143116-134144063	AKR1B1
cg13066043	chr14:24808636-24808992	RIPK3
cg13072057	chr3:160822495-160823260	B3GALNT1
cg13080379	chr1:119526783-119527192	TBX15
cg13088368	chr20:42543098-42545137	TOX2
cg13092806	chr2:177042752-177043444	HAGLR; HAGLROS
cg13102585	chr17:42385811-42386393	RUNDC3A; RUNDC3A-AS1
cg13111733	chr1:228645197-228646434	HIST3H2BB; HIST3H2A
cg13118906	chr17:61523001-61524470	CYB561
cg13122377	chr19:57683597-57683850	DUXA
cg13139972	chr4:107956556-107957453	DKK2
cg13155001	chr4:121843149-121844193	PRDM5
cg13160874	chr2:242549374-242549995	THAP4
cg13167730	chr1:47909713-47911020	
cg13168407	chr22:38808785-38809017	
cg13176012	chr2:157176252-157177346	
cg13177786	chr11:10472001-10472857	AMPD3
cg13191049	chr15:99645031-99646444	SYNM
cg13198321	chr15:58357310-58358776	ALDH1A2
cg13204512	chr17:29298047-29298606	RNF135
cg13208922	chr4:298804-299312	ZNF732
cg13239420	chr1:240160838-240161616	
cg13246235	chr6:12749878-12750499	PHACTR1
cg13247663	chr8:59058168-59059414	FAM110B
cg13258989	chr17:72968168-72969164	HID1
cg13267264	chr8:70981874-70984888	PRDM14
cg13267747	chr5:141030919-141031177	FCHSD1
cg13274077	chr2:121334309-121334801	
cg13280283	chr6:137242316-137245442	SLC35D3
cg13283952	chr2:121411984-121412315	
cg13298199	chr1:208132328-208133117	
cg13300273	chr1:200842197-200843388	GPR25
cg13304665	chr22:22862624-22863220	ZNF280B
cg13314145	chr7:98245806-98247759	NPTX2
cg13323701	chr6:118228043-118229811	SLC35F1
cg13325154	chr10:104000255-104001741	PITX3
cg13326227	chr20:20344401-20350605	INSM1
cg13327545	chr10:22623351-22625875	

cg13335974	chr17:72838813-72839751	GRIN2C
cg13337047	chr19:57683597-57683850	DUXA
cg13341720	chr19:6740670-6741203	TRIP10
cg13342435	chr11:30605559-30605800	MPPED2; MPPED2-AS1
cg13352836	chr16:31225957-31228264	TRIM72
cg13355047	chr19:4304543-4305122	FSD1
cg13356896	chr2:198649752-198651599	BOLL
cg13368923	chr6:1378446-1379318	
cg13372458	chr12:95266833-95267785	
cg13389502	chr17:1952920-1962328	HIC1
cg13409645	chr19:55865825-55866324	COX6B2; FAM71E2
cg13422850	chr9:135116148-135117929	NTNG2
cg13423383	chr9:139888935-139890343	CLIC3
cg13424029	chr10:101297439-101297703	NKX2-3
cg13438961	chr1:41249091-41250161	KCNQ4
cg13443627	chr1:59042014-59043295	TACSTD2
cg13445593	chr6:30227321-30228255	HLA-L; HCG17
cg13445796	chr11:20181201-20182325	DBX1
cg13459498	chr20:42543098-42545137	TOX2
cg13462028	chr18:9707753-9709311	RAB31
cg13465477	chr9:133534535-133542394	PRDM12
cg13468002	chr3:49906738-49907482	CAMKV
cg13472192	chr1:41349741-41350293	
cg13476854	chr1:119549145-119551320	
cg13481969	chr9:99449111-99449578	
cg13484549	chr19:49646093-49646308	PPFIA3
cg13490403	chr9:124981536-124982835	LHX6
cg13491250	chr2:20068429-20068907	LINC00954
cg13499300	chr19:54369388-54369809	MYADM
cg13516820	chr9:37034136-37038341	PAX5
cg13525067	chr19:15662114-15662358	CYP4F22
cg13530938	chr2:239140097-239140659	
cg13532885	chrX:47478671-47479515	SYN1
cg13545212	chr17:6616423-6617471	SLC13A5
cg13556491	chr19:19738573-19739821	LAPAR2
cg13562542	chr3:71802561-71804209	EIF4E3
cg13564825	chr19:38746639-38747379	PPP1R14A
cg13570982	chr14:55595698-55596692	LGALS3
cg13592399	chr14:52534582-52536722	NID2
cg13601435	chr9:120507228-120507642	
cg13607709	chr3:46940055-46940394	PTH1R
cg13610307	chr9:140917285-140918052	CACNA1B
cg13628577	chr5:122430677-122431443	PRDM6
cg13634242	chr5:60921535-60922472	c5orf64
cg13634602	chr18:76737006-76741244	SALL3
cg13641185	chr6:29521111-29521833	OR2I1P
cg13647155	chr5:176046144-176047641	SNCB
cg13647536	chr7:27260102-27260467	
cg13662093	chr20:33865334-33865563	MP24OS; MMP24-AS1-EDEM
cg13677149	chr7:27284640-27286237	EVX1

cg13687570	chr19:37825102-37825756	HKR1
cg13692446	chr13:112758599-112760491	SOX1-OT
cg13699414	chr5:178957434-178957900	
cg13702005	chr12:103889149-103889843	C12orf42
cg13703049	chr7:27212417-27214396	HOXA10; HOXA10-HOXA9
cg13703871	chr19:9473590-9474001	ZNF559-ZNF177
cg13707894	chr3:27674210-27674639	
cg13717446	chr17:62774367-62777797	27P1-BPTFP1-KPNA2P3; ARH
cg13721404	chr18:31739048-31739522	NOL4
cg13725782	chr6:105584149-105585621	BVES; BVES-AS1
cg13726682	chr17:29718231-29719291	RAB11FIP4
cg13729891	chr17:7108305-7108654	DLG4
cg13730743	chr19:52207133-52207731	SPACA6
cg13755546	chr2:74425445-74426423	MTHFD2
cg13759674	chr9:140051063-140051730	GRIN1
cg13765939	chr22:27053082-27053629	MIAT
cg13768269	chr9:112917-114839	
cg13784235	chr6:108495655-108495986	NR2E1
cg13788515	chr2:207139337-207140031	ZDBF2
cg13791254	chr9:100615235-100617510	FOXE1
cg13794530	chr7:158936508-158938492	VIPR2
cg13794993	chr18:76737006-76741244	SALL3
cg13796381	chr9:139872238-139873143	PTGDS
cg13796823	chr6:130686415-130687736	SAMD3; TMEM200A
cg13798146	chr15:83875649-83877079	HDGFL3
cg13800209	chr15:37390176-37390380	MEIS2
cg13802013	chr20:37302814-37303388	
cg13803688	chr1:110610266-110613303	ALX3
cg13817952	chr9:124987744-124991086	LHX6
cg13846270	chr13:51417372-51418149	DLEU7; DLEU7-AS1
cg13849378	chr1:241520104-241520790	RGS7
cg13850380	chr1:1474963-1475220	TMEM240
cg13850606	chr1:229542944-229543770	
cg13865488	chr9:137299191-137299437	RXRA
cg13868084	chr9:86755386-86755700	
cg13877670	chr15:53080459-53083699	ONECUT1
cg13879483	chr12:95941907-95942979	USP44
cg13887205	chr5:139525538-139526227	
cg13888593	chr2:74641734-74643350	C2orf81
cg13894813	chr11:68181087-68181289	LRP5
cg13900100	chr6:43970074-43970922	c6orf223
cg13902210	chr1:110752257-110754794	KCNC4
cg13912311	chr9:127265171-127267659	NR5A1
cg13913015	chr2:47796924-47799166	KCNK12
cg13917662	chr13:100547634-100548911	CLYBL
cg13921956	chr2:12856626-12859019	TRIB2
cg13930892	chr11:2290105-2292932	ASCL2
cg13933080	chr10:22634001-22634862	SPAG6
cg13933262	chr5:140797163-140797701	2,3,4,5,6,7,8,9,10; PCDHGB1,
cg13933734	chr1:119549145-119551320	

cg13942157	chr15:29862036-29863649	FAM189A1
cg13947995	chr15:91427541-91427905	FES
cg13952656	chr2:232260101-232261134	B3GNT7
cg13954457	chr5:167956220-167957371	FBLL1
cg13967888	chr9:97094627-97095058	
cg13974632	chr11:27740645-27741236	BDNF
cg13974773	chr6:27463177-27463379	
cg13984181	chr1:92495368-92495964	EPHX4
cg13990746	chr1:173638663-173639045	ANKRD45
cg13996426	chr3:49755764-49757317	AMIGO3; GMPPB; RNF123
cg13997645	chr11:15136059-15136545	INSC
cg14002345	chr11:31831621-31839038	PAX6
cg14002960	chr18:31739048-31739522	NOL4
cg14007036	chrX:136113584-136114232	GPR101
cg14007067	chr18:76737006-76741244	SALL3
cg14013195	chr2:26624604-26625057	DRC1
cg14015044	chr8:22960385-22960927	TNFRSF10C
cg14019323	chr12:65218246-65219143	TBC1D30
cg14024461	chr1:228645197-228646434	HIST3H2BB; HIST3H2A
cg14038391	chr10:102899823-102900263	TLX1NB
cg14044640	chr7:27186928-27187692	HOXA6; HOXA-AS3
cg14045872	chr7:49813009-49815752	VWC2
cg14052210	chr6:26521948-26522579	HCG11
cg14054357	chr17:2607736-2608005	CLUH
cg14065127	chr20:3063006-3064146	AVP
cg14069049	chr4:11430236-11431256	HS3ST1
cg14069965	chr6:24357720-24358309	DCDC2; KAAG1
cg14079463	chr6:127796287-127797356	SOGA3
cg14084907	chr1:3634634-3635101	TP73
cg14088357	chr19:46800054-46800603	HIF3A
cg14092045	chr9:116450146-116450454	
cg14095438	chr8:49468684-49468959	
cg14095692	chr18:8704722-8707557	GACAT2; MTCL1
cg14098681	chr10:8091375-8098329	GATA3; GATA3-AS1
cg14104369	chr20:57463653-57467739	GNAS
cg14117138	chr19:46800054-46800603	HIF3A
cg14126493	chr5:176830276-176831639	F12
cg14132888	chr5:114514717-114516220	TRIM36
cg14142171	chr6:30227321-30228255	HLA-L; HCG17
cg14144513	chr11:69258150-69258544	
cg14145477	chr13:33589929-33591428	KL
cg14153126	chr9:131218101-131219409	ODF2
cg14156405	chr1:241520104-241520790	RGS7
cg14159026	chr6:105584149-105585621	BVES; BVES-AS1
cg14159336	chr1:228645197-228646434	HIST3H2BB; HIST3H2A
cg14159790	chr9:139886313-139887180	PAXX
cg14166009	chr19:37825102-37825756	HKR1
cg14169333	chr9:96720587-96723189	BARX1-DT
cg14178748	chr1:153650831-153652375	NPR1
cg14183206	chr6:30227321-30228255	HLA-L; HCG17

cg14185918	chr9:110249749-110252660	KLF4
cg14186641	chr6:88875398-88877449	CNR1
cg14186816	chr18:55862654-55862873	NEDD4L
cg14189141	chr9:1042418-1042973	
cg14202325	chr3:13114628-13115245	IQSEC1
cg14216029	chr19:42901017-42901375	LIPE-AS1
cg14231297	chr19:58629310-58630241	ZSCAN18
cg14249348	chr4:48492118-48493589	ZAR1
cg14250130	chr10:3108651-3111419	PFKP
cg14251622	chr14:36002599-36005013	INSM2
cg14252279	chr9:124360439-124362382	DAB2IP
cg14252602	chr19:22018595-22018827	ZNF43
cg14263942	chr4:76555367-76556079	CDKL2
cg14270220	chr12:49371691-49375550	WNT1
cg14270292	chr6:133562087-133563586	EYA4
cg14273450	chr2:26624604-26625057	DRC1
cg14278853	chr6:29521111-29521833	OR2I1P
cg14284952	chr8:104152727-104153390	BAALC
cg14287112	chr6:133562087-133563586	EYA4
cg14287235	chr14:24803679-24804353	ADCY4
cg14298200	chr8:65498604-65498828	
cg14305313	chr9:109622425-109623203	
cg14306734	chr18:34833555-34834606	CELF4
cg14306956	chr9:37026223-37028014	PAX5
cg14314653	chr6:105584149-105585621	BVES; BVES-AS1
cg14314744	chr6:100897081-100897621	SIM1
cg14315558	chr21:26934424-26934805	MIR155HG
cg14320054	chr9:110227968-110228656	
cg14327531	chr10:8091375-8098329	GATA3; GATA3-AS1
cg14328457	chr17:37380954-37382363	STAC2
cg14337339	chr3:53527928-53530106	CACNA1D
cg14345012	chr20:26188639-26190348	MIR663AHG; MIR6633
cg14347199	chr9:91149946-91150648	NXNL2
cg14353137	chr8:67873389-67875600	TCF24
cg14361409	chr14:104583037-104584574	MIR203B
cg14362312	chr9:136293567-136294160	ADAMTS13
cg14369648	chr9:33750520-33751160	PRSS3
cg14380270	chr17:33700488-33700760	SLFN11
cg14382750	chr9:100263676-100265217	TMOD1
cg14396421	chr19:21769190-21769786	
cg14409810	chr9:140172614-140176105	TOR4A
cg14414971	chr7:3340355-3341216	SDK1
cg14416311	chr9:89560585-89562647	GAS1; GAS1RR
cg14416371	chr11:43602546-43603215	MIR129-2
cg14424538	chr20:1783825-1784552	
cg14451382	chr5:1874908-1879032	IRX4
cg14453201	chr5:320789-323010	AHRR
cg14456683	chr3:147126989-147128999	ZIC1
cg14458619	chr9:8857486-8858708	PTPRD; PTPRD-AS1
cg14463412	chr9:96588599-96588933	

cg14473102	chr2:176993480-176995557	HOXD8; HOXD-AS2
cg14473344	chr3:142837886-142840838	CHST2
cg14486338	chr8:99438693-99440425	KCNS2
cg14493920	chr9:37034136-37038341	PAX5
cg14498666	chr9:21031514-21031823	HACD4
cg14502484	chr5:1444679-1446648	SLC6A3
cg14506196	chr10:101294444-101297263	NKX2-3
cg14507560	chr5:1881925-1887743	IRX4; CTD2194D22.4
cg14510333	chr16:51168267-51169110	
cg14531663	chr6:32116591-32117229	PRRT1
cg14531862	chr19:44763979-44764312	ZNF233
cg14543941	chr10:71077947-71079377	HK1
cg14547067	chr11:46316876-46317485	CREB3L1
cg14556683	chr19:15342627-15343181	EPHX3
cg14557534	chr3:27762352-27763995	EOMES
cg14568830	chr10:23462225-23463889	
cg14570307	chr7:148982080-148982675	LOC155060; ZNF783
cg14572967	chr9:35616981-35617324	CD72
cg14587524	chr19:38182794-38183327	ZNF781
cg14592933	chr7:26415747-26416891	
cg14602438	chr19:37463992-37464606	ZNF568; ZNF829
cg14608384	chr5:54516268-54516919	MCIDAS
cg14614314	chr10:135148197-135150565	CALY
cg14614811	chr5:159399005-159399928	ADRA1B
cg14615784	chr5:94955631-94957244	GPR150
cg14619259	chr5:141705392-141705688	SPRY4-AS1
cg14631834	chr18:28621490-28623117	DSC3
cg14643892	chr12:64784011-64784664	C12orf56
cg14644001	chr6:32116591-32117229	PRRT1
cg14644871	chr7:26191795-26192757	NFE2L3
cg14645545	chr2:219252422-219252631	SLC11A1
cg14654324	chr9:137299191-137299437	RXRA
cg14654468	chr17:80186136-80189990	SLC16A3
cg14663510	chr10:124894076-124897366	HMX3
cg14670974	chr5:157098264-157099041	SOX30
cg14673387	chr10:47008085-47008410	GPRIN2
cg14673618	chr2:45169506-45171884	SIX3
cg14676825	chr17:27038085-27038919	PROCA1
cg14681055	chr10:104000255-104001741	PITX3
cg14688104	chr8:99438693-99440425	KCNS2
cg14696396	chr15:83775862-83776922	TM6SF1
cg14699728	chr11:66188131-66189397	NPAS4
cg14704921	chr4:53728038-53729000	RASL11B
cg14709460	chr7:28995306-28998541	TRIL
cg14709691	chr1:46632690-46632932	PIK3R3
cg14711690	chr1:226924561-226926553	ITPKB
cg14719951	chr18:28621490-28623117	DSC3
cg14732324	chr5:528384-528982	
cg14736058	chr4:16084196-16085735	PROM1
cg14738806	chr12:115102688-115103982	

cg14741939	chr3:147126989-147128999	ZIC1
cg14743594	chr5:167956220-167957371	FBLL1
cg14757228	chr6:32116591-32117229	PRRT1
cg14776962	chr6:26251899-26252233	HIST1H2BH; H2BC9
cg14780416	chr7:27208872-27209616	HOXA10-AS
cg14780632	chr11:65816405-65816665	GAL3ST3
cg14789818	chr1:227748424-227748860	
cg14808890	chr6:26250437-26250827	HIST1H3F
cg14819504	chr2:160760605-160761452	LY75; LY75-CD302
cg14822490	chr10:124907284-124911035	HMX2
cg14823851	chr17:59531724-59535254	TBX4
cg14826425	chr7:27231806-27233097	
cg14861089	chr10:102893661-102895059	TLX1
cg14871601	chr7:64029903-64030972	
cg14889643	chr18:60985504-60985741	BCL2
cg14897419	chr10:134120448-134122422	STK32C
cg14899716	chr1:42845979-42846988	RIMKLA
cg14907769	chr8:49468684-49468959	
cg14930075	chr8:140714586-140718259	KCNK9
cg14944647	chr11:43602546-43603215	MIR129-2
cg14986699	chr2:149632683-149633882	KIF5C
cg14988503	chr4:76555367-76556079	CDKL2
cg14991984	chr1:99469933-99470968	PLPPR5
cg14993712	chr7:138720045-138721019	ZC3HAV1L
cg14996143	chr2:172944989-172945384	METAP1D
cg14996783	chr7:28995306-28998541	TRIL
cg14999001	chr7:45197182-45197807	RAMP3
cg15000841	chr11:64815041-64815722	NAALADL1
cg15006175	chr8:145697880-145698784	KIFC2
cg15014975	chr1:25255528-25259005	RUNX3
cg15015920	chr5:16179065-16180420	MARCH11
cg15017004	chr18:2905951-2907348	EMILIN2
cg15032314	chr19:50096610-50096912	PRR12
cg15044248	chr17:75368689-75370506	SEPTIN9
cg15071854	chr1:45792420-45793301	HPDL
cg15073853	chr19:18543829-18549161	ISYNA1; SSBP4
cg15090005	chr1:10057122-10058108	RBP7
cg15092343	chr4:4859633-4860191	MSX1
cg15100599	chr1:223536843-223538628	SUSD4
cg15104644	chr1:231296560-231297345	TRIM67
cg15105326	chr3:147108512-147111703	ZIC4
cg15110403	chr19:17392384-17393775	ANKLE1
cg15114474	chr11:69451137-69458596	CCND1
cg15126179	chr13:79181328-79181614	OBI1-AS1
cg15126975	chr2:175208416-175209076	
cg15140191	chr6:56818874-56820308	DST; BEND6
cg15140703	chr7:99774734-99775583	STAG3
cg15147516	chr1:226411008-226411880	MIXL1
cg15148683	chr19:36736022-36736884	ZNF565
cg15149655	chr2:98703355-98703889	VWA3B

cg15157455	chr1:149399899-149400518	H3-2; HIST2H3PS2
cg15167155	chr19:53662190-53662500	ZNF347
cg15167646	chr17:47653212-47654369	NXPH3
cg15174623	chr7:35494214-35494702	
cg15192750	chr16:69999038-69999777	
cg15201417	chr11:66034752-66035054	KLC2; RAB1B
cg15201635	chr16:68480865-68482822	SMPD3
cg15202627	chr4:1685406-1686743	FAM53A
cg15203566	chr5:115697135-115697589	
cg15205527	chr13:45885755-45886103	
cg15205636	chr13:21520329-21520564	
cg15244223	chr6:118228043-118229811	SLC35F1
cg15264710	chr6:33396051-33396296	SYNGAP1
cg15267232	chr10:8091375-8098329	GATA3; GATA3-AS1
cg15297724	chr10:75407414-75407706	SYNPO2L
cg15317815	chr3:8808962-8811280	OXTR
cg15336765	chr12:50354840-50356163	AQP5
cg15339231	chr7:96650222-96651551	DLX5
cg15346168	chr14:24835811-24836220	NFATC4
cg15364725	chr10:92616821-92618034	HTR7
cg15365320	chr11:17373020-17373665	NCR3LG1
cg15375239	chr19:38754847-38755699	SPINT2
cg15377283	chr8:55370171-55372525	SOX17
cg15386964	chr6:26250437-26250827	HIST1H3F
cg15391574	chr11:134201785-134202407	GLB1L2
cg15396686	chr10:124894076-124897366	HMX3
cg15397374	chr8:49647703-49647988	EFCAB1
cg15412498	chr5:71402878-71404868	MAP1B
cg15415545	chr16:30204696-30206485	DLA2B; SLX1A; SLX1A-SULT1A
cg15427886	chr8:55378929-55380186	
cg15428435	chr14:102247661-102248279	PPP2R5C
cg15443535	chr14:93153279-93154759	RIN3
cg15444947	chr7:28995306-28998541	TRIL
cg15448975	chr4:5894072-5895116	CRMP1
cg15449956	chr8:106330519-106332120	ZFPM2
cg15457058	chr1:47881897-47883065	FOXE3
cg15459780	chr12:104609398-104610172	TXNRD1
cg15460093	chr10:135089970-135090491	ADAM8
cg15460872	chr15:99190447-99194559	IGF1R; IRAIN
cg15466862	chr13:112720565-112723582	SOX1
cg15467646	chr1:22140892-22141407	LDLRAD2
cg15471815	chr1:228645197-228646434	HIST3H2BB; HIST3H2A
cg15475851	chr10:105036629-105038084	INA
cg15490715	chr6:29521111-29521833	OR2I1P
cg15491911	chr6:167764523-167765124	
cg15506157	chr7:139167481-139168712	KLRG2
cg15506609	chr7:27203916-27206462	HOXA9
cg15520279	chr2:176993480-176995557	HOXD8; HOXD-AS2
cg15531403	chr8:53851702-53854426	NPBWR1
cg15545772	chr1:179560752-179561596	TDRD5

cg15553598	chr5:175792356-175793505	ARL10
cg15555527	chr4:80885845-80886148	ANTXR2
cg15558882	chr10:134597358-134602649	NKX6-2
cg15565032	chr15:68870634-68871974	CORO2B
cg15579650	chrX:101906002-101907017	ARMCX5-GPRASP2; GPRASP1
cg15600488	chr8:17270604-17271120	MTMR7
cg15602740	chr3:120003884-120004426	GPR156
cg15607538	chr12:133484659-133485739	
cg15611336	chr15:75248278-75249922	RPP25
cg15613048	chr1:21043833-21044771	KIF17
cg15617814	chr11:131780329-131781532	NTM
cg15618978	chr3:160167185-160168200	I59; TRIM59-IFT80; RP11432
cg15627078	chr13:24152900-24154140	TNFRSF19
cg15642758	chr15:83875649-83877079	HDGFL3
cg15646817	chr5:80255905-80257006	RASGRF2; RASGRF2-AS1
cg15655500	chr18:12407716-12408242	SLMO1
cg15661311	chr11:504190-504659	RNH1
cg15666214	chr6:108437999-108441208	
cg15672768	chr5:140741175-140741738	PCDHGB1,2,3,4
cg15679813	chr22:45403037-45406372	PHF21B
cg15681358	chr10:99473521-99474664	MARVELD1
cg15684724	chr8:67873389-67875600	TCF24
cg15690342	chr5:72594148-72595808	
cg15701313	chr12:125534061-125534527	
cg15720669	chr3:138663719-138666346	FOXL2; FOXL2NB
cg15728256	chr1:40253684-40255172	BMP8B
cg15732502	chr10:93647053-93647393	
cg15732768	chr7:96650222-96651551	DLX5
cg15732851	chr1:203598472-203598853	ATP2B4
cg15737319	chr2:74425445-74426423	MTHFD2
cg15747595	chr8:98289605-98290404	TSPYL5
cg15760257	chr17:26698360-26699557	SARM1
cg15766075	chr2:241496577-241497600	ANKMY1
cg15766101	chr19:46518284-46520080	CCDC61
cg15769764	chr4:995483-997541	IDUA
cg15775138	chr7:127744195-127744554	
cg15778437	chr11:31839364-31839813	PAX6
cg15790820	chr11:2290105-2292932	ASCL2
cg15802091	chr14:65006875-65009197	HSPA2
cg15811515	chr16:31580560-31581023	YBX3P1
cg15818307	chr6:29521111-29521833	OR2I1P
cg15822765	chr6:28956107-28956578	HCG15
cg15834072	chr4:155410624-155413393	DCHS2
cg15835396	chr7:128809090-128809393	TSPAN33
cg15841063	chr1:63782395-63790471	FOXD3; FOXD3-AS1
cg15844835	chr5:141030919-141031177	FCHSD1
cg15845792	chr6:28175283-28175605	
cg15846316	chr4:57371583-57372022	ARL9
cg15861585	chr11:636907-640628	DRD4
cg15866977	chr17:79917287-79920022	NOTUM

cg15869463	chr17:62774367-62777797	BPTFP1-KPNA2P3; ARHGAP2[illegible]
cg15873149	chr3:147136904-147137328	ZIC1
cg15875437	chr4:2059929-2063181	NAT8L
cg15883716	chr1:173638663-173639045	ANKRD45
cg15887459	chr11:18230620-18230906	SLC25A51P4
cg15904523	chr19:44763979-44764312	ZNF233
cg15912800	chr7:27208872-27209616	HOXA10-AS
cg15936446	chr5:42951077-42952410	
cg15949044	chr5:140864528-140864748	[illegible],8,9,10,11,12; PCDHGB1,2,3
cg15954353	chr17:5403132-5404602	
cg15968604	chr5:157098264-157099041	SOX30
cg15969216	chr13:45149971-45152288	TSC22D1
cg15979173	chr1:91182098-91182364	BARHL2
cg15985184	chr19:51830065-51831145	IGLON5
cg15987885	chr1:183386001-183387664	NMNAT2
cg15990972	chr2:177012372-177012675	
cg15991309	chr17:80291021-80292394	SECTM1
cg15992535	chr5:139227606-139228279	NRG2
cg15992563	chr13:28491489-28492518	PLUT
cg15993383	chr3:123166219-123168567	ADCY5
cg15994026	chr4:15779999-15780729	CD38
cg15994467	chr12:52400468-52401696	GRASP
cg15998779	chr3:159756633-159756997	IL12A-AS1
cg16002818	chr2:198649752-198651599	BOLL
cg16008609	chr1:213123648-213125092	VASH2
cg16022575	chr1:47902794-47905518	FOXD2
cg16042259	chr6:100917206-100917523	
cg16043357	chr10:118895964-118898037	VAX1
cg16046465	chr15:44486742-44487860	FRMD5
cg16054907	chr22:50705407-50706553	MAPK11
cg16076328	chr2:47596630-47597256	EPCAM
cg16086620	chr14:93389246-93389899	CHGA
cg16097357	chr17:73583839-73586337	MYO15B
cg16104915	chr7:27203916-27206462	HOXA9
cg16108230	chr6:117584585-117584816	
cg16113681	chr22:19748651-19749189	TBX1
cg16115720	chr1:208084099-208084513	CD34
cg16124981	chr19:9896564-9896994	ZNF846
cg16126280	chr5:158527375-158527983	AK123543
cg16128701	chr8:82192379-82193685	FABP5
cg16132520	chr7:134143116-134144063	AKR1B1
cg16133088	chr12:3862069-3862606	CRACR2A; EFCAB4B
cg16145113	chr1:119549145-119551320	
cg16150752	chr5:16179065-16180420	MARCH11
cg16158807	chr9:95946148-95947835	WNK2
cg16194233	chr6:108437999-108441208	
cg16197925	chr7:121943868-121944538	FEZF1; FEZF1-AS1
cg16215203	chr14:24803679-24804353	ADCY4
cg16218721	chr2:177017267-177017489	HOXD4
cg16219246	chr4:57396749-57397269	THEGL

cg16269733	chr1:156616554-156616946	BCAN
cg16281276	chr11:7694712-7695685	CYB5R2
cg16288089	chr7:97361133-97363018	TAC1
cg16306115	chr6:24357720-24358309	DCDC2; KAAG1
cg16308533	chr17:40838850-40839062	CNTNAP1
cg16311536	chr10:105344174-105345039	NEURL1
cg16318112	chr1:967967-970238	AGRN
cg16328007	chr1:41119853-41120136	RIMS3
cg16332256	chr4:9533767-9534520	
cg16332936	chr22:19748651-19749189	TBX1
cg16334314	chr6:84417598-84419378	SNAP91
cg16339238	chr20:23028404-23032218	THBD
cg16345647	chr21:38377938-38379437	RIPPLY3
cg16348470	chr1:34642383-34643024	c1orf94
cg16358679	chr15:78556406-78557542	DNAJA4
cg16364121	chr17:35291900-35300875	LHX1; LHX1-DT
cg16367511	chr2:74425445-74426423	MTHFD2
cg16376108	chr16:54962423-54967805	IRX5; CRNDE
cg16390139	chr6:74161087-74162090	CGAS
cg16391955	chr1:50880917-50881516	
cg16399136	chr1:151103686-151106100	SEMA6C
cg16404157	chr14:38724255-38725537	CLEC14A
cg16407471	chr11:43602546-43603215	MIR129-2
cg16441259	chr8:11557853-11558252	GATA4
cg16460359	chr15:72611947-72612802	CELF6
cg16475755	chr14:77964572-77965670	ISM2
cg16476975	chr7:155164558-155167854	BLACE
cg16485975	chr20:25063839-25065525	VSX1
cg16512615	chr4:141489963-141490378	UCP1
cg16519587	chr6:26614014-26614851	
cg16520815	chr16:4732831-4733184	MGRN1
cg16523704	chr3:49314438-49314815	USP4
cg16528511	chr6:84562821-84563758	RIPPLY2
cg16551483	chr17:4981358-4981979	ZFP3
cg16553435	chr16:89267824-89268087	SLC22A31
cg16558432	chr17:5403132-5404602	
cg16561266	chr17:62774367-62777797	BPTFP1-KPNA2P3; ARHGAP2
cg16566400	chr11:119293321-119293943	THY1; USP2-AS1
cg16573782	chr16:49314038-49316543	CBLN1
cg16580499	chr18:76737006-76741244	SALL3
cg16615154	chr6:40567267-40567580	
cg16620382	chr6:43211115-43211345	TTNK1
cg16622920	chr6:28956107-28956578	HCG15
cg16624692	chr11:65601070-65601551	SNX32
cg16626067	chr16:66612750-66613412	KLF-CMTM1; CMTM1; CNMT
cg16626884	chr2:71693165-71694127	DYSF
cg16633750	chr7:2558372-2559967	LFNG
cg16642791	chr16:54962423-54967805	IRX5; CRNDE
cg16650717	chr1:19970256-19971923	NBL1; MICOS10-NBL1
cg16651126	chr7:27169573-27170638	HOXA3; HOXA4; HOXA4-AS2

cg16655791	chr6:106429112-106429772	
cg16655905	chr19:38754847-38755699	SPINT2
cg16657538	chr18:32847285-32848130	ZSCAN30
cg16676492	chr15:34806491-34807382	
cg16686137	chr19:48837142-48837549	TMEM143
cg16688483	chr8:102505513-102506430	GRHL2
cg16692735	chr10:135259493-135260192	
cg16692998	chr19:19738573-19739821	LAPAR2
cg16697214	chr7:50342896-50343456	IKZF1
cg16703135	chr7:55516872-55517249	
cg16703956	chr5:1444679-1446648	SLC6A3
cg16709232	chr17:72321949-72322461	KIF19
cg16732469	chr17:4981358-4981979	ZFP3
cg16734164	chr19:41316544-41317318	CYP2T1P
cg16741041	chr13:36919738-36921004	SPART; SPART-AS1
cg16755500	chr17:27918162-27918398	ABHD15-AS1
cg16775746	chr22:42353660-42353900	
cg16778903	chr15:72611947-72612802	CELF6
cg16779463	chr17:75368689-75370506	SEPTIN9
cg16781647	chr2:73151201-73152060	EMX1
cg16788286	chr8:142215692-142216299	
cg16807089	chr7:143582126-143582610	TCAF1
cg16820186	chr11:17373020-17373665	NCR3LG1
cg16823083	chr1:3566446-3569636	TP73; WRAP73
cg16829453	chr17:48636104-48639279	CACNA1G; CACNA1G-AS1
cg16832267	chr2:18059494-18060920	KCNS3
cg16844941	chr8:11555100-11555603	GATA4
cg16848524	chr8:145806259-145806713	ARHGAP39
cg16857858	chr7:27212417-27214396	HOXA10; HOXA10-HOXA9
cg16859924	chr17:20059029-20060060	SPECC1
cg16862295	chr4:42399153-42400802	SHISA3
cg16867584	chr19:49199965-49200184	FUT2
cg16871763	chr8:11536768-11538961	GATA4
cg16877924	chr2:73429284-73430647	NOTO
cg16882226	chr2:101033607-101034296	CHST10
cg16886987	chr21:38377938-38379437	RIPPLY3
cg16896847	chr8:144510851-144513957	MAFA
cg16909408	chr3:134125488-134125871	
cg16909733	chr2:241459633-241460047	ANKMY1
cg16915821	chr11:12029738-12030841	DKK3
cg16919569	chr4:174451829-174452962	HAND2-AS1
cg16928066	chr2:73143056-73148260	EMX1
cg16949120	chr10:134597358-134602649	NKX6-2
cg16964348	chr7:24323559-24325080	NPY
cg16966315	chr7:149112307-149112545	
cg16969368	chr17:57642721-57643294	DHX40
cg16969586	chr18:10726248-10726667	PIEZO2
cg16977570	chr13:24152900-24154140	TNFRSF19
cg16980360	chr17:29298047-29298606	RNF135
cg17003293	chr14:36002599-36005013	INSM2

cg17011276	chr5:140797163-140797701	2,3,4,5,6,7,8,9,10; PCDHGB1,
cg17019053	chr2:74875129-74875562	M1AP
cg17021218	chr10:88730555-88731632	AGAP11; ADIRF
cg17025835	chr7:123672064-123673691	TMEM229A
cg17030173	chr5:16179065-16180420	MARCH11
cg17034390	chr6:30095174-30095610	
cg17035412	chr7:26415747-26416891	
cg17039236	chr18:77547966-77549038	
cg17054969	chr12:48397890-48398731	COL2A1
cg17062279	chr17:3658427-3658685	ITGAE
cg17073392	chr15:69323960-69324232	NOX5
cg17078116	chr8:24770909-24772547	NEFM
cg17078427	chr3:170136243-170137886	CLDN11
cg17088631	chr1:53098630-53099303	SHISAL2A
cg17098147	chr10:22634001-22634862	SPAG6
cg17101296	chr8:145925411-145926101	
cg17104258	chr1:167090576-167091010	DUSP27
cg17105755	chr2:74875129-74875562	M1AP
cg17124224	chr15:83951981-83953930	BNC1
cg17137424	chr1:231297813-231299777	TRIM67
cg17147211	chr1:119543822-119544339	
cg17161250	chr2:106681983-106682403	ECRG4
cg17162576	chr1:66998729-66999060	SGIP1
cg17172980	chr3:196755678-196757374	MELTF
cg17176016	chr6:108437999-108441208	
cg17180977	chr1:228134423-228136282	WNT9A
cg17186168	chr2:11809607-11810433	NTSR2
cg17187287	chr4:115519423-115520932	UGT8
cg17199181	chr18:74799145-74800038	MBP
cg17202086	chr11:77122737-77123088	PAK1
cg17222164	chr17:43339125-43339832	SPATA32; MAP3K14-AS1
cg17222500	chr12:85306283-85306829	SLC6A15
cg17228900	chr6:391189-393790	IRF4
cg17236169	chr11:64480200-64481344	NRXN2
cg17241776	chr13:51417372-51418149	DLEU7; DLEU7-AS1
cg17246382	chr15:78556406-78557542	DNAJA4
cg17264670	chr6:153450993-153452792	RGS17
cg17266581	chr18:74799145-74800038	MBP
cg17267805	chr10:44879715-44882391	CXCL12
cg17269633	chr17:42733487-42734626	MEIOC
cg17275781	chr14:103739579-103740892	
cg17277199	chr2:24397646-24398194	FAM228A
cg17285225	chr3:8808962-8811280	OXTR
cg17292758	chr19:49636225-49636694	PPFIA3
cg17293936	chr3:185911345-185912228	DGKG
cg17295225	chr6:137814356-137815202	OLIG3
cg17299899	chr18:76737006-76741244	SALL3
cg17299935	chr17:74533282-74534566	PRCD; CYGB
cg17300544	chr17:75368689-75370506	SEPTIN9
cg17301902	chr9:80262447-80263701	GNA14

cg17302155	chr6:100060980-100062657	PRDM13
cg17306261	chr14:65016517-65016909	PPP1R36
cg17315500	chr12:103359249-103359629	
cg17321883	chr3:49755764-49757317	AMIGO3; GMPPB; RNF123
cg17333109	chr14:37049334-37051726	NKX2-8
cg17368760	chr20:23028404-23032218	THBD
cg17373442	chr3:142837886-142840838	CHST2
cg17380661	chr6:100912072-100913337	SIM1
cg17384889	chr6:28226980-28227483	NKAPL; ZKSCAN4
cg17386213	chr6:108485672-108490539	NR2E1
cg17403609	chr5:180075689-180076906	FLT4
cg17409893	chr19:42901017-42901375	LIPE-AS1
cg17419241	chr19:19738573-19739821	LAPAR2
cg17429382	chr19:3785512-3786653	MATK
cg17437939	chr7:97361133-97363018	TAC1
cg17438030	chr5:72526204-72526497	
cg17466857	chr7:27225051-27225629	HOXA11; HOXA11-AS
cg17477445	chr12:64061837-64062774	DPY19L2
cg17478228	chr19:11590296-11593643	ELAVL3
cg17478979	chr6:149771837-149772855	ZC3H12D
cg17480760	chr8:86350766-86351196	CA3
cg17493885	chr5:176558853-176561652	NSD1
cg17495087	chr8:96085270-96085747	NDUFAF6
cg17503456	chr3:138663719-138666346	FOXL2; FOXL2NB
cg17536595	chr6:43612715-43613366	RSPH9
cg17538572	chr10:94833273-94835256	CYP26A1
cg17542408	chr11:78673008-78673213	TENM4
cg17555373	chr13:53774265-53776345	
cg17555825	chr5:76923888-76924502	
cg17566118	chr10:8091375-8098329	GATA3; GATA3-AS1
cg17567560	chr10:105036629-105038084	INA
cg17577122	chr22:19510872-19512254	CLDN5
cg17594131	chr17:43044370-43046424	C1QL1
cg17603689	chr1:50882998-50883426	DMRTA2
cg17630392	chr5:32709911-32714450	NPR3
cg17666539	chr19:7926591-7928752	EVI5L
cg17689799	chr2:27529084-27531465	TRIM54; UCN
cg17694351	chr7:27252682-27253835	
cg17694795	chr8:67873389-67875600	TCF24
cg17697633	chr6:10419400-10420323	TFAP2A
cg17712694	chr5:16179065-16180420	MARCH11
cg17716617	chr1:33219428-33220028	KIAA1522
cg17737146	chr3:169529876-169530435	LRRC34
cg17737681	chr2:172951290-172952159	DLX1
cg17757602	chr5:42951077-42952410	
cg17760405	chr6:100914947-100915245	
cg17768491	chr19:41018526-41019486	SPTBN4
cg17780246	chr19:54481413-54481955	CACNG8
cg17780956	chr4:156297602-156298094	MAP9
cg17787134	chr17:47072821-47076042	IGF2BP1

cg17794299	chr20:623089-623398	
cg17796010	chr20:26188639-26190348	MIR663AHG; MIR6633
cg17800654	chr1:50884229-50891471	DMRTA2
cg17802942	chr3:138663719-138666346	FOXL2; FOXL2NB
cg17812788	chr17:40332598-40333471	KCNH4
cg17818432	chrX:134155997-134156602	RTLB8
cg17840719	chr5:178367621-178368725	ZNF454
cg17853216	chr10:81002110-81003687	ZMIZ1
cg17858328	chr1:92945908-92952609	GFI1
cg17882660	chr13:100637113-100637472	ZIC2
cg17885226	chr6:105388191-105389545	LIN28B; LIN28B-AS1
cg17890928	chr17:48545571-48546900	ACSF2; CHAD
cg17900356	chr3:53190510-53190764	
cg17904852	chr3:48698336-48701667	CELSR3
cg17912835	chr2:105478601-105479188	
cg17916490	chr2:11809607-11810433	NTSR2
cg17928883	chr5:2038528-2038949	
cg17928920	chr1:967967-970238	AGRN
cg17930034	chr14:65016517-65016909	PPP1R36
cg17935297	chr19:19651843-19652047	CILP2
cg17941572	chr1:63782395-63790471	FOXD3; FOXD3-AS1
cg17942573	chr5:16935556-16936408	MYO10
cg17960926	chr19:42901017-42901375	LIPE-AS1
cg17965019	chr6:27858167-27858564	H3C12
cg17968795	chr2:99552186-99553831	KIAA1211L
cg17969084	chr7:27190275-27191115	HOXA-AS3
cg17971015	chr19:12305553-12306304	
cg17974166	chr1:23279554-23280569	LACTBL1
cg17975443	chr17:59531724-59535254	TBX4
cg17982102	chr18:9707753-9709311	RAB31
cg17985646	chr7:35292986-35294674	TBX20
cg17988460	chr10:134597358-134602649	NKX6-2
cg17993027	chr8:82542799-82543475	IMPA1P1
cg17996619	chr10:134597358-134602649	NKX6-2
cg18004756	chr8:102504479-102504841	GRHL2
cg18008019	chr13:100641335-100642188	
cg18009321	chr20:25128765-25129610	
cg18016826	chr5:176558853-176561652	NSD1
cg18028999	chr15:81426500-81426702	CFAP161
cg18030386	chr19:4944133-4944498	UHRF1
cg18035229	chr8:70981874-70984888	PRDM14
cg18049676	chr5:177540208-177541234	N4BP3
cg18055610	chr6:30095174-30095610	
cg18081258	chr14:21492736-21494270	NDRG2
cg18096962	chr5:72526204-72526497	
cg18106312	chr14:51560117-51562487	TRIM9
cg18110168	chrX:50556225-50557179	SHROOM4
cg18116971	chr19:3285857-3286121	CELF5
cg18118033	chr1:26686517-26687281	CRYBG2
cg18129621	chr22:29875920-29877242	NEFH

cg18146843	chr1:53098630-53099303	SHISAL2A
cg18156204	chr17:71948479-71949255	
cg18177414	chr7:149389655-149389976	
cg18182111	chr5:37834672-37835128	GDNF; GDNF-AS1
cg18182148	chr1:92945908-92952609	GFI1
cg18202336	chr15:99190447-99194559	IGF1R; IRAIN
cg18209835	chr7:6543016-6543598	GRID2IP
cg18210732	chr11:69517841-69519929	FGF19
cg18233405	chr8:98289605-98290404	TSPYL5
cg18235050	chr12:126675562-126676195	
cg18245660	chr8:54791846-54795141	RGS20
cg18247055	chr10:22634001-22634862	SPAG6
cg18255595	chr13:20735044-20736157	GJA3
cg18260625	chr5:42951077-42952410	
cg18269826	chr15:101458287-101460223	LRRK1
cg18279094	chr1:63782395-63790471	FOXD3; FOXD3-AS1
cg18285309	chr1:40156931-40158514	HPCAL4
cg18309286	chr11:77122737-77123088	PAK1
cg18314424	chr4:184718264-184719754	STOX2
cg18326021	chr10:106399568-106402812	SORCS3
cg18331004	chr20:1783825-1784552	
cg18339788	chr19:41641308-41642082	
cg18342279	chr4:48492118-48493589	ZAR1
cg18342462	chr11:111385338-111385712	BTG4; c11orf88
cg18343437	chr8:142528186-142529029	
cg18349835	chr7:158936508-158938492	VIPR2
cg18350739	chr11:68622723-68623252	
cg18354742	chr19:38754847-38755699	SPINT2
cg18356785	chr11:47611139-47612438	C1QTNF4
cg18356974	chr7:128809090-128809393	TSPAN33
cg18358869	chr10:102807775-102808271	
cg18365406	chr19:11471906-11472299	PLPPR2
cg18366919	chr19:15344092-15344419	EPHX3
cg18368487	chr10:128993506-128995167	DOCK1
cg18406197	chr3:13590415-13591008	FBLN2
cg18409528	chr2:74740456-74743795	TLX2
cg18410627	chr9:129677707-129678009	RALGPS1
cg18418538	chr19:48996865-48997114	LMTK3
cg18423852	chr7:150652808-150653080	KCNH2
cg18424634	chr3:147126989-147128999	ZIC1
cg18429863	chr8:73448660-73449725	KCNB2
cg18438793	chr1:40149432-40150013	HPCAL4
cg18443359	chr5:134374386-134376751	c5orf66
cg18449964	chr18:72916108-72917233	ZADH2
cg18451114	chr1:38510103-38513642	POU3F1
cg18454916	chr8:74005022-74005856	SBSPON
cg18456523	chr5:54516268-54516919	MCIDAS
cg18467168	chr3:13114628-13115245	IQSEC1
cg18468354	chr14:36993489-36994488	
cg18470455	chrX:102000690-102001558	BHLHB9; ARMCX5-GPRASP2

cg18471993	chr6:100917206-100917523	
cg18477204	chr14:70038109-70040302	CCDC177
cg18485193	chr5:16935556-16936408	MYO10
cg18485844	chr11:2290105-2292932	ASCL2
cg18486102	chr12:50297581-50297988	FAIM2
cg18488157	chr6:29521111-29521833	OR2I1P
cg18489434	chr14:77227570-77228691	VASH1
cg18514820	chr10:17270431-17272617	VIM; VIM-AS1
cg18529845	chr2:31805294-31806403	SRD5A2
cg18536148	chr17:59531724-59535254	TBX4
cg18536496	chrX:101906002-101907017	ARMCX5-GPRASP2; GPRASP1
cg18557131	chr13:100620242-100624348	ZIC5
cg18558647	chr15:93198375-93199181	FAM174B
cg18559739	chr6:4775132-4777550	CDYL
cg18560204	chr15:83951981-83953930	BNC1
cg18564989	chr1:226411008-226411880	MIXL1
cg18567682	chr4:128544032-128544903	
cg18579862	chr19:58094740-58095764	ZIK1
cg18583378	chr7:150715309-150716404	ATG9B
cg18587476	chr2:24397646-24398194	FAM228A
cg18588589	chr6:29521111-29521833	OR2I1P
cg18591496	chr19:41595896-41596140	CYP2A13
cg18603228	chr3:13590415-13591008	FBLN2
cg18607529	chr7:50343758-50344519	IKZF1
cg18620571	chr14:103593235-103593923	TNFAIP2
cg18623980	chr2:45240373-45241579	
cg18630040	chr6:46702737-46703316	PLA2G7
cg18630667	chr19:37095681-37096589	ZNF529; ZNF529-AS1; ZNF382
cg18633684	chr12:104697349-104697984	TXNRD1; EID3
cg18638914	chr4:778662-780592	CPLX1
cg18647259	chr17:36609706-36610320	ARHGAP23
cg18671950	chr15:48936811-48938577	FBN1
cg18675043	chr10:105452339-105453230	SH3PXD2A
cg18675097	chr6:28226980-28227483	NKAPL; ZKSCAN4
cg18691800	chr6:17280740-17282334	RBM24
cg18694169	chr6:28226980-28227483	NKAPL; ZKSCAN4
cg18696576	chr6:34202568-34206193	HMGA1
cg18716164	chr19:30015782-30021367	VSTM2B
cg18723937	chr7:37955623-37956555	SFRP4; EPDR1
cg18724565	chr7:5632336-5634555	FSCN1
cg18725867	chr5:37834672-37835128	GDNF; GDNF-AS1
cg18726691	chr15:90039465-90039984	RHCG
cg18732064	chr14:23355739-23356402	REM2
cg18732172	chr11:504190-504659	RNH1
cg18740893	chr3:136537560-136539204	SLC35G2
cg18770350	chr1:236849473-236850323	ACTN2
cg18772588	chr20:61806255-61810867	MIR124-3
cg18786873	chr1:110610266-110613303	ALX3
cg18788664	chr1:221067448-221068185	
cg18790597	chr4:17782643-17783624	FAM184B

cg18794404	chr10:22540708-22542739	
cg18798922	chr2:171569878-171573904	SP5
cg18803104	chr6:28956107-28956578	HCG15
cg18810664	chr10:31073836-31074433	
cg18811550	chr7:27198183-27198514	
cg18815647	chr13:25743999-25746127	AMER2
cg18815943	chr1:47881897-47883065	FOXE3
cg18818834	chr6:52226754-52228006	PAQR8
cg18833573	chr8:144822012-144822805	FAM83H-AS1; IQANK1
cg18840956	chr5:95768875-95769080	PCSK1
cg18853935	chr7:25896714-25896925	
cg18855096	chr9:124061806-124062229	GSN
cg18859089	chr2:232276743-232277135	
cg18862481	chr3:129693128-129694841	TRH
cg18868483	chr6:71665361-71667132	B3GAT2
cg18892212	chr16:31498651-31500564	SLC5A2
cg18914211	chr6:29795554-29796594	HLA-G
cg18918321	chr8:41424342-41425300	
cg18932798	chr10:105036629-105038084	INA
cg18940113	chr2:161263872-161264460	RBMS1
cg18949300	chr6:167275721-167276626	RPS6KA2
cg18952647	chr15:83951981-83953930	BNC1
cg18952796	chr7:98245806-98247759	NPTX2
cg18953784	chr11:64739011-64739750	MAJIN
cg18956481	chr20:52789253-52790986	CYP24A1
cg18969232	chr2:45155196-45157049	
cg18988170	chr18:21718863-21719550	CABYR
cg18991611	chr8:49468684-49468959	
cg19003797	chr6:137818168-137819372	
cg19012731	chr7:86688833-86689238	KIAA1324L
cg19029181	chr3:147130343-147130577	ZIC1
cg19037922	chr4:108852496-108853387	CYP2U1
cg19043574	chr2:99438298-99439884	KIAA1211L
cg19045644	chr4:57975861-57976916	IGFBP7; IGFBP7-AS1
cg19046725	chr3:189838081-189838879	P3H2; P3H2-AS1
cg19047707	chr2:233350279-233352974	ECEL1
cg19059495	chr6:30095174-30095610	
cg19063061	chr19:49935752-49936275	SLC17A7
cg19078576	chr5:17217789-17219021	BASP1; BASP1-AS1
cg19127283	chr3:32858195-32860506	TRIM71
cg19129369	chr4:155664820-155665833	LRAT
cg19142026	chr7:27169573-27170638	HOXA3; HOXA4, HOXA4-AS2
cg19153828	chr2:127782614-127782829	
cg19155007	chr3:183542497-183543804	MAP6D1
cg19156875	chr17:43971411-43975040	MAPT; MAPT-AS1; MAPT-IT1
cg19157853	chr11:126286452-126286870	
cg19186145	chr2:45169506-45171884	SIX3
cg19198568	chr7:28995306-28998541	TRIL
cg19201719	chr9:214587-215431	DOCK8; DOCK8-AS1
cg19244428	chr6:3227026-3229688	TUBB2B

cg19250799	chr1:47909713-47911020	
cg19250989	chr1:147752860-147753165	NBPF8
cg19267596	chr19:15334123-15334822	
cg19267861	chr20:61806255-61810867	
cg19275261	chr18:5628325-5630785	EPB41L3
cg19281363	chr13:42030914-42032667	RGCC2
cg19283506	chr2:101033607-101034296	CHST10
cg19287823	chr6:155316300-155317197	TIAM2
cg19300568	chr4:2059929-2063181	NAT8L
cg19306047	chr19:19738573-19739821	LAPAR2
cg19320476	chr11:82443436-82444976	FAM181B
cg19337852	chr19:38754847-38755699	SPINT2
cg19355186	chr19:12305553-12306304	
cg19360104	chr1:154474108-154475699	TDRD10; SHE
cg19367047	chr19:5076032-5076905	KDM4B
cg19370054	chr2:98962874-98964187	CNGA3
cg19375537	chr1:46951169-46951792	
cg19380001	chr1:50884229-50891471	DMRTA2
cg19389884	chr11:1357627-1359081	
cg19396878	chr19:40971477-40973393	SPTBN4; BLVRB
cg19397703	chr19:43967248-43968625	LYPD3
cg19402991	chr2:164204741-164205039	
cg19403104	chr19:50030982-50031300	RCN3
cg19423622	chr7:158361910-158362970	PTPRN2
cg19424265	chr20:825268-826276	FAM110A
cg19429281	chr19:53496733-53497028	ZNF702P
cg19435033	chr17:19648140-19648492	ALDH3A1
cg19442495	chr3:142837886-142840838	CHST2
cg19451673	chr12:48397890-48398731	COL2A1
cg19456540	chr14:60975733-60978180	SIX6
cg19480724	chr7:27284640-27286237	EVX1
cg19485202	chr19:18899038-18902284	COMP
cg19499452	chr14:105830606-105830846	PACS2
cg19499748	chr7:28995306-28998541	TRIL
cg19504702	chr5:153862143-153862451	
cg19507206	chr10:103043991-103044480	
cg19513834	chr2:105468852-105473488	POU3F3; PANTR1
cg19517718	chr2:1746834-1748971	PXDN
cg19519964	chr2:223288923-223290013	SGPP2
cg19544662	chr2:88751336-88752865	FOXI3
cg19545623	chr4:40751843-40752493	NSUN7
cg19553869	chr11:66034752-66035054	KLC2; RAB1B
cg19554255	chr17:75368689-75370506	SEPTIN9
cg19585816	chr8:41753341-41755250	ANK1
cg19590532	chr1:25255528-25259005	RUNX3
cg19590598	chr2:127782614-127782829	
cg19592439	chr3:138663719-138666346	FOXL2; FOXL2NB
cg19594218	chr1:62660556-62660946	L1TD1
cg19595750	chr12:133065844-133066393	FBRSL1
cg19609502	chr22:40390485-40391490	FAM83F

cg19610869	chr5:157098264-157099041	SOX30
cg19619405	chr12:43944720-43946285	ADAMTS20
cg19628148	chr3:126113548-126113967	CFAP100; CCDC37-DT
cg19632532	chr6:3227026-3229688	TUBB2B
cg19636302	chr19:46032305-46032855	OPA3
cg19647755	chr8:49293202-49293547	
cg19651694	chr11:71954817-71955659	PHOX2A
cg19656282	chr2:74740456-74743795	TLX2
cg19657174	chr22:19746156-19746369	TBX1
cg19665644	chr11:13030331-13032584	RASSF10; RASSF10-DT
cg19685491	chr12:2800140-2801062	CACNA1C; CACNA1C-AS1
cg19690984	chr6:29521111-29521833	OR2I1P
cg19701540	chr10:134597358-134602649	NKX6-2
cg19706516	chr1:151103686-151106100	SEMA6C
cg19712603	chr13:53419898-53422872	PCDH8
cg19714957	chr19:19843483-19843943	ZNF14
cg19715410	chr1:29138502-29139348	OPRD1
cg19717586	chr11:131780329-131781532	NTM
cg19722720	chr1:113050814-113052301	WNT2B
cg19729116	chr6:26501726-26502107	BTN1A1
cg19734163	chr7:138720045-138721019	ZC3HAV1L
cg19751300	chr18:44336184-44337110	ST8SIA5
cg19752891	chr19:4304543-4305122	FSD1
cg19756821	chr1:54940347-54940753	
cg19760241	chr17:35291900-35300875	LHX1; LHX1-DT
cg19761115	chr5:178487147-178487921	ZNF354C
cg19761848	chr2:237071795-237078762	GBX2
cg19763319	chr19:50316212-50316469	FUZ
cg19763809	chr2:98703355-98703889	VWA3B
cg19767215	chr19:38754847-38755699	SPINT2
cg19779211	chr11:2465172-2465648	KCNQ1
cg19788741	chr5:101632050-101632373	SLCO4C1
cg19796981	chr22:50438258-50438776	IL17REL
cg19806642	chr5:178421226-178422337	GRM6
cg19807257	chr1:46951169-46951792	
cg19809453	chr1:6507208-6509186	ESPN
cg19820372	chr11:111410933-111412199	LAYN
cg19837790	chr10:97802872-97804262	CCNJ; ENTPD1-AS1
cg19841005	chr19:3687787-3688262	PIP5K1C
cg19850348	chr15:55879793-55881228	PYGO1
cg19851909	chr11:94134227-94134848	GPR83
cg19852958	chr4:13543563-13546494	NKX3-2
cg19859290	chr11:20618198-20619920	SLC6A5
cg19861117	chr19:46456210-46456503	NOVA2
cg19863210	chr8:142138209-142139342	DENND
cg19866866	chr1:182025605-182026511	ZNF648
cg19867649	chr11:12029738-12030841	DKK3
cg19870512	chr12:4918274-4919657	KCNA6
cg19875368	chr6:91320891-91321219	
cg19879075	chr10:73846807-73848233	SPOCK2

cg19883813	chr10:8091375-8098329	GATA3; GATA3-AS1
cg19885761	chr5:175223610-175224679	CPLX2
cg19900615	chr1:41267920-41268398	KCNQ4
cg19901801	chr1:114696887-114697185	SYT6
cg19904122	chr19:3285857-3286121	CELF5
cg19908577	chr4:115519423-115520932	UGT8
cg19908812	chr4:164252955-164253471	NPY1R
cg19909787	chr10:124907284-124911035	HMX2
cg19925849	chr19:54445232-54445745	CACNG7
cg19929126	chr7:28995306-28998541	TRIL
cg19947104	chr11:17756057-17758286	KCNC1
cg19954017	chr17:80332183-80333671	UTS2R
cg19955500	chr5:32709911-32714450	NPR3
cg19961043	chr17:48545571-48546900	ACSF2; CHAD
cg19968840	chr15:45408574-45409528	DUOXA2
cg19974428	chr7:98467024-98468058	TMEM130
cg19975933	chr11:74022429-74022703	P4HA3
cg19988449	chr15:83951981-83953930	BNC1
cg19996355	chr19:19729128-19729814	PBX4
cg19998368	chr12:64784011-64784664	C12orf56
cg20000718	chr4:41869175-41869459	
cg20002504	chr2:241496577-241497600	ANKMY1
cg20011402	chr10:17495408-17496721	ST8SIA6
cg20014398	chr11:31825744-31826967	PAX6
cg20018469	chr10:17270431-17272617	VIM; VIM-AS1
cg20019985	chr11:32008803-32009044	RCN1
cg20022589	chr18:21718863-21719550	CABYR
cg20049415	chr20:21376359-21378245	NKX2-4
cg20056542	chr15:53080459-53083699	ONECUT1
cg20070026	chr14:102247661-102248279	PPP2R5C
cg20075156	chr4:11430236-11431256	HS3ST1
cg20078466	chr7:50343758-50344519	IKZF1
cg20080624	chr7:96650222-96651551	DLX5
cg20085077	chrX:100740222-100740601	ARMCX4
cg20092531	chr18:54788960-54789194	
cg20092892	chr12:52257745-52258128	
cg20102877	chr2:27665252-27665670	KRTCAP3
cg20106459	chr19:55865825-55866324	COX6B2
cg20113824	chr8:70946891-70947299	
cg20122943	chr19:54483022-54483572	CACNG8
cg20129213	chr8:104510871-104513913	RIMS2
cg20146541	chr1:248020331-248021252	TRIM58
cg20150591	chr7:3341451-3341733	SDK1
cg20166714	chr12:113592204-113592620	CFAP73
cg20182358	chr3:158288801-158289271	MLF1
cg20185461	chr19:18899038-18902284	COMP
cg20192747	chr18:44772993-44775577	SKOR2
cg20209308	chr22:19136294-19138512	GSC2
cg20219381	chr8:101117923-101118693	RGS22
cg20222926	chr7:121945346-121946235	FEZF1; FEZF1-AS1

cg20227255	chr6:43142014-43142217	SRF
cg20232102	chr19:38746639-38747379	PPP1R14A
cg20232986	chr6:100915603-100915883	
cg20247486	chr1:119543822-119544339	
cg20255775	chr12:48397890-48398731	COL2A1
cg20270188	chr1:156611640-156612447	BCAN
cg20275528	chr17:75368689-75370506	SEPTIN9
cg20284629	chr1:47909713-47911020	
cg20289688	chr11:17740790-17743779	MYOD1
cg20291049	chr2:105468852-105473488	POU3F3; PANTR1
cg20297199	chr14:54420185-54422958	BMP4
cg20300175	chr17:27918162-27918398	ANKRD13B-AS1
cg20305024	chr15:40573629-40576118	ANKRD63
cg20318845	chr2:45235512-45237792	SIX2
cg20340508	chr21:34442207-34444543	OLIG1
cg20358834	chr11:66623621-66626614	LRNF4
cg20365074	chr2:189156417-189157719	GULP1
cg20370678	chr17:58216297-58216505	
cg20377305	chr7:96650222-96651551	DLX5
cg20381020	chr11:66623621-66626614	LRNF4
cg20383948	chr21:46897842-46898158	COL18A1
cg20384898	chr6:1624186-1625468	GMDS
cg20392240	chr8:104383410-104384109	CTHRC1
cg20395967	chr20:26188639-26190348	MIR663AHG; MIR6633
cg20399509	chr21:47717236-47718059	YBEY
cg20399616	chr12:25055600-25056246	BCAT1
cg20401551	chr22:20790639-20792665	SCARF2
cg20419623	chr20:48598960-48599657	SNAI1
cg20442599	chr6:108478524-108479558	OSTM1-AS1
cg20443254	chr12:108168987-108169570	ASCL4
cg20447655	chr8:104383410-104384109	CTHRC1
cg20449685	chr19:58545116-58545897	ZSCAN1
cg20449692	chr3:170136243-170137886	CLDN11
cg20482698	chr1:236849473-236850323	ACTN2
cg20486569	chr22:19510872-19512254	CLDN5
cg20491838	chr6:1604607-1615866	FOXC1
cg20495645	chr22:45403037-45406372	PHF21B
cg20527270	chr8:49468684-49468959	
cg20530204	chr6:33244678-33245554	B3GALT4
cg20532999	chr15:69323960-69324232	NOX5
cg20536041	chr2:45028956-45029318	
cg20555674	chr17:37321483-37322099	ARL5C
cg20555682	chr11:8289533-8290322	LMO1
cg20557801	chr15:68115486-68122575	SKOR1
cg20569128	chrX:136655910-136657085	ZIC3
cg20569287	chr8:144510851-144513957	MAFA
cg20574490	chr8:102504479-102504841	GRHL2
cg20577019	chr1:150121696-150123078	PLEKHO1
cg20585530	chr14:60975733-60978180	SIX6
cg20585869	chr8:24770909-24772547	NEFM

cg20597013	chr1:244893442-244894366	
cg20611911	chr1:3566446-3569636	TP73; WRAP73
cg20616414	chr9:95946148-95947835	WNK2
cg20636352	chr2:200468552-200468958	
cg20648847	chr11:66326640-66326840	ACTN3
cg20673829	chr7:158936508-158938492	VIPR2
cg20684180	chr3:38080629-38081187	DLEC1
cg20699586	chr1:227748424-227748860	
cg20701182	chr2:24300061-24300294	FAM228B; SF3B6
cg20707679	chr19:3434930-3435417	NFIC
cg20744625	chr10:64574661-64578778	EGR2
cg20748955	chr11:118016238-118016984	SCN4B
cg20749741	chr2:263401-265238	SH3YL1; ACP1
cg20750832	chr5:170741604-170742751	
cg20761860	chr5:131991555-131992304	IL13; TH2LCRR
cg20769177	chr17:44928288-44929690	WNT9B
cg20771178	chr11:8615398-8615889	STK33
cg20776829	chr4:90758009-90758870	SNCA
cg20785796	chr2:200320503-200329681	SATB2
cg20787173	chr6:133562087-133563586	EYA4
cg20797142	chr1:6051596-6052955	NPHP4; KCNAB2
cg20800509	chr8:104510871-104513913	
cg20809087	chr15:72611947-72612802	CELF6
cg20810478	chr1:248020331-248021252	TRIM58
cg20817902	chr4:81123509-81124318	PRDM8
cg20844851	chr6:117591534-117592279	VGLL2
cg20859731	chr20:36226618-36226841	
cg20885782	chr4:41875446-41875794	
cg20902783	chr14:21492736-21494270	NDRG2
cg20912770	chr11:2290105-2292932	ASCL2
cg20923245	chr12:104697349-104697984	TXNRD1; EID3
cg20927661	chr2:5836069-5837643	SOX11
cg20935165	chr2:172972805-172973242	DLX2-DT
cg20944305	chr3:152552603-152553712	P2RY1
cg20945085	chr2:74875129-74875562	M1AP
cg20950465	chr1:53067881-53068608	GPX7
cg20953187	chr3:45837481-45838256	SLC6A20
cg20961591	chr19:38754847-38755699	SPINT2
cg20971220	chr19:50554013-50554492	
cg20977794	chr14:51560117-51562487	TRIM9
cg20978694	chr4:2059929-2063181	NAT8L
cg20984053	chr7:156802171-156804414	MNX1; MNX1-AS1
cg20985450	chr13:100620242-100624348	ZIC5
cg20986370	chr4:57975861-57976916	IGFBP7; IGFBP7-AS1
cg21000072	chr1:3566446-3569636	TP73; WRAP73
cg21002957	chr19:15568028-15569227	RASAL3
cg21006031	chr19:43967248-43968625	LYPD3
cg21007414	chr11:76381450-76382295	LRRC32
cg21011139	chr11:7694712-7695685	CYB5R2
cg21033440	chr11:65409637-65410127	SIPA1

cg21038156	chr7:158936508-158938492	VIPR2
cg21039708	chr14:57278710-57279116	OTX2-AS1
cg21046967	chr17:37761692-37763551	NEUROD2
cg21051519	chr5:137224987-137225477	PKD2L2
cg21052682	chr11:69517841-69519929	FGF19
cg21073930	chr19:12984133-12985466	MAST1
cg21074827	chr3:158288801-158289271	MLF1
cg21079345	chr7:134143116-134144063	AKR1B1
cg21101720	chr17:27939299-27940770	ANKRD13B
cg21104276	chr7:90893568-90896683	FZD1
cg21115608	chr13:20735044-20736157	GJA3
cg21144922	chr1:109203594-109204378	HENMET1
cg21158087	chr17:58216297-58216505	
cg21158411	chr9:130689379-130690215	PIP5KL1
cg21172458	chr8:86350766-86351196	CA3
cg21195185	chr4:76555367-76556079	CDKL2
cg21200353	chr3:183145428-183146790	MCF2L2
cg21200408	chr20:21376359-21378245	NKX2-4
cg21200656	chr20:21376359-21378245	NKX2-4
cg21207028	chr20:327377-328977	NRSN2
cg21237687	chr17:6898821-6900427	ALOX12
cg21248554	chr2:27664940-27665151	KRTCAP3
cg21249376	chr9:27528358-27528725	MOB3B
cg21265540	chr12:125534061-125534527	
cg21270860	chr1:38510103-38513642	POU3F1
cg21277995	chr6:391189-393790	IRF4
cg21282549	chr19:51925000-51925413	
cg21290042	chr1:224803638-224805314	CNIH3
cg21306240	chr1:38229839-38230888	EPHA10
cg21319323	chr2:177001222-177001783	HOXD-AS2
cg21325154	chr4:141489963-141490378	UCP1
cg21334513	chr6:30095174-30095610	
cg21346043	chr8:86350766-86351196	CA3
cg21347053	chr6:1624186-1625468	GMDS
cg21355828	chr7:149389655-149389976	
cg21361094	chr12:58021295-58022037	B4GALNT1
cg21383487	chr10:22623351-22625875	
cg21383810	chr12:65514879-65515863	WIF1
cg21384402	chr10:105036629-105038084	INA
cg21392341	chr1:119529820-119530712	TBX15
cg21404045	chr7:27231806-27233097	
cg21415530	chr8:140714586-140718259	KCNK9
cg21424782	chr20:48598960-48599657	SNAI1
cg21425842	chr6:26240698-26240951	HIST1H4F
cg21433558	chr17:40837023-40837525	CNTNAP1
cg21447550	chr1:223536843-223538628	SUSD4
cg21453443	chr1:228645197-228646434	HIST3H2BB; HIST3H2A
cg21463763	chr20:3713160-3713515	HSPA12B
cg21472506	chr2:63283937-63284147	OTX1
cg21475402	chr1:156611640-156612447	BCAN

cg21491107	chr16:85644948-85649027	GSE1
cg21497607	chr2:74425445-74426423	MTHFD2
cg21497990	chr7:155255099-155255311	EN2
cg21534423	chr19:24154458-24154759	
cg21535253	chr19:12758274-12758754	MAN2B1
cg21538208	chr4:4864457-4864834	MSX1
cg21540810	chr19:53141176-53141813	ZNF83
cg21554552	chr3:50377804-50378540	RASSF1
cg21570988	chr6:32116591-32117229	PRRT1
cg21572621	chr7:28995306-28998541	TRIL
cg21590264	chr5:37834672-37835128	GDNF
cg21609640	chr1:166889777-166890649	ILDR2
cg21610164	chr6:26273291-26273557	HIST1H2BI; H2BC10
cg21643403	chr8:104383410-104384109	CTHRC1
cg21647227	chr1:119526783-119527192	TBX15
cg21654383	chr2:85640970-85641259	CAPG
cg21658235	chr8:22456092-22456508	c8orf58
cg21684012	chr6:100912072-100913337	SIM1
cg21692846	chr11:134145560-134147180	GLB1L3
cg21706229	chr17:70112825-70114271	SOX9
cg21725888	chr6:43252735-43253476	TTBK1
cg21743907	chr7:129422998-129423355	
cg21762523	chr6:108497596-108497996	NR2E1
cg21773872	chr7:30721373-30722445	CRHR2
cg21773962	chrX:139585868-139588578	SOX3
cg21790626	chr19:58220190-58220517	ZNF154
cg21799346	chr11:2905024-2907024	CDKN1C
cg21800232	chr5:79864843-79866447	ANKRD34B
cg21801378	chr15:72611947-72612802	CELF6
cg21816330	chr17:27044169-27045049	RAB34
cg21820656	chr15:59157046-59157594	
cg21836358	chr1:63782395-63790471	FOXD3; FOXD3-AS1
cg21848143	chr1:39269730-39270251	
cg21856256	chr2:121200504-121200788	
cg21858380	chr2:119914127-119916663	C1QL2
cg21868134	chr1:45792420-45793301	HPDL
cg21872037	chr17:6616423-6617471	SLC13A5
cg21872764	chr22:19510872-19512254	CLDN5
cg21874829	chr22:38808785-38809017	
cg21875532	chr6:42109905-42110361	c6orf132
cg21883598	chr15:45403249-45404218	DUOX2
cg21884231	chr3:170303533-170303768	SLC7A14
cg21884421	chr15:65647918-65648491	IGDCC3
cg21889472	chr5:42992034-42992955	FLJ32255
cg21901946	chr7:127744195-127744554	
cg21908235	chr1:146551477-146551764	
cg21908638	chr3:101497831-101498648	NXPE3
cg21912308	chrX:48929637-48930361	PRAF2
cg21921384	chr4:57975861-57976916	IGFBP7; IGFBP7-AS1
cg21928701	chr19:821346-822669	PLPPR3

cg21940568	chr19:52207133-52207731	SPACA6
cg21945930	chr7:96631384-96631800	DLX6-AS1
cg21951457	chr10:123922851-123923542	TACC2
cg21962901	chr19:18543829-18549161	ISYNA1
cg22001496	chr8:69242921-69243803	c8orf34; c8orf34-as1
cg22010052	chr5:115298351-115299137	LVRN
cg22029275	chr13:25743999-25746127	AMER2
cg22053904	chr6:30227321-30228255	HLA-L; HCG17
cg22054918	chr4:81123509-81124318	PRDM8
cg22059073	chr22:17600564-17602611	
cg22066230	chr11:66034752-66035054	RAB1B
cg22070406	chr18:55019708-55021605	ST8SIA3
cg22076972	chr19:6740670-6741203	TRIP10
cg22078805	chr17:42431093-42432555	FAM171A2
cg22079161	chr4:7940564-7941853	AFAP1
cg22121557	chr6:28583935-28584289	ZBED9
cg22131234	chr7:54609735-54610140	VSTM2A
cg22144875	chr19:46032305-46032855	OPA3
cg22149516	chr5:101632050-101632373	SLCO4C1
cg22152688	chr7:97839863-97840222	BHLHA15
cg22153181	chr4:172733735-172735118	GALNTL6
cg22158769	chr2:39186778-39187968	ARHGEF33
cg22160000	chr1:45251963-45252292	BEST4
cg22167515	chr1:91190490-91192804	
cg22168369	chr1:85462586-85463435	MCOLN2
cg22178238	chr7:158361910-158362970	PTPRN2
cg22183049	chr5:139143966-139144177	
cg22186831	chr5:176543901-176544228	
cg22191696	chr17:71948479-71949255	
cg22195627	chr19:12305553-12306304	
cg22201588	chr19:11533199-11533619	CCDC151
cg22210157	chr3:46853728-46854138	PRSS50
cg22212691	chr2:220416864-220417869	OBSL1
cg22232327	chr18:53447075-53447842	LINC01415
cg22241820	chr15:68115486-68122575	SKOR1
cg22274539	chr12:103696091-103696418	c12orf42
cg22275864	chr8:37822487-37824008	ADRB3
cg22277636	chr2:218621280-218621498	DIRC3
cg22282405	chr6:50810643-50810994	TFAP2B
cg22282410	chr7:158379329-158381221	PTPRN2
cg22305167	chr6:29521111-29521833	OR2I1P
cg22311200	chr17:46695326-46695553	
cg22319311	chr8:98289605-98290404	TSPYL5
cg22322863	chr17:30813178-30815239	CDK5R1
cg22345692	chr5:32709911-32714450	NPR3
cg22346124	chr11:2290105-2292932	ASCL2
cg22348673	chr7:28995306-28998541	TRIL
cg22352818	chr2:97192978-97193383	
cg22367989	chr2:206546604-206547566	NRP2
cg22375192	chr15:99190447-99194559	IGF1R; IRAIN

cg22376688	chr12:49371691-49375550	WNT1
cg22399133	chr2:208988835-208989269	CRYGD
cg22402852	chr4:108852496-108853387	CYP2U1
cg22409276	chr15:40763163-40764196	CHST14
cg22410478	chr6:110678855-110679108	METLL24
cg22418737	chr7:139930066-139930356	
cg22433276	chr1:1072370-1072847	LINC01342
cg22443762	chr1:197890479-197890824	LHX9
cg22447539	chr7:5632336-5634555	FSCN1
cg22455914	chr18:7116853-7118241	LAMA1
cg22465516	chr17:54910497-54912470	DGKE; c17orf67
cg22468075	chr20:22548968-22549720	LINC00261
cg22471255	chr22:42315503-42316409	
cg22488797	chr5:134363093-134365146	PITX1
cg22490134	chr10:22623351-22625875	
cg22510337	chr19:58399946-58400276	ZNF814
cg22524657	chr1:47998900-47999517	
cg22531183	chr19:50554013-50554492	ZNF473
cg22535307	chr15:48936811-48938577	FBN1
cg22536150	chr3:142837886-142840838	CHST2
cg22539420	chr2:200320503-200329681	SATB2
cg22546696	chr15:41952536-41953222	MGA
cg22557662	chr19:38746639-38747379	PPP1R14A
cg22560214	chr4:13530848-13531244	LINC01097
cg22569587	chrX:9733155-9734064	GPR143
cg22571038	chr17:48585386-48586167	MYCBPAP
cg22571664	chr5:32709911-32714450	NPR3
cg22579265	chr10:94180316-94180754	MARK2P9
cg22592142	chr15:45408574-45409528	DUOXA2
cg22598028	chr3:44626326-44626794	ZNF660; ZNF660-197
cg22604123	chr1:32930501-32931061	ZBTB8B
cg22605919	chr17:42733487-42734626	MEIOC
cg22606658	chr4:150999251-151000706	DCLK2
cg22617773	chr6:29521111-29521833	OR2I1P
cg22645427	chr20:25128765-25129610	
cg22659676	chr5:139525538-139526227	
cg22660578	chr17:35291900-35300875	LHX1; LHX1-DT
cg22674699	chr2:176986425-176988291	HOXD9
cg22682811	chr20:21372042-21372463	
cg22690188	chrX:64626744-64628045	
cg22694470	chr1:92495368-92495964	EPHX4
cg22720790	chr19:58715221-58716033	ZNF274
cg22721334	chr19:58609339-58609988	ZSCAN18
cg22730047	chr1:161410143-161442503	
cg22730464	chr5:94955631-94957244	GPR150
cg22740547	chr11:13030331-13032584	RASSF10; RASSF10-DT
cg22758454	chr17:16569939-16570517	
cg22762844	chr2:95690858-95692431	MAL
cg22789900	chr1:226411008-226411880	MIXL1
cg22791936	chr6:137242316-137245442	SLC35D3

cg22792646	chr11:15136059-15136545	INSC
cg22802813	chr12:95941907-95942979	USP44
cg22807877	chr8:141107838-141110984	PEG13
cg22815953	chr20:48598960-48599657	SNAI1
cg22827250	chr5:134363093-134365146	PITX1
cg22836229	chr8:49647703-49647988	EFCAB1
cg22848598	chr8:38965014-38965423	ADAM32
cg22855900	chr5:72529100-72529976	
cg22856324	chr2:72371122-72375004	CYP26B1
cg22865058	chr11:2905024-2907024	CDKN1C
cg22871653	chr1:119535667-119535987	
cg22871668	chr6:133562087-133563586	EYA4
cg22876812	chr2:71115928-71116412	LINC01143
cg22878622	chr8:67873389-67875600	TCF24
cg22880677	chr8:1771362-1772760	ARHGEF10
cg22881914	chr14:52534582-52536722	NID2
cg22882665	chr2:45240373-45241579	
cg22884130	chr22:32026298-32026874	PISD
cg22892110	chr8:144798488-144799038	MAPK15
cg22900415	chr13:20735044-20736157	GJA3
cg22902177	chr3:129323916-129325332	PLXND1
cg22905274	chr4:154170369-154170600	TRIM2
cg22953960	chr10:22764709-22767050	
cg22958090	chr12:52626794-52627577	KRT7
cg22963915	chr19:3785512-3786653	MATK
cg22977876	chr2:172949243-172950126	DLX1
cg22979783	chr2:72371122-72375004	CYP26B1
cg22983083	chr19:8656789-8657917	ADAMTS10
cg22984571	chr3:186490603-186490813	
cg23003534	chr10:105036629-105038084	INA
cg23016129	chr10:22634001-22634862	SPAG6
cg23026864	chr5:140305713-140307193	HA1,2,3,4,5,6,7,8,9,10,11,12
cg23027521	chr1:247170869-247171434	ZNF625; ZNF625-ZNF20
cg23029526	chr1:21616381-21617101	ECE1
cg23030278	chr15:55879793-55881228	PYGO1
cg23039195	chr10:22623351-22625875	
cg23043245	chr6:34433412-34434371	PACSIN1
cg23049458	chr1:62660556-62660946	L1TD1
cg23052826	chr2:232478360-232479925	
cg23054189	chr1:248020331-248021252	TRIM58
cg23058177	chr2:10219803-10221016	CYS1
cg23064609	chr2:175208416-175209076	
cg23068913	chr10:23462225-23463889	
cg23089825	chr1:170630457-170630851	
cg23091758	chr11:9025096-9026315	NRIP3
cg23092040	chr14:65068369-65068668	
cg23095743	chr10:104000255-104001741	PITX3
cg23097402	chr1:50882998-50883426	DMRTA2
cg23118086	chr15:65360025-65360542	RASL12
cg23119604	chr6:30227321-30228255	HCG17; HLA-L

cg23125200	chr13:20806017-20806867	GJB6
cg23132624	chr13:33589929-33591428	KL
cg23136139	chr10:43697778-43698177	RASGEF1A
cg23148701	chr12:64061837-64062774	DPY19L2
cg23152743	chr7:139167481-139168712	KLRG2
cg23154526	chr15:60296136-60298520	FOXB1
cg23187653	chr5:95768875-95769080	PCSK1
cg23194354	chr7:3340355-3341216	SDK1
cg23200020	chr19:35068407-35068897	SCGB1B2P
cg23208152	chr3:50382890-50383310	ZMYND10
cg23208513	chr8:101117923-101118693	RGS22
cg23217622	chr1:156405161-156406350	
cg23229261	chr2:63283937-63284147	OTX1
cg23231910	chr2:202897387-202901046	FZD7
cg23237183	chr2:73429284-73430647	NOTO
cg23244289	chr5:79330929-79331488	THBS4
cg23244790	chr5:140810495-140812617	4,5,6,7,8,9,10,11,12; PCDHG
cg23246885	chr2:202897387-202901046	FZD7
cg23248887	chr14:38678246-38680937	SSTR1
cg23255835	chr2:29337984-29338909	CLIP4
cg23258881	chr1:150121696-150123078	PLEKHO1
cg23265500	chr14:65016517-65016909	PPP1R36
cg23266594	chr5:149546028-149546988	CDX1
cg23267759	chr18:19744937-19752363	GATA6; GATA6-AS1
cg23290344	chr8:24770909-24772547	NEFM
cg23297477	chr16:66638255-66639561	CMTM3
cg23302649	chr14:29243404-29243763	LINC01551
cg23305408	chr1:32169538-32169869	COL16A1
cg23305567	chr19:54369388-54369809	MYADM
cg23310835	chr19:24154458-24154759	
cg23316253	chr10:8076003-8077261	
cg23318063	chr14:58331677-58333121	SLC35F4
cg23319982	chr19:41641308-41642082	
cg23322933	chr14:61108955-61109786	SALRNA1
cg23325963	chr2:45159894-45160112	
cg23337116	chr4:53728038-53729000	RASL11B
cg23353952	chr22:29875920-29877242	NEFH
cg23356769	chrX:129243675-129245575	ELF4
cg23376821	chr6:45389421-45391646	RUNX2
cg23402821	chr17:35291900-35300875	LHX1; LHX1-DT
cg23413104	chr2:118981770-118982466	
cg23426238	chr7:103086345-103086840	SLC26A5
cg23428985	chr2:29337984-29338909	CLIP4
cg23445461	chr5:140864528-140864748	,8,9,10,11,12; PCDHGB1,2,3
cg23461615	chr7:103085711-103086132	SLC26A5
cg23462514	chr4:1107126-1107728	RNF212
cg23463269	chr11:15136059-15136545	INSC
cg23479922	chr5:16179065-16180420	MARCH11
cg23495748	chr5:175223610-175224679	CPLX2
cg23497383	chr2:239140097-239140659	

cg23501406	chr11:636907-640628	DRD4
cg23505145	chr19:12996087-12997039	KLF1
cg23511432	chr5:137610106-137610311	GFRA3
cg23511613	chr2:88751336-88752865	FOXI3
cg23516463	chr10:88391747-88392242	
cg23524735	chr2:209271338-209271987	PTH2R
cg23528400	chr3:32858195-32860506	TRIM71
cg23543123	chr4:48485363-48486473	SLC10A4
cg23543615	chr19:19280999-19281560	MEF2B
cg23563234	chr5:140797163-140797701	[illegible]2,3,4,5,6,7,8,9,10; PCDHGB1,
cg23565942	chr10:21462129-21463808	NEBL; NEBL-AS1
cg23572908	chr7:158936508-158938492	VIPR2
cg23588217	chr1:119529820-119530712	TBX15
cg23597629	chr1:229567207-229570684	ACTA1
cg23610453	chr10:79470963-79471425	
cg23619365	chr13:112709885-112712665	SOX1-OT
cg23620340	chr1:63782395-63790471	FOXD3; FOXD3-AS1
cg23624808	chr18:32847285-32848130	ZSCAN30
cg23625660	chr5:135265849-135266517	FBXL21
cg23634087	chr20:50721311-50721912	ZFP64
cg23652859	chr19:59092692-59093273	CENPBDEP1
cg23663760	chr1:228246632-228247197	WNT3A
cg23666378	chr1:3662964-3664085	TP73-AS1
cg23681664	chr3:9745500-9746782	CPNE9
cg23684074	chr7:128470444-128471161	FLNC
cg23695133	chr20:21086140-21087175	
cg23697855	chr7:89950259-89950778	FAM237B
cg23719069	chr15:83875649-83877079	HDGFL3
cg23728669	chr11:63258379-63258804	HRASLS5
cg23735712	chr4:40858885-40859162	APBB2
cg23746497	chr6:105388191-105389545	LIN28B
cg23754431	chr11:69258150-69258544	
cg23763647	chr10:4868126-4868949	AKR1E2
cg23778596	chr7:149410859-149412351	KRBA1
cg23782001	chr7:138720045-138721019	ZC3HAV1L
cg23808946	chr2:45169506-45171884	SIX3
cg23816347	chr3:126113548-126113967	CCDC37-DT; CFAP100
cg23817096	chr6:1619094-1621094	
cg23837799	chr15:31683985-31685824	
cg23842255	chr22:29875920-29877242	NEFH
cg23844174	chr1:2064629-2064855	PRKCZ
cg23847109	chr1:40782221-40783145	COL9A2
cg23847712	chr4:9783036-9784960	DRD5
cg23858040	chr10:11059443-11060524	CELF2
cg23874437	chr19:38886110-38886615	SPRED3
cg23874561	chr4:81124469-81124845	PRDM8
cg23881278	chr11:113345069-113346328	DRD2
cg23882862	chr11:111169305-111170428	
cg23900203	chr12:127765277-127765515	
cg23901852	chr4:126235896-126238930	FAT4

cg23906738	chr14:36986363-36990576	NKX2-1; NKX2-1-AS1
cg23907926	chr12:131303094-131303836	STX2
cg23912429	chr10:17270431-17272617	VIM; VIM-AS1
cg23920953	chr14:70038109-70040302	CCDC177
cg23923856	chr1:113050814-113052301	WNT2B
cg23944005	chr6:26271346-26271588	HIST1H3G
cg23944251	chr10:25463757-25465639	GPR158; GPR158-AS1
cg23951961	chr4:42152767-42154984	BEND4
cg23964682	chr2:241496577-241497600	ANKMY1
cg23965061	chr8:22960385-22960927	TNFRSF10C
cg23971915	chr3:46742765-46743086	TMIE
cg23973429	chr8:102504479-102504841	GRHL2
cg23989963	chr15:83951981-83953930	BNC1
cg23994043	chr2:200320503-200329681	SATB2
cg23999695	chr15:89942593-89943853	
cg23999932	chr4:54975388-54976202	
cg24002887	chr7:64407590-64408299	
cg24013620	chr8:144798488-144799038	MAPK15
cg24015249	chr9:124498514-124498962	DAB2IP
cg24026757	chr18:12407716-12408242	SLMO1
cg24031355	chr10:22634001-22634862	SPAG6
cg24034005	chr14:97058944-97059522	
cg24035245	chr1:119535667-119535987	
cg24037897	chr10:16561605-16563822	C1QL3
cg24039697	chr10:8091375-8098329	GATA3; GATA3-AS1
cg24051554	chr1:151810461-151811684	C2CD4D
cg24073122	chr1:3566446-3569636	TP73; WARP73
cg24078363	chr14:77227570-77228691	VASH1
cg24084504	chr4:42399153-42400802	SHISA3
cg24087403	chr10:71077947-71079377	HK1
cg24098326	chr10:123922851-123923542	TACC2
cg24102266	chr1:33219428-33220028	KIAA1522
cg24104938	chr6:108485672-108490539	NR2E1
cg24106636	chr1:116380360-116382364	NHLH2
cg24125828	chr6:32116591-32117229	PRRT1
cg24127874	chr2:239147593-239149900	HES6
cg24144440	chr1:119526783-119527192	TBX15
cg24154839	chr4:46995129-46995872	GABRA4
cg24157892	chr17:59473061-59483266	TBX2
cg24159214	chr3:50402104-50402942	CACNA2D2
cg24161925	chr6:139116947-139117469	ECT2L
cg24166864	chr12:65514879-65515863	WIF1
cg24190603	chr6:84417598-84419378	SNAP91
cg24198558	chr3:132756986-132757895	TEMEM108
cg24199834	chr4:147559206-147561901	POU4F2
cg24213719	chr18:60263381-60264248	
cg24217704	chr2:166649910-166650966	GALNT3
cg24217844	chr6:137242316-137245442	SLC35D3
cg24225517	chr6:108485672-108490539	NR2E1
cg24239148	chr10:63422539-63423105	cabcoco1

cg24255928	chr10:97802872-97804262	CCNJ
cg24274579	chr17:59531724-59535254	TBX4
cg24276681	chr18:2905951-2907348	EMILIN2
cg24292235	chr12:8171337-8171790	
cg24300924	chr18:12407716-12408242	SLMO1
cg24306982	chr1:119549145-119551320	
cg24318763	chr7:139208443-139209069	CLEC2L
cg24319718	chr2:206546604-206547566	NRP2
cg24331301	chr10:73156165-73157954	CDH23
cg24347663	chr2:70994448-70995385	ADD2
cg24358846	chr8:99984585-99985072	
cg24368902	chr10:43248524-43250912	
cg24370475	chr11:69517841-69519929	FGF19
cg24382527	chr10:135089970-135090491	ADAM8
cg24385580	chr17:28088028-28088607	SSH2
cg24393316	chr9:100615235-100617510	FOXE1
cg24399349	chr7:55516872-55517249	
cg24399712	chr22:39784355-39785104	
cg24405179	chr1:226411008-226411880	MIXL1
cg24412501	chr11:13689589-13690724	FAR1; FAR1-IT1
cg24416513	chr2:176993480-176995557	HOXD8
cg24425838	chr1:151812254-151812525	C2CD4D
cg24432073	chr4:76555367-76556079	CDKL2
cg24437737	chr1:149399899-149400518	
cg24442454	chr6:37616723-37617179	MDGA1
cg24446586	chr7:27225051-27225629	HOXA11
cg24448269	chr17:54910497-54912470	DGKE; c17orf67
cg24453580	chr12:49688874-49691360	PRPH; AC125611.4
cg24468070	chr19:54974176-54976819	LENG9; CDC42EP5
cg24469980	chr1:221064890-221065600	
cg24471254	chr7:100253783-100254150	ACTL6B
cg24496475	chr4:1396292-1401730	NKX1-1
cg24505618	chr15:45421237-45422394	DUOXA1; DUOX1
cg24524285	chr11:64405452-64406006	NRXN2
cg24525176	chr1:47909713-47911020	
cg24525457	chr7:100091181-100091598	NYAP1
cg24542751	chr17:43339125-43339832	SPATA32; MAP3K14-AS1
cg24575083	chr11:7694712-7695685	CYB5R2
cg24588482	chr1:160370113-160370658	VANGL2
cg24589459	chr2:198649752-198651599	BOLL
cg24590788	chr13:95655109-95655517	
cg24599205	chr1:110752257-110754794	KCNC4
cg24599249	chr7:90893568-90896683	FZD1
cg24604013	chr13:112720565-112723582	SOX1
cg24610236	chr7:30721373-30722445	CRHR2
cg24626079	chr12:4378367-4382222	CCND2; CCND2-AS1
cg24626554	chr7:5111621-5112088	RBAKDN; RBAK-RBAKDN
cg24634568	chr1:43250763-43251082	TMEM269
cg24654525	chr12:58004983-58005351	ARHGEF25
cg24657817	chr4:42152767-42154984	BEND4

cg24659054	chr10:134597358-134602649	NKX6-2
cg24662718	chr1:108507252-108507841	VAV3; VAV3-AS1
cg24674635	chr20:6750696-6750957	BMP2
cg24683414	chr1:92945908-92952609	GFI1
cg24685006	chr1:36042433-36043444	TFAP2E
cg24710320	chr3:192958710-192959373	HRASLS
cg24714905	chr13:100637113-100637472	ZIC2
cg24718866	chr2:25391073-25391875	POMC
cg24718971	chr5:80255905-80257006	RASGRF2; RASGRF2-AS1
cg24722112	chr3:49755764-49757317	AMIGO3; GMPPB; RNF123
cg24724633	chr19:36909282-36909854	ZFP82
cg24724664	chr2:172944989-172945384	METAP1D
cg24740531	chr18:60985504-60985741	BCL2
cg24745495	chr19:15344092-15344419	EPHX3
cg24746100	chr2:88751336-88752865	FOXI3
cg24770624	chr2:20068429-20068907	LINC00954
cg24785067	chr18:19928417-19929329	
cg24794228	chr19:52390842-52391368	ZNF577; ZNF649-AS1
cg24800655	chr12:133065844-133066393	FBRSL1
cg24804544	chr7:6543796-6544108	GRID2IP
cg24808280	chr1:169396622-169396869	CCDC181
cg24809973	chr8:72468561-72469561	
cg24812837	chr10:102893661-102895059	TLX1
cg24816460	chr6:4775132-4777550	CDYL
cg24817154	chr6:27858167-27858564	HIST1H3J
cg24818939	chrX:101906002-101907017	ARMCX5-GPRASP2; GPRASP1
cg24831305	chr19:53426079-53426389	
cg24848035	chr8:101117923-101118693	RGS22
cg24851854	chr1:59042014-59043295	TACSTD2
cg24853724	chr7:28995306-28998541	TRIL
cg24856258	chr15:41952536-41953222	MGA
cg24876960	chr5:1881925-1887743	IRX4; CTD2194D22.4
cg24879782	chr2:19560964-19561650	
cg24880701	chr15:68115486-68122575	SKOR1
cg24884142	chr1:119531992-119532196	TBX15
cg24884703	chr1:91185157-91185577	
cg24886257	chr19:52452317-52452543	
cg24886267	chr5:167956220-167957371	FBLL1
cg24908814	chr5:132946745-132948467	FSTL4
cg24909548	chr19:36736022-36736884	ZNF565
cg24927800	chr2:220283201-220283750	DES
cg24929737	chr13:107186469-107189024	EFNB2
cg24932457	chr1:175568377-175568808	TNR
cg24934063	chr7:121943868-121944538	FEZF1; FEZF1-AS1
cg24937747	chr5:1881925-1887743	IRX4
cg24940138	chr17:41363728-41364273	TMEM106A
cg24947764	chr4:41881178-41881418	
cg24948962	chr18:2905951-2907348	EMILIN2
cg24954895	chr5:140800761-140801072	A 1-10; PCDHGA11; PCDHGA
cg24960947	chr11:65816405-65816665	GAL3ST3

cg24975222	chr3:45187027-45187946	CDCP1
cg24979233	chr4:1800145-1800351	FGFR3
cg24989962	chr14:52734208-52735486	PTGDR
cg24998096	chr5:115912002-115912384	SEMA6A; SEMA6-AS2
cg25001102	chr1:221068783-221069159	
cg25027788	chr12:6166874-6167258	VWF
cg25032595	chr13:96204692-96205496	CLDN10
cg25036779	chr6:1604607-1615866	FOXC1
cg25047056	chr10:30347600-30348416	JCAD
cg25057743	chr2:209271338-209271987	PTH2R
cg25062137	chr19:10589682-10590041	
cg25078444	chr14:29234890-29235908	FOXG1
cg25084878	chr5:127872564-127874945	FBN2
cg25087487	chr14:100625320-100626461	DEGS2
cg25088758	chr4:110222971-110224257	COL25A1
cg25109009	chr16:69760294-69760506	NQO1
cg25136495	chr12:6729249-6730310	LPAR5
cg25137711	chr1:48058794-48059230	
cg25147376	chr1:166889777-166890649	ILDR2
cg25152631	chr3:140660335-140661602	SLC25A36
cg25153755	chrX:47433415-47434124	SYN1
cg25174438	chr4:85403831-85404524	
cg25181651	chr2:1746834-1748971	PXDN
cg25181941	chr17:40805675-40805957	
cg25188395	chr7:27203916-27206462	HOXA9
cg25189564	chr7:158936508-158938492	VIPR2
cg25191628	chr1:50884229-50891471	DMRTA2
cg25209842	chr10:103535637-103536505	FGF8
cg25214789	chr5:7849946-7850439	c5orf49
cg25235766	chr19:54485304-54486322	CACNG8
cg25243455	chr2:114033360-114033617	PAX8; PAX8-AS1
cg25253217	chr19:47776371-47778740	CCDC9; INAFM1
cg25266629	chr10:102893661-102895059	TLX1
cg25287474	chr9:14693031-14693576	ZDHHC21
cg25295727	chr17:19483306-19483687	
cg25298725	chr6:30227321-30228255	HCG17
cg25307318	chr10:123922851-123923542	TACC2
cg25307665	chr7:27182614-27185562	HOXA5; HOXA6
cg25315816	chr12:96252021-96252246	SNRPF
cg25316339	chr5:79864843-79866447	ANKRD34B
cg25324047	chr10:22764709-22767050	
cg25324105	chr19:38182794-38183327	ZNF781
cg25330243	chr8:102504479-102504841	GRHL2
cg25330843	chr8:49293202-49293547	
cg25331703	chr15:101458287-101460223	LRRK1
cg25334393	chr8:145955640-145955989	ZNF251
cg25334575	chr13:100641335-100642188	
cg25335544	chr3:49906738-49907482	CAMKV
cg25340966	chr1:119531992-119532196	TBX15
cg25344734	chr11:13030331-13032584	RASSF10

cg25351606	chr6:100917206-100917523	
cg25363931	chr22:42896637-42897041	SERHL
cg25365934	chr10:77155129-77169600	ZNF503; ZNF503-AS2
cg25375340	chr1:85462586-85463435	MCOLN2
cg25392692	chr12:58021295-58022037	B4GALNT1
cg25397945	chr19:37095681-37096589	NF529, ZNF529-AS1; ZNF38
cg25416153	chr18:43355319-43355698	
cg25424856	chrX:50556225-50557179	SHROOM4
cg25466090	chr1:2064629-2064855	PRKCZ
cg25482900	chr5:167956220-167957371	FBLL1
cg25485875	chr10:134597358-134602649	NKX6-2
cg25486143	chr3:50377804-50378540	RASSF1
cg25503903	chr3:138679245-138679579	
cg25508679	chr6:28956107-28956578	
cg25513173	chr15:83875649-83877079	HDGFL3
cg25524962	chr13:20735044-20736157	GJA3
cg25527090	chr10:118893528-118894432	VAX1
cg25528916	chr19:4328659-4328865	STAP2
cg25537993	chr19:58545116-58545897	ZSCAN1
cg25552435	chr5:178016559-178017670	COL23A1
cg25577023	chr14:103394398-103397070	AMN
cg25585712	chr4:41869175-41869459	
cg25611476	chr5:88179867-88180258	MEF2C; MEFC2-AS1
cg25622366	chr2:63281035-63281347	OTX1
cg25623934	chr2:12856626-12859019	TRIB2
cg25627226	chr18:4453970-4455647	DLGAP1
cg25640635	chrX:100740222-100740601	
cg25644556	chr7:27208872-27209616	HOXA10-AS
cg25645687	chr3:138663719-138666346	FOXL2; FOXL2NB; LINC01391
cg25649641	chr15:76632263-76635515	ISL2
cg25657713	chr6:100038656-100039477	
cg25670330	chr3:138663719-138666346	FOXL2; FOXL2NB; LINC01391
cg25681339	chr17:1173536-1174733	BHLHA9
cg25693302	chr18:55862654-55862873	NEDD4L
cg25694349	chr12:133065844-133066393	FBRSL1
cg25714826	chr16:85783863-85785131	C16orf74
cg25720804	chr5:170735170-170739863	TLX3
cg25723050	chr1:224803638-224805314	CNIH3
cg25737323	chr2:29337984-29338909	CLIP4
cg25741023	chr10:102891011-102891794	TLX1
cg25742246	chr1:214153215-214153668	
cg25756406	chr10:134597358-134602649	NKX6-2
cg25756435	chr19:12952016-12952281	MAST1
cg25756780	chr2:121344551-121345341	
cg25757598	chr8:85096760-85097247	RALYL
cg25763393	chr19:52956657-52957245	ZNF578
cg25764899	chr1:47696505-47698197	TAL1
cg25771271	chr1:119549145-119551320	
cg25773262	chr1:156616554-156616946	BCAN
cg25784220	chr19:58609339-58609988	ZSCAN18

cg25792518	chr17:48545571-48546900	ACSF2; CHAD
cg25794766	chr1:247170869-247171434	ZNF695; ZNF670-ZNF695
cg25810938	chr2:241496577-241497600	ANKMY1
cg25823419	chr3:179168736-179169593	GNB4
cg25830696	chr17:42030174-42030941	PYY
cg25848557	chr1:108507252-108507841	VAV3; VAV3-AS1
cg25862444	chr1:147737016-147737303	NBPF8
cg25862644	chr10:77155129-77169600	ZNF503; ZNF503-AS2
cg25875213	chr19:38182794-38183327	ZNG781
cg25876509	chr7:97501184-97501919	ASNS
cg25881804	chr20:21080930-21082824	
cg25884711	chr7:24323559-24325080	NPY
cg25886284	chr19:36909282-36909854	ZFP82
cg25887069	chr22:38220590-38221619	GALR3
cg25900280	chr17:59531724-59535254	TBX4
cg25902187	chr15:55879793-55881228	PYGO1
cg25902889	chr19:4304543-4305122	FSD1
cg25920406	chr10:35928502-35931893	FZD8
cg25942450	chr5:170735170-170739863	TLX3
cg25945732	chr2:263401-265238	SH3YL1; ACP1
cg25947878	chr2:20068429-20068907	LINC00954
cg25951981	chr4:46995129-46995872	GABRA4
cg25963041	chr19:57049676-57050646	ZFP28; AC005498.2
cg25964032	chr10:35928502-35931893	FZD8
cg25970832	chr18:28621490-28623117	DSC3
cg26005578	chr11:13030331-13032584	RASSF10
cg26009486	chr14:24837873-24838324	NFATC4
cg26013553	chr1:111216245-111217937	KCNA3
cg26015401	chr19:49436696-49437055	DHDH
cg26029345	chr8:22960385-22960927	TNFRSF10C
cg26043257	chr4:15779999-15780729	CD38
cg26047066	chr1:221068783-221069159	
cg26057751	chr2:87015975-87018182	CD8A
cg26064774	chr4:6665672-6666089	
cg26071526	chr10:123922851-123923542	TACC2
cg26074603	chr12:75602992-75603344	KCNC2
cg26090107	chr11:60718429-60718888	SLC15A3
cg26103587	chr6:1619094-1621094	
cg26107850	chr1:228645197-228646434	HIST3H2A; HIST3H2BB
cg26107890	chr3:124860570-124861019	SLC12A8
cg26113809	chr12:54132149-54132353	
cg26114043	chr4:128544032-128544903	
cg26117023	chr2:74781495-74782685	DOK1; LOXL3
cg26121591	chr5:175298516-175300066	CPLX2
cg26124318	chr8:38965014-38965423	ADAM32
cg26128383	chr6:31238853-31240120	HLA-B; HLA-C
cg26130488	chr17:59528980-59530266	TBX4
cg26132320	chr20:44640289-44641210	MMP9
cg26142965	chr6:29521111-29521833	OR2I1P
cg26146617	chr2:232260101-232261134	B3GNT7

cg26149244	chr17:80329067-80330208	UTS2R
cg26156256	chr1:116370811-116371303	
cg26157385	chr1:248020331-248021252	TRIM58
cg26164269	chr14:62217726-62218077	
cg26173997	chr4:76555367-76556079	CDKL2
cg26195583	chr4:41869175-41869459	
cg26199241	chr17:59528980-59530266	TBX4
cg26211360	chr12:122667649-122668038	LRRC43
cg26240185	chr11:13689589-13690724	FAR1
cg26245531	chr14:88459119-88459689	GALC
cg26246928	chr1:153650831-153652375	NPR1
cg26248173	chr1:166853254-166853790	
cg26253438	chr1:203598472-203598853	ATP2B4
cg26256263	chr1:25255528-25259005	RUNX3
cg26259171	chr19:49255779-49256495	FUT1
cg26259537	chr7:27208872-27209616	HOXA10-AS
cg26261793	chr1:171810468-171811325	DNM3
cg26268044	chr10:97802872-97804262	CCNJ; ENTPD1-AS
cg26279550	chr12:66582696-66583345	IRAK3
cg26281453	chr17:45810590-45811388	TBX21
cg26292150	chr3:195934213-195934446	ZDHHC19
cg26297547	chr17:58227242-58227720	CA4
cg26301389	chr5:139047906-139048235	CXXC5
cg26303165	chr1:228399472-228402037	OBSCN; OBSCN-AS1
cg26315862	chr13:21520329-21520564	
cg26322231	chr7:138720045-138721019	ZC3HAV1L
cg26325335	chr3:50402104-50402942	CACNA2D2
cg26330116	chr15:37172474-37173018	
cg26333652	chr5:2748369-2757024	IRX2; c5orf38
cg26335633	chr7:64029903-64030972	
cg26349976	chr18:21718863-21719550	CABYR
cg26381352	chr6:33244678-33245554	B3GALT4
cg26381364	chr12:103889149-103889843	C12orf42
cg26386852	chr3:48693119-48694768	CELSR3
cg26388816	chr12:568703-570362	B4GALNT3
cg26397188	chr12:65514879-65515863	WIF1
cg26415547	chr12:66582696-66583345	IRAK3
cg26421310	chr1:25255528-25259005	RUNX3
cg26422488	chr5:16935556-16936408	MYO10
cg26425933	chr10:115999009-115999603	VWA2
cg26434983	chr15:89952272-89953061	
cg26444528	chr13:58206527-58208930	PCDH17
cg26447413	chr9:89560585-89562647	GAS1; GAS1RR
cg26448406	chr22:32026298-32026874	PISD
cg26460092	chr15:83775862-83776922	TM6SF1
cg26468007	chr7:4998406-4998950	MMD2
cg26468478	chr3:48693119-48694768	CELSR3
cg26470101	chr2:173099483-173100098	
cg26472636	chr5:1594239-1595027	SDHAP3
cg26476852	chr7:27203916-27206462	HOXA9

cg26477573	chr19:15342627-15343181	EPHX3
cg26482893	chrX:108867763-108868486	KCNE1L
cg26492368	chr10:22634001-22634862	SPAG6
cg26503073	chr7:6703504-6704075	
cg26506212	chrX:100740222-100740601	
cg26509691	chr5:134363093-134365146	PITX1
cg26517714	chr5:170735170-170739863	TLX3; AC091980.2
cg26521404	chr7:27203916-27206462	HOXA9
cg26526379	chr7:26415747-26416891	
cg26537209	chr16:54317822-54324604	IRX3
cg26540905	chr2:71693165-71694127	DYSF
cg26558485	chr1:47489227-47489633	CYP4X1
cg26560222	chr1:50884229-50891471	DMRTA2
cg26570179	chr8:99960498-99961438	OSR2
cg26575690	chr15:34728788-34729495	
cg26595643	chr10:118899248-118900329	VAX1
cg26599006	chr22:19136294-19138512	GSC2
cg26609631	chr13:28366550-28368505	GSX1
cg26612727	chr17:38024146-38024637	ZPBP2
cg26614816	chr12:104697349-104697984	EID3; TXNRD1
cg26618041	chr19:19651843-19652047	CILP2
cg26620530	chr12:115102688-115103982	
cg26654798	chr15:83951981-83953930	BNC1
cg26659805	chr19:2250561-2253959	JSRP1; AMH
cg26663651	chr7:139167481-139168712	KLRG2
cg26669806	chr19:18899038-18902284	COMP
cg26673012	chr7:35292986-35294674	TBX20
cg26674943	chr15:76627524-76627838	ISL2
cg26675876	chr8:11059012-11059242	XKR6
cg26680520	chr8:144822012-144822805	
cg26690949	chr20:58507756-58509517	FAM217B; SYCP2
cg26698460	chr19:58715221-58716033	ZNF274
cg26700716	chr2:183730809-183731357	FRZB
cg26709285	chr20:48098513-48099560	KCNB1
cg26714410	chr13:45149971-45152288	TSC22D1; TSC22D1-AS1
cg26715571	chr19:50554013-50554492	
cg26717554	chr19:852856-853495	ELANE
cg26727435	chr22:50453057-50454137	TTL8
cg26734888	chr19:37825102-37825756	HKR1
cg26735846	chr10:124894076-124897366	HMX3
cg26742995	chr17:43339125-43339832	SPATA32
cg26751792	chr19:15580383-15580863	PGLYRP2
cg26782108	chr17:6616423-6617471	SLC13A5
cg26790247	chr3:147108512-147111703	ZIC4
cg26797073	chr2:131129719-131130511	PTPN18
cg26802291	chr19:19371676-19372393	HAPLN4
cg26807961	chr19:47776371-47778740	INAFM1
cg26818735	chr7:19156051-19158042	TWIST1
cg26822097	chr22:17081937-17082595	TPTEP1
cg26833652	chr12:102036254-102036461	MYBPC1

cg26839871	chr1:171810468-171811325	DNM3
cg26844246	chr5:170735170-170739863	TLX3
cg26855801	chr2:45176729-45177264	
cg26857670	chr7:27225051-27225629	HOXA11-AS; HOXA11
cg26859365	chr12:52240773-52242547	
cg26859841	chr1:228744111-228784168	RHOU; DUSP5P1
cg26860935	chr4:1407447-1408014	
cg26870803	chr11:66623621-66626614	LRFN4; PC
cg26875805	chr1:166889777-166890649	ILDR2
cg26878949	chr9:98278129-98279754	PTCH1
cg26890189	chr19:47950870-47951566	SLC8A2
cg26915558	chr11:68181087-68181289	LRP5
cg26919818	chr5:177540208-177541234	N4BP3
cg26923490	chr19:49575130-49576076	KCNA7
cg26928125	chr11:69517841-69519929	FGF19
cg26931862	chr12:54348696-54349378	HOXC12
cg26946821	chr17:35291900-35300875	LHX1; LHX1-DT
cg26949694	chr11:27742060-27742273	BDNF
cg26953749	chr12:122667649-122668038	LRRC43
cg26954625	chr19:19738573-19739821	LAPAR2
cg26955987	chr12:3862069-3862606	CRACR2A; EFCAB4B
cg26958124	chr19:46032305-46032855	OPA3
cg26961808	chr7:128470444-128471161	FLCN
cg26966124	chr6:100912072-100913337	SIM1
cg26980244	chr8:24770909-24772547	NEFM
cg26983469	chr10:17270431-17272617	VIM; VIM-AS1
cg26990587	chr7:100253783-100254150	ACTL6B
cg26998044	chr17:8868470-8869372	PIK3R5
cg27005794	chr2:109744586-109746833	SH3RF3; SH3RF3-AS1
cg27009703	chr7:27203916-27206462	HOXA9
cg27011480	chr6:30227321-30228255	HCG17
cg27018380	chr14:93389246-93389899	CHGA
cg27021986	chr15:41803444-41805531	LTK
cg27028760	chr16:2041826-2043026	SYNGR3
cg27032146	chr7:96651964-96652246	DLX5
cg27041794	chr10:11059443-11060524	CELF2
cg27043726	chr8:101661672-101662022	SNX31
cg27045999	chr15:81426500-81426702	C15orf26
cg27049766	chr19:58220190-58220517	ZNF154; ZNF551
cg27051315	chr2:238864316-238865170	
cg27056599	chr17:21279507-21281574	KCNJ12
cg27067781	chr6:32116591-32117229	PRRT1
cg27071152	chr7:39649254-39649510	
cg27071460	chr5:40679503-40682081	PTGER4; TTC33
cg27076454	chr2:236578081-236580153	AGAP1
cg27085741	chr7:143042509-143042867	CLCN1
cg27090062	chr5:92939796-92940216	
cg27093143	chr19:36360265-36360835	APLP1
cg27111463	chr2:88751336-88752865	FOXI3
cg27114644	chr2:121344551-121345341	

cg27118059	chr21:32929928-32932017	TIAM1; AP000251.1
cg27127017	chr12:113592204-113592620	CCDC42B
cg27136844	chr3:147142182-147142391	
cg27142536	chr11:20618198-20619920	SLC6A5
cg27147819	chr20:21376359-21378245	NKX2-4
cg27166033	chr22:46929318-46934861	CELSR1
cg27177554	chr10:16561605-16563822	C1QL3
cg27212234	chr19:36048557-36049673	ATP4A
cg27226949	chr13:107186469-107189024	EFNB2
cg27232866	chr1:43250763-43251082	
cg27239157	chr3:183145428-183146790	MCF2L2
cg27252696	chr6:100912072-100913337	SIM1
cg27260772	chr6:50791111-50791573	TFAP2B
cg27262412	chr1:119529820-119530712	TBX15
cg27272677	chr10:102891011-102891794	TLX1; TLX1NB
cg27280575	chr20:44540446-44540957	PLTP
cg27282900	chr6:30095174-30095610	
cg27288226	chr19:57617602-57618448	
cg27304020	chr6:28956107-28956578	
cg27304406	chr15:83951981-83953930	BNC1
cg27306119	chr2:9143128-9144630	MBOAT2
cg27323784	chr11:116450660-116451912	
cg27331241	chr7:751713-752150	PRKAR1B
cg27353143	chr19:38307879-38308526	ZNF573
cg27356513	chr7:27265159-27265493	
cg27360326	chr13:53419898-53422872	PCDH8
cg27362525	chr17:5019638-5019879	ZNF232; USP6
cg27364741	chr2:63281035-63281347	OTX1
cg27376182	chr16:3232836-3234048	
cg27382405	chr12:49942787-49943435	KCNH3
cg27392792	chr7:158379329-158381221	PTPRN2
cg27393010	chr7:121940007-121940648	FEZF1
cg27395939	chr11:17373020-17373665	NCR3LG1
cg27404023	chr2:162283582-162284677	TBR1
cg27405791	chr4:41882451-41882964	
cg27417609	chr16:68676451-68676843	CDH3
cg27424995	chr1:27901661-27902688	AHDC1
cg27434368	chr19:19650684-19651274	CILP2
cg27452341	chr7:143042509-143042867	CLCN1
cg27454064	chr12:64215500-64215990	
cg27456468	chr2:200333688-200334172	STAB2
cg27478242	chr12:54611596-54612038	
cg27478313	chr6:1624186-1625468	GMDS
cg27496526	chr15:41803444-41805531	LTK
cg27504802	chr1:231297813-231299777	TRIM67
cg27505047	chrX:133683175-133685463	LINC00629
cg27506082	chr2:207139337-207140031	ZDBF2
cg27507687	chr3:48631883-48632901	COL7A1
cg27513979	chr13:96742139-96744162	HS6ST3
cg27522060	chr15:65360025-65360542	RASL12

cg27537203	chr1:38510103-38513642	POU3F1
cg27549878	chr19:38886110-38886615	SPRED3
cg27552679	chr15:74425092-74428821	ISLR2
cg27558479	chr2:74425445-74426423	MTHFD2
cg27574000	chr14:36002599-36005013	INSM2
cg27578811	chr15:83315117-83317541	CPEB1; CPEB1-AS1
cg27587125	chr1:109203594-109204378	HENMT1
cg27595860	chr8:145555343-145562310	SCRT1
cg27605748	chr5:42951077-42952410	
cg27616541	chr17:40837023-40837525	CNTNAP1
cg27633530	chr7:50343758-50344519	IKZF1
cg27636310	chr1:46632690-46632932	PIK3R3
cg27649073	chr10:104000255-104001741	PITX3
cg27649239	chr15:68115486-68122575	SKOR1
cg27655158	chr4:1396292-1401730	
cg27663938	chr12:103696091-103696418	c12orf42

Table S2

CpG site (hg19)_Illumina 450K Array	CGI (hg19)	Gene name
cg00029353	chr12:52240773-52242547	
cg00030422	chr3:38080629-38081187	DLEC1
cg00108164	chr2:263401-265238	SH3YL1
cg00114029	chr1:35350879-35351854	DLGAP3
cg00117172	chr1:25255528-25259005	RUNX3
cg00121634	chr3:169529876-169530435	LRRC34
cg00121843	chr13:33923931-33924781	STARD13
cg00133595	chr16:31225957-31228264	TRIM72
cg00155526	chr5:32709911-32714450	NPR3
cg00157796	chr6:159589637-159591319	
cg00164949	chr17:43037167-43037740	C1QL1
cg00292664	chr10:71077947-71079377	HK1
cg00370229	chr6:29521111-29521833	
cg00393798	chr11:8615398-8615889	STK33
cg00456086	chr3:197281606-197283128	BDH1
cg00527440	chr2:242549374-242549995	THAP4
cg00544436	chr6:34202568-34206193	HMGA1
cg00556112	chr11:636907-640628	DRD4
cg00584713	chr1:213123648-213125092	VASH2
cg00599393	chr8:22457124-22457753	C8orf58
cg00612595	chr21:47717236-47718059	YBEY
cg00626110	chr10:134597358-134602649	NKX6-2
cg00648301	chr20:20344401-20350605	INSM1
cg00723271	chr14:102247661-102248279	PPP2R5C
cg00731785	chr4:15704641-15705000	BST1
cg00753478	chr12:21810489-21810766	LDHB
cg00773413	chr10:77155129-77169600	ZNF5 3-AS2
cg00803088	chr10:43600147-43601165	RET
cg00806198	chr17:72968168-72969164	HID1
cg00817367	chr12:52400468-52401696	GRASP
cg00864183	chr17:48045789-48047207	DLX4
cg00896540	chr8:82192379-82193685	FABP5
cg00909646	chr8:67873389-67875600	TCF24
cg00914041	chr8:91997231-91997906	LOC1 127983
cg00922376	chr1:14026482-14027200	PRDM2
cg00971050	chr17:40573741-40575526	PTRF
cg00995327	chr3:142837886-142840838	CHST2
cg01009697	chr9:87283179-87285704	NTRK2
cg01060026	chr3:129323916-129325332	PLXND1
cg01076997	chr22:37815804-37816477	ELFN2
cg01108118	chr6:391189-393790	IRF4
cg01112965	chr19:38852312-38853485	CATSPERG
cg01143804	chr4:40751843-40752493	NSUN7
cg01163842	chr14:95234613-95236569	GSC
cg01246835	chr7:6703504-6704075	AK1233
cg01268683	chr19:53141176-53141813	ZNF83

cg01373292	chr1:7764594-7765856	CAMTA1
cg01419831	chr2:162283582-162284677	
cg01420426	chr1:41831977-41832542	
cg01423964	chr1:111216245-111217937	KCNA3
cg01445942	chr7:72847934-72850032	FZD9
cg01485998	chr1:213123648-213125092	VASH2
cg01490894	chr6:26550783-26551114	
cg01535733	chr6:153450993-153452792	RGS17
cg01556502	chr14:103655242-103655928	LINC 6 5
cg01565320	chr4:42399153-42400802	SHISA3
cg01579950	chr11:64480200-64481344	NRXN2
cg01627847	chr17:43339125-43339832	SPATA32; MAP3K14-AS1
cg01645753	chr15:83315117-83317541	CPEB1
cg01649597	chr2:232394764-232395337	NMUR1
cg01696193	chr6:56818874-56820308	BEND6
cg01718447	chr7:30721373-30722445	CRHR2
cg01720033	chr17:62774367-62777797	BPTFP1-KPNA2P3; ARHGAP2[illegible]
cg01725199	chr9:101569906-101570349	GALNT12
cg01765249	chr17:59473061-59483266	TBX2
cg01824969	chr8:67873389-67875600	TCF24
cg01833196	chr10:105452339-105453230	SH3PXD2A
cg01851088	chr3:48698336-48701667	CELSR3
cg01901107	chr7:150747977-150748519	ASIC3
cg01913455	chr17:59473061-59483266	TBX2
cg01939477	chr11:43602546-43603215	MIR129-2
cg01981601	chr8:60032533-60032746	
cg01992990	chr1:202779791-202780684	KDM5B-AS1
cg01995480	chr22:45403037-45406372	PHF21B
cg01996714	chr20:3218579-3220930	SLC4A11
cg02009088	chr5:139227606-139228279	NRG2
cg02011074	chr1:171810468-171811325	DNM3
cg02017200	chr19:49255779-49256495	FUT1
cg02096396	chr2:26395417-26395620	GAREML
cg02119927	chr10:71077947-71079377	HK1
cg02132470	chr7:98467024-98468058	TMEM13
cg02147764	chr2:45169506-45171884	SIX3
cg02215070	chr7:134143116-134144063	AKR1B1
cg02227867	chr8:22457124-22457753	C8orf58
cg02325540	chr10:94833273-94835256	CYP26A1
cg02330121	chr6:118228043-118229811	SLC35F1
cg02337836	chr1:110210582-110210956	GSTM2
cg02349096	chr19:11450017-11450414	RAB3D
cg02362385	chr19:19738573-19739821	LPAR2
cg02396496	chr17:74533282-74534566	PRCD; CYGB
cg02408775	chr12:48206722-48207126	HDAC7
cg02433564	chr6:37673309-37673774	
cg02434443	chr11:15136059-15136545	INSC
cg02457680	chr1:154474108-154475699	TDRD1
cg02466113	chr4:76555367-76556079	CDKL2
cg02511231	chr5:170735170-170739863	TLX3

cg02553663	chr17:80291021-80292394	SECTM1
cg02578368	chr6:26250437-26250827	HIST1H3F
cg02586551	chr11:73693703-73694802	UCP2
cg02590401	chr19:48983563-48984175	CYTH2
cg02636041	chr10:43697778-43698177	RASGEF1A
cg02659086	chr11:67350929-67351953	GSTP1
cg02659794	chr12:21810489-21810766	LDHB
cg02668581	chr2:216877900-216878352	MREG
cg02675308	chr4:76555367-76556079	CDKL2
cg02685896	chr17:27038085-27038919	PROCA1
cg02699694	chr2:9143128-9144630	MBOAT2
cg02716516	chr3:45837481-45838256	SLC6A2
cg02746869	chr10:17270431-17272617	VIM
cg02788401	chr17:48585386-48586167	MYCBPAP
cg02831587	chr11:13689589-13690724	FAR1
cg02858118	chr3:48693119-48694768	CELSR3
cg02884176	chr2:88751336-88752865	FOXI3
cg02884239	chr17:75368689-75370506	SEPTIN9
cg02886408	chr10:133794899-133796598	BNIP3
cg02909790	chr6:26271346-26271588	HIST1H3G
cg02912326	chr6:97284831-97285782	GPR63
cg02919936	chr8:70981874-70984888	PRDM14
cg03024619	chr3:71802561-71804209	EIF4E3
cg03048083	chr17:43339125-43339832	SPATA32; MAP3K14-AS1
cg03063639	chr15:83775862-83776922	TM6SF1
cg03070194	chr1:110210582-110210956	GSTM2
cg03073402	chr19:42927286-42928598	LIPE
cg03109306	chr11:123524734-123525685	SCN3B
cg03113878	chr7:100091181-100091598	NYAP1
cg03200052	chr1:223536843-223538628	SUSD4
cg03208016	chr1:180123556-180124584	QSOX1
cg03217795	chr16:23846942-23848102	PRKCB
cg03306374	chr16:23846942-23848102	PRKCB
cg03308628	chr12:95941907-95942979	USP44
cg03309253	chr19:41119032-41120394	LTBP4
cg03321592	chr1:19991147-19992788	HTR6
cg03326059	chr11:13689589-13690724	FAR1
cg03410231	chr7:143042509-143042867	CLCN1
cg03423149	chr11:63766059-63768666	MACROD1
cg03444245	chr11:45376932-45377243	LOC1 1928812
cg03455458	chr1:213123648-213125092	VASH2
cg03468349	chr2:263401-265238	SH3YL1
cg03506033	chr11:75378417-75379882	MAP6
cg03509671	chr3:101497831-101498648	NXPE3
cg03517376	chr16:30389036-30390631	MYLPF
cg03585419	chr19:54369388-54369809	MYADM
cg03607359	chr5:176056521-176057494	SNCB
cg03612722	chr22:27053082-27053629	MIAT
cg03666741	chr11:9025096-9026315	NRIP3
cg03671191	chr19:52207133-52207731	SPACA6

cg03695666	chr19:38210108-38210692	ZNF6 7
cg03705926	chr19:6740670-6741203	TRIP1
cg03710719	chr19:36389546-36389967	NFKBID
cg03720581	chr11:506354-508009	RNH1
cg03755177	chr7:44349112-44349716	CAMK2B
cg03766620	chr2:127783082-127783311	
cg03768777	chr1:213123648-213125092	VASH2
cg03776464	chr2:222436035-222438941	EPHA4
cg03780132	chr5:42951077-42952410	
cg03786933	chr16:69760294-69760506	NQO1
cg03787864	chr16:88716990-88717606	CYBA
cg03803071	chr19:22034395-22034943	ZNF43
cg03804136	chr17:75368689-75370506	SEPTIN9
cg03825010	chr5:159399005-159399928	ADRA1B
cg03848381	chr15:40573629-40576118	ANKRD63
cg03864000	chr11:111410933-111412199	LAYN
cg03885037	chr10:135089970-135090491	ADAM8
cg03932723	chr11:63258379-63258804	HRASLS5
cg03957481	chr22:50984973-50988141	KLHDC7B
cg03976877	chr7:158936508-158938492	VIPR2
cg04008888	chr11:68622723-68623252	
cg04012924	chr11:13689589-13690724	FAR1
cg04035559	chr10:71077947-71079377	HK1
cg04085025	chr7:30028519-30029822	SCRN1
cg04093225	chr1:166916866-166917215	ILRD2
cg04100696	chr11:12029738-12030841	DKK3
cg04111064	chr3:138663719-138666346	FOXL2; FOXL2NB
cg04147049	chr1:33219428-33220028	KIAA1522
cg04148762	chr1:203598472-203598853	ATP2B4
cg04203238	chr4:16084196-16085735	PROM1
cg04209650	chr17:19648140-19648492	ALDH3A1
cg04221886	chr2:44058766-44059232	ABCG5
cg04246763	chr1:87617693-87617986	LINC 114
cg04281464	chr14:70014544-70014993	
cg04293902	chr10:134597358-134602649	NKX6-2
cg04321866	chr13:107186469-107189024	EFNB2
cg04361852	chr11:73693703-73694802	UCP2
cg04365224	chr3:72788073-72788416	
cg04395369	chr19:19738573-19739821	LPAR2
cg04407470	chr6:108485672-108490539	NR2E1
cg04426311	chr3:3840514-3842772	LRRN1
cg04487931	chr1:28199032-28199257	THEMIS2
cg04497176	chr19:34972055-34973645	WTIP
cg04502985	chr5:77268351-77268787	
cg04515996	chr6:85472703-85474132	TBX18
cg04550737	chr1:119529820-119530712	TBX15
cg04557125	chr4:41883493-41884570	LINC 682
cg04564920	chr3:122296613-122296828	PARP15
cg04618002	chr7:5632336-5634555	FSCN1
cg04638468	chr4:122685861-122686565	TMEM155

cg04652621	chr7:150671807-150672370	KCNH2
cg04673590	chr7:27135343-27136736	HOTAIRM1
cg04743654	chr3:50377804-50378540	RASSF1
cg04779720	chr1:11919116-11919555	
cg04781274	chr16:67034471-67035340	CES4A
cg04787024	chr2:99552186-99553831	KIAA1211L
cg04805619	chr3:167097928-167098198	ZBBX
cg04823311	chr7:28995306-28998541	TRIL
cg04907523	chr1:213123648-213125092	VASH2
cg04920358	chr8:49468684-49468959	LOC1 1929268
cg04920951	chr11:67350929-67351953	GSTP1
cg04970117	chr3:45837481-45838256	SLC6A2
cg04981611	chr2:47796924-47799166	MSH2
cg04992638	chr17:43339125-43339832	SPATA32; MAP3K14-AS1
cg05040544	chr13:107186469-107189024	EFNB2
cg05095158	chr11:13030331-13032584	RASSF1
cg05099508	chr10:22634001-22634862	SPAG6
cg05106897	chr6:34433412-34434371	PACSIN1
cg05184938	chr17:75368689-75370506	SEPTIN9
cg05297854	chr22:50623165-50625226	TRABD
cg05309948	chr11:2290105-2292932	
cg05327835	chr4:995483-997541	IDUA
cg05337743	chr19:54369388-54369809	MYADM
cg05346841	chr1:14026482-14027200	PRDM2
cg05347898	chr6:24357720-24358309	DCDC2; KAAG1
cg05377226	chr1:171810468-171811325	DNM3
cg05386493	chr3:138663719-138666346	FOXL2; FOXL2NB
cg05457344	chr6:26251899-26252233	HIST1H2BH
cg05488523	chr17:79478236-79482426	ACTG1
cg05495949	chr7:4922708-4923576	RADIL
cg05519781	chr21:40032244-40033665	ERG
cg05529506	chr18:44526867-44527137	KATNAL2
cg05545132	chr2:144694667-144695180	LOC1 1928386
cg05554936	chr19:11450017-11450414	RAB3D
cg05569109	chr2:166649910-166650966	GALNT3
cg05617413	chr12:133065844-133066393	
cg05629186	chr6:27356493-27357020	ZNF391
cg05639679	chr1:33219428-33220028	KIAA1522
cg05663341	chr7:96634623-96634915	DLX6-AS1
cg05684891	chr9:124460968-124461361	DAB2IP
cg05714219	chr2:31360322-31361821	GALNT14
cg05720261	chr1:40105011-40105707	HEYL
cg05721365	chr17:62774367-62777797	3PTFP1-KPNA2P3; ARHGAP2
cg05739190	chr10:97802872-97804262	CCNJ; ENTPD1-AS1
cg05751100	chr6:26550783-26551114	
cg05766140	chr6:391189-393790	IRF4
cg05770238	chr1:26686517-26687281	
cg05843841	chr11:76381450-76382295	LRRC32
cg05871997	chr6:56818874-56820308	DST; BEND6
cg05933935	chr1:6051596-6052955	NPHP4; KCNAB2

cg05994148	chr6:34433412-34434371	PACSIN1
cg06036912	chr16:3067522-3068358	CLDN6
cg06040206	chr2:172959268-172962054	
cg06048524	chr10:44879715-44882391	CXCL12
cg06073471	chr3:101497831-101498648	NXPE3
cg06186698	chr3:138663719-138666346	FOXL2NB; FOXL2
cg06196147	chr3:128712405-128713058	KIAA1257
cg06211893	chr1:171810468-171811325	DNM3
cg06215569	chr1:110610266-110613303	ALX3
cg06223767	chr6:391189-393790	IRF4
cg06241292	chr3:49314438-49314815	USP4
cg06270244	chr15:78933188-78933821	CHRNB4
cg06341513	chr11:61275826-61277329	LRRC1 B
cg06366062	chr17:30813178-30815239	CDK5R1
cg06377278	chr1:25255528-25259005	RUNX3
cg06396724	chr14:61188117-61190772	SIX4
cg06399148	chr8:104152727-104153390	BAALC
cg06431702	chr11:66623621-66626614	LRFN4; PC
cg06463958	chr6:166579974-166583423	TBXT
cg06472476	chr19:54369388-54369809	MYADM
cg06611810	chr19:41316544-41317318	CYP2T1P
cg06665109	chr19:54369388-54369809	MYADM
cg06686029	chr20:30777757-30778329	TSPY26P
cg06741043	chr15:68870634-68871974	CORO2B
cg06749789	chr2:242549374-242549995	THAP4
cg06782692	chr10:134120448-134122422	STK32C
cg06790860	chr16:69760294-69760506	NQO1
cg06825142	chr11:636907-640628	DRD4
cg06848185	chr17:75368689-75370506	SEPTIN9
cg06893138	chr1:228645197-228646434	HIST3H2A
cg06897264	chr6:28979247-28979499	
cg06905726	chr19:18209912-18210268	MAST3
cg06913330	chr4:2059929-2063181	NAT8L
cg06921552	chr11:69258150-69258544	
cg06933574	chr5:134368579-134370466	PITX1
cg06947913	chr12:50297581-50297988	FAIM2
cg06992285	chr8:99952021-99954686	STK3
cg07036412	chr6:24360030-24360411	DCDC2
cg07044708	chr15:75470912-75471212	
cg07049552	chr16:30204696-30206485	SLX1A
cg07074543	chr3:119041383-119042255	ARHGAP31
cg07076076	chr16:1458144-1458435	UNKL
cg07095997	chr14:61188117-61190772	SIX4
cg07137955	chr5:42992034-42992955	AK 56817
cg07138355	chr6:26550783-26551114	
cg07167423	chr1:36042433-36043444	TFAP2E
cg07219542	chr1:110210582-110210956	GSTM2
cg07230107	chr1:224803638-224805314	CNIH2
cg07276415	chr11:46354092-46355190	DGKZ
cg07352345	chr5:175792356-175793505	ARL1

cg07380496	chr5:71402878-71404868	MAP1B
cg07399369	chr3:142837886-142840838	CHST2
cg07435294	chr11:65553750-65555573	OVOL1
cg07451080	chr3:44036408-44041104	
cg07513622	chr7:18125915-18127009	HDAC9
cg07528613	chr19:53073309-53074039	ZNF7 1
cg07561017	chr12:48397890-48398731	COL2A1
cg07589773	chr7:50343758-50344519	IKZF1
cg07622404	chr3:101497831-101498648	NXPE3
cg07688604	chr12:133065844-133066393	
cg07689503	chr2:74425445-74426423	MTHFD2
cg07702750	chr7:28995306-28998541	TRIL
cg07791578	chr6:24357720-24358309	DCDC2
cg07799366	chr6:52226754-52228006	PAQR8
cg07844931	chr16:58018401-58019471	TEPP
cg07846311	chr4:57371583-57372022	ARL9
cg07878486	chr19:58951215-58952250	ZNF132
cg07879739	chr22:38220590-38221619	GALR3
cg07888856	chr6:137242316-137245442	SLC35D3
cg07960067	chr2:9143128-9144630	MBOAT2
cg07987843	chr12:3308813-3310270	TSPAN9
cg07993547	chr19:18209912-18210268	MAST3
cg08034379	chr2:228736231-228736544	DAW1
cg08042316	chr7:139930066-139930356	
cg08081805	chr19:49242019-49242962	RASIP1
cg08100565	chr3:140660335-140661602	SLC25A36
cg08101407	chr20:30777757-30778329	TSYP26P
cg08173692	chr6:166720240-166722275	PRR18
cg08187418	chr2:74425445-74426423	MTHFD2
cg08196842	chr14:65016517-65016909	PPP1R36
cg08226111	chr14:36002599-36005013	INSM2
cg08228715	chr17:79894669-79895533	PYCR1
cg08247376	chr17:43339125-43339832	SPATA32; MAP3K14-AS1
cg08249424	chr6:4775132-4777550	CDYL
cg08296680	chr17:80186136-80189990	SLC16A3
cg08296831	chr11:65553750-65555573	OVOL1
cg08303146	chr11:2466248-2466818	KCNQ1
cg08347500	chr16:54315603-54316104	
cg08407114	chr5:36690208-36690658	
cg08444220	chr10:134597358-134602649	NKX6-2
cg08452658	chr15:83775862-83776922	TM6SF1
cg08460435	chr1:109203594-109204378	HENMT1
cg08465862	chr10:3108651-3111419	PFKP
cg08482682	chr11:107798959-107799980	RAB39A
cg08494905	chr6:37663762-37667102	MDGA1
cg08495115	chr15:33009531-33011696	GREM1
cg08513472	chr6:1624186-1625468	GMDS
cg08553437	chr4:122685861-122686565	TMEM155
cg08682544	chr16:66638255-66639561	CMTM3
cg08694014	chr16:68480865-68482822	SMPD3

cg08741214	chr2:74425445-74426423	MTHFD2
cg08763705	chr5:139525538-139526227	
cg08789053	chr6:28979247-28979499	
cg08844848	chr3:3840514-3842772	LRRN1
cg08848774	chr17:43047548-43047954	
cg08854306	chr4:91048412-91049682	CCSER1
cg08858437	chr3:142837886-142840838	CHST2
cg08862890	chr5:169064271-169064702	DOCK2
cg08892600	chr1:223536843-223538628	SUSD4
cg08892705	chr15:99645031-99646444	SYNM
cg08934846	chr16:3067522-3068358	CLDN6
cg08940787	chr17:62774367-62777797	[illegible]PTFP1-KPNA2P3 y ARHGAP2[illegible]
cg08956724	chr16:29624388-29625391	SMG1P2
cg09118312	chr12:21810489-21810766	LDHB
cg09163958	chr1:213123648-213125092	VASH2
cg09193347	chr15:79381805-79384209	RASGRF1
cg09200738	chr15:30864783-30865586	ULK4P2
cg09233013	chr16:66638255-66639561	CMTM3
cg09241929	chr20:57463653-57467739	GNAS
cg09248054	chr1:967967-970238	AGRN
cg09252528	chr19:42901017-42901375	LIPE-AS1
cg09392940	chr2:109744586-109746833	SH3RF3; SH3RF3-AS1
cg09418984	chr19:54369388-54369809	MYADM
cg09420439	chr7:27135343-27136736	HOTAIRM1
cg09433135	chr19:46001831-46002686	PPM1N
cg09472203	chr15:83378008-83379068	AP3B2
cg09614653	chr10:22540708-22542739	LOC1 13 992
cg09616463	chr15:72611947-72612802	CELF6
cg09642925	chr2:114260096-114261794	
cg09652652	chr3:194406441-194409045	FAM43A
cg09654997	chr11:46259265-46260048	
cg09661010	chr10:73846807-73848233	SPOCK2
cg09691340	chr17:42402788-42403266	
cg09695735	chr17:58498712-58499332	
cg09761040	chr19:13208344-13210525	LYL1; NFIX
cg09775582	chr17:45810590-45811388	TBX21
cg09793584	chr1:156390404-156391581	C1orf61
cg09848789	chr1:85462586-85463435	MCOLN2
cg09866102	chr16:31213567-31214287	PYCARD-AS1; PYCARD
cg09868451	chr2:74425445-74426423	MTHFD2
cg09871079	chr1:33358470-33359449	HPCA
cg09894698	chr11:2290105-2292932	ASCL2
cg09938462	chr22:19705511-19706557	SEPTIN5; SEPT5-GP1BB
cg09952395	chr11:63258379-63258804	HRASLS5
cg09992116	chr10:71077947-71079377	HK1
cg10023272	chr1:110610266-110613303	ALX3
cg10034364	chr13:28491489-28492518	
cg10064339	chr11:73693703-73694802	UCP2
cg10091994	chr12:4378367-4382222	CCND2; CCND2-AS1
cg10106804	chr1:35258565-35258791	GJA4

cg10122865	chr2:63283937-63284147	OTX1
cg10154122	chr19:38754847-38755699	SPINT2
cg10162691	chr17:41177337-41177593	RND2
cg10181419	chr2:263401-265238	chr2:2634 1-265238
cg10191210	chr5:42992034-42992955	
cg10297491	chr10:11059443-11060524	CELF2
cg10304922	chr12:133065844-133066393	
cg10333808	chr12:22486836-22488666	ST8SIA1
cg10334767	chr4:10458130-10459353	ZNF518B
cg10344081	chr4:76555367-76556079	CDKL2
cg10363661	chr16:89641054-89643415	CPNE7
cg10376598	chr19:19738573-19739821	LPAR2
cg10379890	chr6:391189-393790	IRF4
cg10386298	chr1:221050449-221050864	HLX-AS1
cg10429922	chr15:33009531-33011696	GREM1
cg10480343	chr15:48936811-48938577	FBN1
cg10511618	chr4:113435899-113438002	NEUROG2; NEUROG2-AS1
cg10523966	chr1:39956425-39958137	BMP8A
cg10555583	chr20:1783825-1784552	
cg10572969	chr11:13689589-13690724	FAR1; FAR1-IT1
cg10573386	chr3:48698336-48701667	CELSR3
cg10611186	chr17:30813178-30815239	CDK5R1
cg10623840	chr3:194406441-194409045	FAM43A
cg10633601	chr1:47696505-47698197	TAL1
cg10637512	chr15:99645031-99646444	SYNM
cg10695848	chr12:48206722-48207126	HDAC7
cg10715425	chr1:6051596-6052955	NPHP4; KCNAB2
cg10716835	chr10:94833273-94835256	CYP26A1
cg10723962	chr6:26240698-26240951	H4C6; HIST1H4F
cg10800464	chr12:109162409-109162722	
cg10886442	chr3:142837886-142840838	CHST2
cg10900437	chr6:149068180-149069775	UST
cg10904986	chr8:145555343-145562310	SCRT1
cg10979880	chr6:105584149-105585621	BVES; BVES-AS1
cg11012584	chr2:242549374-242549995	THAP4
cg11068343	chr6:24360030-24360411	DCDC2
cg11073571	chr4:78978366-78979256	FRAS1
cg11172693	chr20:37434207-37435592	PPP1R16B
cg11173146	chr13:107186469-107189024	EFNB2
cg11183365	chr6:27064629-27065004	
cg11198128	chr11:65601070-65601551	SNX32
cg11223367	chr3:101497831-101498648	NXPE3
cg11235741	chr15:82335473-82339937	MEX3B
cg11258043	chr6:73972820-73973027	KHDC1
cg11279444	chr1:53098630-53099303	SHISAL2A
cg11364273	chr11:63766059-63768666	MACROD1
cg11376305	chr19:49127389-49127720	SPHK2
cg11398523	chr19:50316212-50316469	FUZ
cg11459603	chr6:26273291-26273557	HIST1H2BI; H2BC1
cg11472279	chr10:11059443-11060524	CELF2

cg11474250	chr5:107005131-107008191	EFNA5
cg11481534	chr6:116691828-116692868	DSE
cg11485595	chr5:32709911-32714450	NPR3
cg11500797	chr7:96651964-96652246	DLX5
cg11502745	chr11:18742691-18743513	IGSF22
cg11509907	chr2:235404503-235406541	ARL4C
cg11510060	chr4:330163-332068	ZNF141
cg11521404	chr13:20735044-20736157	GJA3
cg11549953	chr5:43017969-43018668	
cg11595545	chr1:111216245-111217937	KCNA3
cg11600807	chr11:17373020-17373665	NCR3LG1
cg11660826	chr6:150284683-150286515	ULBP1
cg11672054	chr1:228645197-228646434	HIST3H2A; HIST3H2BB
cg11684022	chr1:247494250-247495840	ZNF496
cg11686528	chr17:1082377-1083833	ABR
cg11694752	chr11:75378417-75379882	MAP6
cg11716665	chr5:149569593-149569945	SLC6A7
cg11721803	chr12:48397890-48398731	COL2A1
cg11737742	chr1:110752257-110754794	KCNC4
cg11804775	chr19:19729128-19729814	PBX4
cg11891395	chr7:96651964-96652246	DLX5
cg11929643	chr3:48698336-48701667	CELSR3
cg11940285	chr3:129693128-129694841	TRH
cg11973177	chr10:17270431-17272617	VIM; VIM-AS1
cg12002882	chr20:62461267-62462037	ZBTB46
cg12022772	chr2:131129719-131130511	PTPN18
cg12035092	chr2:149632683-149633882	KIF5C
cg12040486	chr19:11959578-11960064	ZNF439
cg12041056	chr22:27053082-27053629	MIAT
cg12078320	chr1:54940347-54940753	
cg12086936	chr11:31831621-31839038	PAX6
cg12091732	chr10:82116203-82117120	DYDC2
cg12177220	chr3:38080629-38081187	DLEC1
cg12178578	chr1:45792420-45793301	HPDL
cg12191938	chr10:88730555-88731632	AGAP11; ADIRF
cg12212657	chr3:3840514-3842772	LRRN1
cg12220691	chr19:41641308-41642082	
cg12269002	chr11:77122737-77123088	PAK1
cg12281657	chr14:65016517-65016909	PPP1R36
cg12319602	chr5:43017969-43018668	
cg12414653	chr12:8123347-8123561	
cg12457909	chr7:39649254-39649510	YAE1
cg12477533	chr2:98703355-98703889	VWA3B
cg12518410	chr2:109744586-109746833	SH3RF3; SH3RF3-AS1
cg12539796	chr5:7849946-7850439	c5orf49
cg12631351	chr5:80255905-80257006	RASGRF2; RASGRF2-AS1
cg12668309	chr3:53527928-53530106	CACNA1D
cg12690148	chr1:63782395-63790471	FOXD3; FOXD3-AS1
cg12727374	chr3:183542497-183543804	MAP6D1
cg12796229	chr18:12407716-12408242	SLMO1

cg12825804	chr14:77227570-77228691	VASH1
cg12863545	chr17:8868470-8869372	PIK3R5
cg12927498	chr5:141030919-141031177	FCHSD1
cg12928379	chr11:636907-640628	DRD4
cg12936335	chr6:150284683-150286515	ULBP1
cg12992720	chr19:19738573-19739821	LAPAR2
cg12998491	chr9:134151854-134153015	FAM78A
cg13001868	chr17:43339125-43339832	SPATA32; MAP3K14-AS1
cg13056495	chr7:134143116-134144063	AKR1B1
cg13066043	chr14:24808636-24808992	RIPK3
cg13088368	chr20:42543098-42545137	TOX2
cg13092806	chr2:177042752-177043444	HAGLR; HAGLROS
cg13111733	chr1:228645197-228646434	HIST3H2BB; HIST3H2A
cg13118906	chr17:61523001-61524470	CYB561
cg13177786	chr11:10472001-10472857	AMPD3
cg13204512	chr17:29298047-29298606	RNF135
cg13258989	chr17:72968168-72969164	HID1
cg13267747	chr5:141030919-141031177	FCHSD1
cg13280283	chr6:137242316-137245442	SLC35D3
cg13304665	chr22:22862624-22863220	ZNF28 B
cg13323701	chr6:118228043-118229811	SLC35F1
cg13325154	chr10:104000255-104001741	PITX3
cg13326227	chr20:20344401-20350605	INSM1
cg13341720	chr19:6740670-6741203	TRIP1
cg13355047	chr19:4304543-4305122	FSD1
cg13438961	chr1:41249091-41250161	KCNQ4
cg13476854	chr1:119549145-119551320	
cg13516820	chr9:37034136-37038341	PAX5
cg13556491	chr19:19738573-19739821	LAPAR2
cg13570982	chr14:55595698-55596692	LGALS3
cg13592399	chr14:52534582-52536722	NID2
cg13662093	chr20:33865334-33865563	MP24OS; MMP24-AS1-EDEM
cg13699414	chr5:178957434-178957900	
cg13702005	chr12:103889149-103889843	C12orf42
cg13717446	chr17:62774367-62777797	27P1-BPTFP1-KPNA2P3; ARH
cg13725782	chr6:105584149-105585621	BVES; BVES-AS1
cg13729891	chr17:7108305-7108654	DLG4
cg13730743	chr19:52207133-52207731	SPACA6
cg13755546	chr2:74425445-74426423	MTHFD2
cg13765939	chr22:27053082-27053629	MIAT
cg13768269	chr9:112917-114839	
cg13788515	chr2:207139337-207140031	ZDBF2
cg13850606	chr1:229542944-229543770	
cg13865488	chr9:137299191-137299437	RXRA
cg13877670	chr15:53080459-53083699	ONECUT1
cg13900100	chr6:43970074-43970922	c6orf223
cg13913015	chr2:47796924-47799166	KCNK12
cg13921956	chr2:12856626-12859019	TRIB2
cg13974773	chr6:27463177-27463379	
cg13984181	chr1:92495368-92495964	EPHX4

cg13996426	chr3:49755764-49757317	AMIGO3; GMPPB; RNF123
cg14002345	chr11:31831621-31839038	PAX6
cg14015044	chr8:22960385-22960927	TNFRSF1 C
cg14019323	chr12:65218246-65219143	TBC1D3
cg14024461	chr1:228645197-228646434	HIST3H2BB; HIST3H2A
cg14104369	chr20:57463653-57467739	GNAS
cg14117138	chr19:46800054-46800603	HIF3A
cg14126493	chr5:176830276-176831639	F12
cg14144513	chr11:69258150-69258544	
cg14159026	chr6:105584149-105585621	BVES; BVES-AS1
cg14169333	chr9:96720587-96723189	BARX1-DT
cg14178748	chr1:153650831-153652375	NPR1
cg14186641	chr6:88875398-88877449	CNR1
cg14216029	chr19:42901017-42901375	LIPE-AS1
cg14250130	chr10:3108651-3111419	PFKP
cg14251622	chr14:36002599-36005013	INSM2
cg14263942	chr4:76555367-76556079	CDKL2
cg14270220	chr12:49371691-49375550	WNT1
cg14287235	chr14:24803679-24804353	ADCY4
cg14306956	chr9:37026223-37028014	PAX5
cg14362312	chr9:136293567-136294160	ADAMTS13
cg14380270	chr17:33700488-33700760	SLFN11
cg14424538	chr20:1783825-1784552	
cg14493920	chr9:37034136-37038341	PAX5
cg14498666	chr9:21031514-21031823	HACD4
cg14543941	chr10:71077947-71079377	HK1
cg14547067	chr11:46316876-46317485	CREB3L1
cg14557534	chr3:27762352-27763995	EOMES
cg14572967	chr9:35616981-35617324	CD72
cg14592933	chr7:26415747-26416891	
cg14614314	chr10:135148197-135150565	CALY
cg14654324	chr9:137299191-137299437	RXRA
cg14654468	chr17:80186136-80189990	SLC16A3
cg14663510	chr10:124894076-124897366	HMX3
cg14670974	chr5:157098264-157099041	SOX3
cg14673387	chr10:47008085-47008410	GPRIN2
cg14676825	chr17:27038085-27038919	PROCA1
cg14681055	chr10:104000255-104001741	PITX3
cg14709460	chr7:28995306-28998541	TRIL
cg14743594	chr5:167956220-167957371	FBLL1
cg14776962	chr6:26251899-26252233	HIST1H2BH; H2BC9
cg14808890	chr6:26250437-26250827	HIST1H3F
cg14819504	chr2:160760605-160761452	LY75; LY75-CD3 2
cg14889643	chr18:60985504-60985741	BCL2
cg14897419	chr10:134120448-134122422	STK32C
cg14986699	chr2:149632683-149633882	KIF5C
cg14988503	chr4:76555367-76556079	CDKL2
cg14993712	chr7:138720045-138721019	ZC3HAV1L
cg14996783	chr7:28995306-28998541	TRIL
cg15017004	chr18:2905951-2907348	EMILIN2

cg15032314	chr19:50096610-50096912	PRR12
cg15044248	chr17:75368689-75370506	SEPTIN9
cg15104644	chr1:231296560-231297345	TRIM67
cg15110403	chr19:17392384-17393775	ANKLE1
cg15147516	chr1:226411008-226411880	MIXL1
cg15205527	chr13:45885755-45886103	
cg15264710	chr6:33396051-33396296	SYNGAP1
cg15267232	chr10:8091375-8098329	GATA3; GATA3-AS1
cg15336765	chr12:50354840-50356163	AQP5
cg15365320	chr11:17373020-17373665	NCR3LG1
cg15375239	chr19:38754847-38755699	SPINT2
cg15386964	chr6:26250437-26250827	HIST1H3F
cg15412498	chr5:71402878-71404868	MAP1B
cg15415545	chr16:30204696-30206485	[illegible]LA2B; SLX1A; SLX1A-SULT1[illegible]
cg15444947	chr7:28995306-28998541	TRIL
cg15459780	chr12:104609398-104610172	TXNRD1
cg15467646	chr1:22140892-22141407	LDLRAD2
cg15471815	chr1:228645197-228646434	HIST3H2BB; HIST3H2A
cg15490715	chr6:29521111-29521833	OR2I1P
cg15491911	chr6:167764523-167765124	
cg15531403	chr8:53851702-53854426	NPBWR1
cg15602740	chr3:120003884-120004426	GPR156
cg15607538	chr12:133484659-133485739	
cg15627078	chr13:24152900-24154140	TNFRSF19
cg15655500	chr18:12407716-12408242	SLMO1
cg15679813	chr22:45403037-45406372	PHF21B
cg15684724	chr8:67873389-67875600	TCF24
cg15720669	chr3:138663719-138666346	FOXL2; FOXL2NB
cg15732851	chr1:203598472-203598853	ATP2B4
cg15737319	chr2:74425445-74426423	MTHFD2
cg15760257	chr17:26698360-26699557	SARM1
cg15766101	chr19:46518284-46520080	CCDC61
cg15769764	chr4:995483-997541	IDUA
cg15775138	chr7:127744195-127744554	
cg15778437	chr11:31839364-31839813	PAX6
cg15835396	chr7:128809090-128809393	TSPAN33
cg15844835	chr5:141030919-141031177	FCHSD1
cg15866977	chr17:79917287-79920022	NOTUM
cg15875437	chr4:2059929-2063181	NAT8L
cg15969216	chr13:45149971-45152288	TSC22D1
cg15991309	chr17:80291021-80292394	SECTM1
cg15992535	chr5:139227606-139228279	NRG2
cg15994026	chr4:15779999-15780729	CD38
cg16008609	chr1:213123648-213125092	VASH2
cg16022575	chr1:47902794-47905518	FOXD2
cg16097357	chr17:73583839-73586337	MYO15B
cg16104915	chr7:27203916-27206462	HOXA9
cg16115720	chr1:208084099-208084513	CD34
cg16124981	chr19:9896564-9896994	ZNF846
cg16126280	chr5:158527375-158527983	AK123543

cg16128701	chr8:82192379-82193685	FABP5
cg16132520	chr7:134143116-134144063	AKR1B1
cg16215203	chr14:24803679-24804353	ADCY4
cg16219246	chr4:57396749-57397269	THEGL
cg16332936	chr22:19748651-19749189	TBX1
cg16358679	chr15:78556406-78557542	DNAJA4
cg16367511	chr2:74425445-74426423	MTHFD2
cg16390139	chr6:74161087-74162090	CGAS
cg16460359	chr15:72611947-72612802	CELF6
cg16475755	chr14:77964572-77965670	ISM2
cg16551483	chr17:4981358-4981979	ZFP3
cg16561266	chr17:62774367-62777797	[illegible]BPTFP1-KPNA2P3; ARHGAP2[illegible]
cg16580499	chr18:76737006-76741244	SALL3
cg16624692	chr11:65601070-65601551	SNX32
cg16633750	chr7:2558372-2559967	LFNG
cg16655905	chr19:38754847-38755699	SPINT2
cg16676492	chr15:34806491-34807382	
cg16686137	chr19:48837142-48837549	TMEM143
cg16732469	chr17:4981358-4981979	ZFP3
cg16734164	chr19:41316544-41317318	CYP2T1P
cg16775746	chr22:42353660-42353900	
cg16778903	chr15:72611947-72612802	CELF6
cg16779463	chr17:75368689-75370506	SEPTIN9
cg16820186	chr11:17373020-17373665	NCR3LG1
cg16857858	chr7:27212417-27214396	HOXA1 ; HOXA1 -HOXA9
cg16859924	chr17:20059029-20060060	SPECC1
cg16867584	chr19:49199965-49200184	FUT2
cg16882226	chr2:101033607-101034296	CHST1
cg16896847	chr8:144510851-144513957	MAFA
cg16915821	chr11:12029738-12030841	DKK3
cg16977570	chr13:24152900-24154140	TNFRSF19
cg16980360	chr17:29298047-29298606	RNF135
cg17003293	chr14:36002599-36005013	INSM2
cg17021218	chr10:88730555-88731632	AGAP11; ADIRF
cg17035412	chr7:26415747-26416891	
cg17054969	chr12:48397890-48398731	COL2A1
cg17088631	chr1:53098630-53099303	SHISAL2A
cg17172980	chr3:196755678-196757374	MELTF
cg17180977	chr1:228134423-228136282	WNT9A
cg17202086	chr11:77122737-77123088	PAK1
cg17222164	chr17:43339125-43339832	SPATA32; MAP3K14-AS1
cg17228900	chr6:391189-393790	IRF4
cg17246382	chr15:78556406-78557542	DNAJA4
cg17267805	chr10:44879715-44882391	CXCL12
cg17299935	chr17:74533282-74534566	PRCD; CYGB
cg17300544	chr17:75368689-75370506	SEPTIN9
cg17306261	chr14:65016517-65016909	PPP1R36
cg17321883	chr3:49755764-49757317	AMIGO3; GMPPB; RNF123
cg17373442	chr3:142837886-142840838	CHST2
cg17409893	chr19:42901017-42901375	LIPE-AS1

cg17419241	chr19:19738573-19739821	LAPAR2
cg17429382	chr19:3785512-3786653	MATK
cg17466857	chr7:27225051-27225629	HOXA11; HOXA11-AS
cg17567560	chr10:105036629-105038084	INA
cg17577122	chr22:19510872-19512254	CLDN5
cg17666539	chr19:7926591-7928752	EVI5L
cg17716617	chr1:33219428-33220028	KIAA1522
cg17737146	chr3:169529876-169530435	LRRC34
cg17757602	chr5:42951077-42952410	
cg17780956	chr4:156297602-156298094	MAP9
cg17802942	chr3:138663719-138666346	FOXL2; FOXL2NB
cg17812788	chr17:40332598-40333471	KCNH4
cg17853216	chr10:81002110-81003687	ZMIZ1
cg17858328	chr1:92945908-92952609	GFI1
cg17904852	chr3:48698336-48701667	CELSR3
cg17930034	chr14:65016517-65016909	PPP1R36
cg17960926	chr19:42901017-42901375	LIPE-AS1
cg17969084	chr7:27190275-27191115	HOXA-AS3
cg18049676	chr5:177540208-177541234	N4BP3
cg18118033	chr1:26686517-26687281	CRYBG2
cg18146843	chr1:53098630-53099303	SHISAL2A
cg18202336	chr15:99190447-99194559	IGF1R; IRAIN
cg18209835	chr7:6543016-6543598	GRID2IP
cg18210732	chr11:69517841-69519929	FGF19
cg18233405	chr8:98289605-98290404	TSPYL5
cg18245660	chr8:54791846-54795141	RGS2
cg18255595	chr13:20735044-20736157	GJA3
cg18260625	chr5:42951077-42952410	
cg18285309	chr1:40156931-40158514	HPCAL4
cg18309286	chr11:77122737-77123088	PAK1
cg18331004	chr20:1783825-1784552	
cg18339788	chr19:41641308-41642082	
cg18349835	chr7:158936508-158938492	VIPR2
cg18354742	chr19:38754847-38755699	SPINT2
cg18356974	chr7:128809090-128809393	TSPAN33
cg18368487	chr10:128993506-128995167	DOCK1
cg18409528	chr2:74740456-74743795	TLX2
cg18410627	chr9:129677707-129678009	RALGPS1
cg18423852	chr7:150652808-150653080	KCNH2
cg18424634	chr3:147126989-147128999	ZIC1
cg18429863	chr8:73448660-73449725	KCNB2
cg18456523	chr5:54516268-54516919	MCIDAS
cg18477204	chr14:70038109-70040302	CCDC177
cg18486102	chr12:50297581-50297988	FAIM2
cg18489434	chr14:77227570-77228691	VASH1
cg18514820	chr10:17270431-17272617	VIM; VIM-AS1
cg18558647	chr15:93198375-93199181	FAM174B
cg18588589	chr6:29521111-29521833	OR2I1P
cg18607529	chr7:50343758-50344519	IKZF1
cg18630040	chr6:46702737-46703316	PLA2G7

cg18638914	chr4:778662-780592	CPLX1
cg18675043	chr10:105452339-105453230	SH3PXD2A
cg18691800	chr6:17280740-17282334	RBM24
cg18696576	chr6:34202568-34206193	HMGA1
cg18716164	chr19:30015782-30021367	VSTM2B
cg18723937	chr7:37955623-37956555	SFRP4; EPDR1
cg18772588	chr20:61806255-61810867	MIR124-3
cg18786873	chr1:110610266-110613303	ALX3
cg18794404	chr10:22540708-22542739	
cg18798922	chr2:171569878-171573904	SP5
cg18818834	chr6:52226754-52228006	PAQR8
cg18853935	chr7:25896714-25896925	
cg18855096	chr9:124061806-124062229	GSN
cg18862481	chr3:129693128-129694841	TRH
cg18940113	chr2:161263872-161264460	RBMS1
cg19037922	chr4:108852496-108853387	CYP2U1
cg19045644	chr4:57975861-57976916	IGFBP7; IGFBP7-AS1
cg19127283	chr3:32858195-32860506	TRIM71
cg19155007	chr3:183542497-183543804	MAP6D1
cg19244428	chr6:3227026-3229688	TUBB2B
cg19250799	chr1:47909713-47911020	
cg19287823	chr6:155316300-155317197	TIAM2
cg19306047	chr19:19738573-19739821	LAPAR2
cg19337852	chr19:38754847-38755699	SPINT2
cg19355186	chr19:12305553-12306304	
cg19397703	chr19:43967248-43968625	LYPD3
cg19403104	chr19:50030982-50031300	RCN3
cg19424265	chr20:825268-826276	FAM11 A
cg19435033	chr17:19648140-19648492	ALDH3A1
cg19442495	chr3:142837886-142840838	CHST2
cg19504702	chr5:153862143-153862451	
cg19554255	chr17:75368689-75370506	SEPTIN9
cg19585816	chr8:41753341-41755250	ANK1
cg19590532	chr1:25255528-25259005	RUNX3
cg19595750	chr12:133065844-133066393	FBRSL1
cg19665644	chr11:13030331-13032584	RASSF1 ; RASSF1 -DT
cg19706516	chr1:151103686-151106100	SEMA6C
cg19714957	chr19:19843483-19843943	ZNF14
cg19722720	chr1:113050814-113052301	WNT2B
cg19734163	chr7:138720045-138721019	ZC3HAV1L
cg19752891	chr19:4304543-4305122	FSD1
cg19761115	chr5:178487147-178487921	ZNF354C
cg19763319	chr19:50316212-50316469	FUZ
cg19763809	chr2:98703355-98703889	VWA3B
cg19837790	chr10:97802872-97804262	CCNJ; ENTPD1-AS1
cg19850348	chr15:55879793-55881228	PYGO1
cg19861117	chr19:46456210-46456503	NOVA2
cg19863210	chr8:142138209-142139342	DENND
cg19875368	chr6:91320891-91321219	
cg19879075	chr10:73846807-73848233	SPOCK2

cg19900615	chr1:41267920-41268398	KCNQ4
cg19908577	chr4:115519423-115520932	UGT8
cg19929126	chr7:28995306-28998541	TRIL
cg19974428	chr7:98467024-98468058	TMEM13
cg20000718	chr4:41869175-41869459	
cg20011402	chr10:17495408-17496721	ST8SIA6
cg20018469	chr10:17270431-17272617	VIM; VIM-AS1
cg20056542	chr15:53080459-53083699	ONECUT1
cg20070026	chr14:102247661-102248279	PPP2R5C
cg20078466	chr7:50343758-50344519	IKZF1
cg20166714	chr12:113592204-113592620	CFAP73
cg20255775	chr12:48397890-48398731	COL2A1
cg20275528	chr17:75368689-75370506	SEPTIN9
cg20340508	chr21:34442207-34444543	OLIG1
cg20358834	chr11:66623621-66626614	LRNF4
cg20381020	chr11:66623621-66626614	LRNF4
cg20383948	chr21:46897842-46898158	COL18A1
cg20384898	chr6:1624186-1625468	GMDS
cg20399509	chr21:47717236-47718059	YBEY
cg20401551	chr22:20790639-20792665	SCARF2
cg20419623	chr20:48598960-48599657	SNAI1
cg20495645	chr22:45403037-45406372	PHF21B
cg20577019	chr1:150121696-150123078	PLEKHO1
cg20684180	chr3:38080629-38081187	DLEC1
cg20707679	chr19:3434930-3435417	NFIC
cg20744625	chr10:64574661-64578778	EGR2
cg20748955	chr11:118016238-118016984	SCN4B
cg20749741	chr2:263401-265238	SH3YL1; ACP1
cg20761860	chr5:131991555-131992304	IL13; TH2LCRR
cg20776829	chr4:90758009-90758870	SNCA
cg20797142	chr1:6051596-6052955	NPHP4; KCNAB2
cg20817902	chr4:81123509-81124318	PRDM8
cg20950465	chr1:53067881-53068608	GPX7
cg20961591	chr19:38754847-38755699	SPINT2
cg20971220	chr19:50554013-50554492	
cg21007414	chr11:76381450-76382295	LRRC32
cg21038156	chr7:158936508-158938492	VIPR2
cg21046967	chr17:37761692-37763551	NEUROD2
cg21074827	chr3:158288801-158289271	MLF1
cg21101720	chr17:27939299-27940770	ANKRD13B
cg21115608	chr13:20735044-20736157	GJA3
cg21144922	chr1:109203594-109204378	HENMET1
cg21158411	chr9:130689379-130690215	PIP5KL1
cg21195185	chr4:76555367-76556079	CDKL2
cg21249376	chr9:27528358-27528725	MOB3B
cg21277995	chr6:391189-393790	IRF4
cg21383810	chr12:65514879-65515863	WIF1
cg21384402	chr10:105036629-105038084	INA
cg21404045	chr7:27231806-27233097	
cg21424782	chr20:48598960-48599657	SNAI1

cg21425842	chr6:26240698-26240951	HIST1H4F
cg21447550	chr1:223536843-223538628	SUSD4
cg21463763	chr20:3713160-3713515	HSPA12B
cg21472506	chr2:63283937-63284147	OTX1
cg21497607	chr2:74425445-74426423	MTHFD2
cg21540810	chr19:53141176-53141813	ZNF83
cg21554552	chr3:50377804-50378540	RASSF1
cg21658235	chr8:22456092-22456508	c8orf58
cg21692846	chr11:134145560-134147180	GLB1L3
cg21773872	chr7:30721373-30722445	CRHR2
cg21889472	chr5:42992034-42992955	FLJ32255
cg21901946	chr7:127744195-127744554	
cg21908638	chr3:101497831-101498648	NXPE3
cg21940568	chr19:52207133-52207731	SPACA6
cg21962901	chr19:18543829-18549161	ISYNA1
cg22054918	chr4:81123509-81124318	PRDM8
cg22066230	chr11:66034752-66035054	RAB1B
cg22076972	chr19:6740670-6741203	TRIP1
cg22160000	chr1:45251963-45252292	BEST4
cg22168369	chr1:85462586-85463435	MCOLN2
cg22322863	chr17:30813178-30815239	CDK5R1
cg22345692	chr5:32709911-32714450	NPR3
cg22352818	chr2:97192978-97193383	
cg22367989	chr2:206546604-206547566	NRP2
cg22402852	chr4:108852496-108853387	CYP2U1
cg22409276	chr15:40763163-40764196	CHST14
cg22418737	chr7:139930066-139930356	
cg22447539	chr7:5632336-5634555	FSCN1
cg22465516	chr17:54910497-54912470	DGKE; c17orf67
cg22535307	chr15:48936811-48938577	FBN1
cg22546696	chr15:41952536-41953222	MGA
cg22740547	chr11:13030331-13032584	RASSF1 ; RASSF1 -DT
cg22762844	chr2:95690858-95692431	MAL
cg22789900	chr1:226411008-226411880	MIXL1
cg22791936	chr6:137242316-137245442	SLC35D3
cg22815953	chr20:48598960-48599657	SNAI1
cg22884130	chr22:32026298-32026874	PISD
cg22902177	chr3:129323916-129325332	PLXND1
cg22905274	chr4:154170369-154170600	TRIM2
cg22963915	chr19:3785512-3786653	MATK
cg22979783	chr2:72371122-72375004	CYP26B1
cg23003534	chr10:105036629-105038084	INA
cg23029526	chr1:21616381-21617101	ECE1
cg23052826	chr2:232478360-232479925	
cg23058177	chr2:10219803-10221016	CYS1
cg23125200	chr13:20806017-20806867	GJB6
cg23148701	chr12:64061837-64062774	DPY19L2
cg23152743	chr7:139167481-139168712	KLRG2
cg23229261	chr2:63283937-63284147	OTX1
cg23255835	chr2:29337984-29338909	CLIP4

cg23258881	chr1:150121696-150123078	PLEKHO1
cg23265500	chr14:65016517-65016909	PPP1R36
cg23297477	chr16:66638255-66639561	CMTM3
cg23305567	chr19:54369388-54369809	MYADM
cg23337116	chr4:53728038-53729000	RASL11B
cg23426238	chr7:103086345-103086840	SLC26A5
cg23428985	chr2:29337984-29338909	CLIP4
cg23511613	chr2:88751336-88752865	FOXI3
cg23528400	chr3:32858195-32860506	TRIM71
cg23620340	chr1:63782395-63790471	FOXD3; FOXD3-AS1
cg23634087	chr20:50721311-50721912	ZFP64
cg23684074	chr7:128470444-128471161	FLNC
cg23728669	chr11:63258379-63258804	HRASLS5
cg23735712	chr4:40858885-40859162	APBB2
cg23754431	chr11:69258150-69258544	
cg23782001	chr7:138720045-138721019	ZC3HAV1L
cg23837799	chr15:31683985-31685824	
cg23844174	chr1:2064629-2064855	PRKCZ
cg23874561	chr4:81124469-81124845	PRDM8
cg23912429	chr10:17270431-17272617	VIM; VIM-AS1
cg23923856	chr1:113050814-113052301	WNT2B
cg23965061	chr8:22960385-22960927	TNFRSF1 C
cg24031355	chr10:22634001-22634862	SPAG6
cg24037897	chr10:16561605-16563822	C1QL3
cg24078363	chr14:77227570-77228691	VASH1
cg24084504	chr4:42399153-42400802	SHISA3
cg24098326	chr10:123922851-123923542	TACC2
cg24102266	chr1:33219428-33220028	KIAA1522
cg24106636	chr1:116380360-116382364	NHLH2
cg24159214	chr3:50402104-50402942	CACNA2D2
cg24166864	chr12:65514879-65515863	WIF1
cg24217704	chr2:166649910-166650966	GALNT3
cg24217844	chr6:137242316-137245442	SLC35D3
cg24276681	chr18:2905951-2907348	EMILIN2
cg24300924	chr18:12407716-12408242	SLMO1
cg24331301	chr10:73156165-73157954	CDH23
cg24368902	chr10:43248524-43250912	
cg24385580	chr17:28088028-28088607	SSH2
cg24393316	chr9:100615235-100617510	FOXE1
cg24405179	chr1:226411008-226411880	MIXL1
cg24412501	chr11:13689589-13690724	FAR1; FAR1-IT1
cg24432073	chr4:76555367-76556079	CDKL2
cg24448269	chr17:54910497-54912470	DGKE; c17orf67
cg24525457	chr7:100091181-100091598	NYAP1
cg24542751	chr17:43339125-43339832	SPATA32; MAP3K14-AS1
cg24588482	chr1:160370113-160370658	VANGL2
cg24599205	chr1:110752257-110754794	KCNC4
cg24610236	chr7:30721373-30722445	CRHR2
cg24626079	chr12:4378367-4382222	CCND2; CCND2-AS1
cg24662718	chr1:108507252-108507841	VAV3; VAV3-AS1

cg24685006	chr1:36042433-36043444	TFAP2E
cg24718866	chr2:25391073-25391875	POMC
cg24722112	chr3:49755764-49757317	AMIGO3; GMPPB; RNF123
cg24724633	chr19:36909282-36909854	ZFP82
cg24740531	chr18:60985504-60985741	BCL2
cg24746100	chr2:88751336-88752865	FOXI3
cg24800655	chr12:133065844-133066393	FBRSL1
cg24804544	chr7:6543796-6544108	GRID2IP
cg24816460	chr6:4775132-4777550	CDYL
cg24856258	chr15:41952536-41953222	MGA
cg24876960	chr5:1881925-1887743	IRX4; CTD2194D22.4
cg24886267	chr5:167956220-167957371	FBLL1
cg24940138	chr17:41363728-41364273	TMEM1 6A
cg25109009	chr16:69760294-69760506	NQO1
cg25137711	chr1:48058794-48059230	
cg25152631	chr3:140660335-140661602	SLC25A36
cg25253217	chr19:47776371-47778740	CCDC9; INAFM1
cg25287474	chr9:14693031-14693576	ZDHHC21
cg25307318	chr10:123922851-123923542	TACC2
cg25331703	chr15:101458287-101460223	LRRK1
cg25335544	chr3:49906738-49907482	CAMKV
cg25340966	chr1:119531992-119532196	TBX15
cg25344734	chr11:13030331-13032584	RASSF1
cg25375340	chr1:85462586-85463435	MCOLN2
cg25466090	chr1:2064629-2064855	PRKCZ
cg25486143	chr3:50377804-50378540	RASSF1
cg25513173	chr15:83875649-83877079	HDGFL3
cg25524962	chr13:20735044-20736157	GJA3
cg25528916	chr19:4328659-4328865	STAP2
cg25577023	chr14:103394398-103397070	AMN
cg25622366	chr2:63281035-63281347	OTX1
cg25623934	chr2:12856626-12859019	TRIB2
cg25645687	chr3:138663719-138666346	FOXL2; FOXL2NB; LINC 1391
cg25693302	chr18:55862654-55862873	NEDD4L
cg25694349	chr12:133065844-133066393	FBRSL1
cg25714826	chr16:85783863-85785131	C16orf74
cg25723050	chr1:224803638-224805314	CNIH3
cg25737323	chr2:29337984-29338909	CLIP4
cg25741023	chr10:102891011-102891794	TLX1
cg25764899	chr1:47696505-47698197	TAL1
cg25823419	chr3:179168736-179169593	GNB4
cg25830696	chr17:42030174-42030941	PYY
cg25848557	chr1:108507252-108507841	VAV3; VAV3-AS1
cg25862644	chr10:77155129-77169600	ZNF5 3; ZNF5 3-AS2
cg25876509	chr7:97501184-97501919	ASNS
cg25881804	chr20:21080930-21082824	
cg25886284	chr19:36909282-36909854	ZFP82
cg25945732	chr2:263401-265238	SH3YL1; ACP1
cg25947878	chr2:20068429-20068907	LINC 954
cg26005578	chr11:13030331-13032584	RASSF1

cg26009486	chr14:24837873-24838324	NFATC4
cg26013553	chr1:111216245-111217937	KCNA3
cg26015401	chr19:49436696-49437055	DHDH
cg26029345	chr8:22960385-22960927	TNFRSF1 C
cg26043257	chr4:15779999-15780729	CD38
cg26107850	chr1:228645197-228646434	HIST3H2A; HIST3H2BB
cg26107890	chr3:124860570-124861019	SLC12A8
cg26117023	chr2:74781495-74782685	DOK1; LOXL3
cg26173997	chr4:76555367-76556079	CDKL2
cg26195583	chr4:41869175-41869459	
cg26211360	chr12:122667649-122668038	LRRC43
cg26240185	chr11:13689589-13690724	FAR1
cg26245531	chr14:88459119-88459689	GALC
cg26246928	chr1:153650831-153652375	NPR1
cg26253438	chr1:203598472-203598853	ATP2B4
cg26259171	chr19:49255779-49256495	FUT1
cg26261793	chr1:171810468-171811325	DNM3
cg26279550	chr12:66582696-66583345	IRAK3
cg26281453	chr17:45810590-45811388	TBX21
cg26301389	chr5:139047906-139048235	CXXC5
cg26322231	chr7:138720045-138721019	ZC3HAV1L
cg26386852	chr3:48693119-48694768	CELSR3
cg26397188	chr12:65514879-65515863	WIF1
cg26415547	chr12:66582696-66583345	IRAK3
cg26425933	chr10:115999009-115999603	VWA2
cg26448406	chr22:32026298-32026874	PISD
cg26460092	chr15:83775862-83776922	TM6SF1
cg26468478	chr3:48693119-48694768	CELSR3
cg26470101	chr2:173099483-173100098	
cg26526379	chr7:26415747-26416891	
cg26570179	chr8:99960498-99961438	OSR2
cg26575690	chr15:34728788-34729495	
cg26620530	chr12:115102688-115103982	
cg26714410	chr13:45149971-45152288	TSC22D1; TSC22D1-AS1
cg26742995	chr17:43339125-43339832	SPATA32
cg26797073	chr2:131129719-131130511	PTPN18
cg26802291	chr19:19371676-19372393	HAPLN4
cg26857670	chr7:27225051-27225629	HOXA11-AS; HOXA11
cg26870803	chr11:66623621-66626614	LRFN4; PC
cg26919818	chr5:177540208-177541234	N4BP3
cg26928125	chr11:69517841-69519929	FGF19
cg26953749	chr12:122667649-122668038	LRRC43
cg26954625	chr19:19738573-19739821	LAPAR2
cg26983469	chr10:17270431-17272617	VIM; VIM-AS1
cg26998044	chr17:8868470-8869372	PIK3R5
cg27018380	chr14:93389246-93389899	CHGA
cg27028760	chr16:2041826-2043026	SYNGR3
cg27071460	chr5:40679503-40682081	PTGER4; TTC33
cg27085741	chr7:143042509-143042867	CLCN1
cg27118059	chr21:32929928-32932017	TIAM1; AP 251.1

cg27127017	chr12:113592204-113592620	CCDC42B
cg27212234	chr19:36048557-36049673	ATP4A
cg27239157	chr3:183145428-183146790	MCF2L2
cg27304406	chr15:83951981-83953930	BNC1
cg27306119	chr2:9143128-9144630	MBOAT2
cg27382405	chr12:49942787-49943435	KCNH3
cg27395939	chr11:17373020-17373665	NCR3LG1
cg27452341	chr7:143042509-143042867	CLCN1
cg27506082	chr2:207139337-207140031	ZDBF2
cg27513979	chr13:96742139-96744162	HS6ST3
cg27558479	chr2:74425445-74426423	MTHFD2
cg27587125	chr1:109203594-109204378	HENMT1
cg27605748	chr5:42951077-42952410	
cg27633530	chr7:50343758-50344519	IKZF1
cg27636310	chr1:46632690-46632932	PIK3R3

Table S3

CpG ID	CpG site (hg19)_Illumina 450K Array	CpG site (hg19)	CGI (hg19)	Gene name	Gene complete name	Genomic region
CpG#1	cg22789900	chr1:226411715	chr1:226411008-226411880	*MIXL1*	Mix Paired-Like Homeobox	Gene body
CpG#2	cg11173146	chr13:107187413	chr13:107186469-107189024	*EFNB2*	Ephrin B2	Promoter
CpG#3	cg07689503	chr2:74426201	chr2:74425445-74426423	*MTHFD2*	Methylenetetrahydrofolate Dehydrogenase 2	Gene body
CpG#4	cg17003293	chr14:36003826	chr14:36002599-36005013	*INSM2*	INSM Transcriptional Repressor 2	Gene body
CpG#5	cg15386964	chr6:26250686	chr6:26250437-26250827	*HIST1H2BH;HIST1H3F*	Histone Cluster 1, H3f	Promoter
CpG#6	cg04907523	chr1:213124896	chr1:213123648-213125092	*VASH2*	Vasohibin 2	Gene body
CpG#7	cg16896847	chr8:144512042	chr8:144510851-144513957	*MAFA*	MAF BZIP Transcription Factor A	Gene body
CpG#8	cg15969216	chr13:45150263	chr13:45149971-45152288	*TSC22D1*	Transforming growth factor-beta-stimulated clone-22	Promoter
CpG#9	cg08296831	chr11:65554175	chr11:65553750-65555573	*OVOL1*	Ovo Like Transcriptional Repressor 1	Promoter
CpG#10	cg08042316	chr7:139930256	chr7:139930066-139930356			Intergenic
CpG#11	cg20776829	chr4:90758797	chr4:90758009-90758870	*SNCA*	Synuclein Alpha	Promoter
CpG#12	cg17567560	chr10:105036863	chr10:105036629-105038084	*INA*	Internexin Neuronal Intermediate Filament Protein Alpha	Promoter
CpG#13	cg06341513	chr11:61277017	chr11:61275826-61277329	*LRRC10B*	Leucine Rich Repeat Containing 10B	Gene body
CpG#14	cg27636310	chr1:46632696	chr1:46632690-46632932	*PIK3R3*	Phosphoinositide-3-Kinase Regulatory Subunit 3	Gene body
CpG#15	cg25830696	chr17:42030480	chr17:42030174-42030941	*PYY*	Peptide Tyrosine Tyrosine	Gene body
CpG#16	cg14996783	chr7:28996903	chr7:28995306-28998541	*TRIL*	TLR4 Interactor With Leucine Rich Repeats	Gene body
CpG#17	cg05040544	chr13:107188257	chr13:107186469-107189024	*EFNB2*	Ephrin B2	Promoter
CpG#18	cg01939477	chr11:43602880	chr11:43602546-43603215	*MIR129-2*	MicroRNA 129-2	Promoter
CpG#19	cg26468478	chr3:48693598	chr3:48693119-48694768	*CELSR3*	Cadherin EGF LAG Seven-Pass G-Type Receptor 3	Gene body
CpG#20	cg17466857	chr7:27225528	chr7:27225051-27225629	*HOXA11, HOXA11-AS*	Homeobox A11; HOXA11 Antisense RNA	Gene body
CpG#21	cg04321866	chr13:107188417	chr13:107186469-107189024	*EFNB2*	Ephrin B2	Promoter
CpG#22	cg07435294	chr11:65554356	chr11:65553750-65555573	*OVOL1*	Ovo Like Transcriptional Repressor 1	Promoter
CpG#23	cg13325154	chr10:104001240	chr10:104000255-104001741	*PITX3*	Paired Like Homeodomain 3	Promoter
CpG#24	cg16460359	chr15:72612630	chr15:72611947-72612802	*CELF6*	CUGBP Elav-Like Family Member 6	Promoter
CpG#25	cg11672054	chr1:228645482	chr1:228645197-228646434	*HIST3H2A, HIST3H2BB*	Histone cluster 3 H2A; Histone Cluster 3, H2bb	Promoter
CpG#26	cg04281464	chr14:70014874	chr14:70014544-70014993			Intergenic
CpG#27	cg18456523	chr5:54516805	chr5:54516268-54516919	*MCIDAS*	Multiciliate Differentiation And DNA Synthesis Associated Cell Cycle Protein	Gene body
CpG#28	cg17300544	chr17:75369091	chr17:75368689-75370506	*SEPT9*	Septin 9	Promoter
CpG#29	cg03468349	chr2:264164	chr2:263401-265238	*SH3YL1*	SH3 And SYLF Domain Containing 1	Promoter
CpG#30	cg05327835	chr4:996175	chr4:995483-997541	*IDUA*	Alpha-L-Iduronidase	Gene body
CpG#31	cg11595545	chr1:111217497	chr1:111216245-111217937	*KCNA3*	Potassium Voltage-Gated Channel Subfamily A Member 3	Promoter
CpG#32	cg13204512	chr17:29298184	chr17:29298047-29298606	*RNF135*	Ring Finger Protein 135	Promoter
CpG#33	cg03063639	chr15:83776423	chr15:83775862-83776922	*TM6SF1*	Transmembrane 6 Superfamily Member 1	Promoter
CpG#34	cg04823311	chr7:28997486	chr7:28995306-28998541	*TRIL*	TLR4 Interactor With Leucine Rich Repeats	Gene body
CpG#35	cg25577023	chr14:103394900	chr14:103394398-103397070	*AMN*	Amnion Associated Transmembrane Protein	Gene body
CpG#36	cg00922376	chr1:14026585	chr1:14026482-14027200	*PRDM2*	PR/SET Domain 2	Promoter
CpG#37	cg14159026	chr6:105584552	chr6:105584149-105585621	*BVES*	Blood Vessel Epicardial Substance	Promoter
CpG#38	cg17373442	chr3:142839991	chr3:142837886-142840838	*CHST2*	Carbohydrate Sulfotransferase 2	Gene body
CpG#39	cg20275528	chr17:75369485	chr17:75368689-75370506	*SEPT9*	Septin 9	Promoter

Supplementary Table S4

		n samples	
Study		NTs (>5)	Tumor
LIHC	Liver Hepatocellular Carcinoma	50	380
BLCA	Bladder Urothelial Carcinoma	21	413
BRCA	Breast Invasive Carcinoma	98	790
CHOL	Cholangiocarcinoma	9	36
COADREAD	Colorectal Adenocarcinoma	45	398
ESCA	Esophageal Carcinoma	16	186
HNSC	Head and Neck Squamous Cell Carcinoma	50	530
KIRC	Kidney Renal Clear Cell Carcinoma	160	320
LUAD	Lung Adenocarcinoma	32	460
LUNG	Lung Squamous Cell Carcinoma	74	833
LUSC	Lung Squamous Cell Carcinoma	43	372
PAAD	Pancreatic Adenocarcinoma	10	185
PRAD	Prostate Adenocarcinoma	49	500
THCA	Thyroid Carcinoma	56	515
UCEC	Uterine Corpus Endometrial Carcinoma	46	432
ACC	Adrenocortical Carcinoma		80
CESC	Cervical Squamous Cell Carcinoma and Endocervical Adenocarcinoma		312
DLBC	Diffuse Large B-Cell Lymphoma		48
GBM	Glioblastoma Multiforme		153
LAML	Acute Myeloid Leukemia		194
MESO	Mesothelioma		87
OV	Ovarian Serous Cystadenocarcinoma		10
SARC	Sarcoma		265
SKCM	Skin Cutaneous Melanoma		474
STAD	Stomach Adenocarcinoma		396
TGCT	Testicular Germ Cell Tumors		156
THYM	Thymoma		124
UCS	Uterine Carcinosarcoma		57
	**TOTAL**	**759**	**8706**
